# Unveiling the neuroinflammatory potential of aspartame in Alzheimer’s disease: A comprehensive network toxicology and experimental validation approach

**DOI:** 10.1371/journal.pone.0361766

**Published:** 2026-11-03

**Authors:** Dongyang Li, Xuejun Weng, Yijuan Huang, Dongni Yang, Shunhan Yao, Weihao Zheng, Xiaohong Jiang, Yilin Wei, Sheng Xu, Chunwei Wei

**Affiliations:** 1 School of Nursing, Guangxi Medical University, Nanning, China; 2 Department of Neurology, The Second Affiliated Hospital of Guangxi Medical University, Nanning, China; 3 Guangxi Key Laboratory of Artificial Intelligence for Genetic Diseases of Long-dwelling Nationalities, Youjiang Medical University for Nationalities, Baise, China; 4 Institute of Life Sciences, Guangxi Medical University, Nanning, China; 5 Department of Orthopedic, Affiliated Minzu Hospital of Guangxi Medical University, Nanning, China; Lusofona University of Humanities and Technologies: Universidade Lusofona de Humanidades e Tecnologias, PORTUGAL

## Abstract

Aspartame, a widely used artificial sweetener, has raised significant concerns regarding its safety, especially in light of the rising incidence of Alzheimer’s Disease (AD). However, systematic investigations into aspartame’s potential neurotoxicity and its role in AD pathology remain limited. In this study, we employed an integrated approach combining network toxicology predictions, molecular docking, and in vitro validation to explore the neurotoxic mechanisms of aspartame in AD. By analyzing multiple public databases through network toxicology, we identified 185 common targets between aspartame and AD. Protein-protein interaction (PPI) network analysis further highlighted 10 core hub genes, including KDR, CCNE1, and GSK-3β. Molecular docking predicted favorable interactions for key targets such as GSK-3β (−5.002 kcal/mol) and KDR (−5.613 kcal/mol), with other hubs showing moderate binding affinities. In vitro experiments demonstrated that aspartame dose-dependently reduced BV2 microglial cell viability and triggered a pro-inflammatory response. Specifically, aspartame increased the production of nitric oxide (NO) and pro-inflammatory cytokines (TNF-α, IL-1β, IL-6), while upregulating the expression of GSK-3β, iNOS, TNF-α, IL-1β, and IL-6, similar to LPS-induced inflammation. These findings suggest that aspartame may induce a pro-inflammatory response in BV2 microglial cells through multiple signaling pathways, although the relevance of these findings to human neurological safety remains to be established. This study provides a basis for further investigation into the neurological safety of aspartame and proposes a novel paradigm for evaluating the neurotoxic potential of food additives using an integrated systems toxicology approach.

## Introduction

Alzheimer’s disease (AD) is one of the most common neurodegenerative diseases, with patients typically presenting with memory loss, cognitive impairment, and a deterioration in the ability to perform daily activities [1]. According to the World Health Organization (WHO), more than 50 million people worldwide are living with AD, and this number is expected to increase substantially in the coming decades due to the accelerating aging of the global population [2,3]. The pathogenesis of AD is complex and remains incompletely understood, involving the aggregation of β-amyloid peptides [4], abnormal phosphorylation of tau protein [5], and neuroinflammation [6]. However, no effective drug has been found to cure the disease so far. Recent studies have revealed a close association between dietary components and disease onset [7]. Aspartame is a widely used artificial sweetener commonly incorporated into food additives, has been the subject of extensive debate regarding its potential effects on human health and safety [8].

Aspartame is a low-calorie sweetener widely used in sugar-free or low-sugar foods and beverages to modulate palatability, and it has been approved for use by regulatory authorities in numerous countries and regions [9]. In recent years, increasing attention has been directed toward the potential toxicological effects and adverse health outcomes associated with aspartame exposure. Previous studies have demonstrated that aspartame exposure can promote triglyceride and phospholipid biosynthesis in neuronal cells, accompanied by abnormal lipid droplet accumulation [10], the addition of aspartame or its metabolites to neurons can induce oxidative stress and mitochondrial damage, in response to which neurons synthesize triglycerides and store them in lipid droplets as a protective mechanism [11]. Additionally, aspartame has been reported to activate NMDA and AMPA receptors, increase intracellular calcium influx, and trigger reactive oxygen species (ROS) production, which may collectively aggravate neurodegenerative progression [12].

Animal exposure to aspartame shows marked decreases in spatial working and recognition memory, together with inhibition of the NMDAR–CaMKII–ERK/CREB signaling pathway, suggesting that this inhibition leads to neuronal excitotoxicity and memory decline [13]. Furthermore, aspartame metabolites, including phenylalanine, aspartate, and methanol, are capable of crossing the blood–brain barrier and accumulating within the central nervous system [14], potentially perturbing neurotransmitter balance, eliciting neurotoxic reactions, and compromising neural function [9]. Despite accumulating evidence implicating aspartame in potential neurotoxicity, most previous studies have focused on individual biological effects rather than systematic mechanisms. Consequently, the molecular links between aspartame exposure and AD pathology remain poorly understood, and the toxicological mechanisms underlying its involvement in AD warrant further investigation [15].

In recent years, multi-target prediction approaches have been increasingly applied in drug discovery and the investigation of disease mechanisms [16]. The construction of AD-associated target networks, combined with drug-target interaction analysis and multi-target prediction strategies, can provide valuable insights into AD pathogenesis and facilitate the identification of potential therapeutic candidates [17,18]. Further evaluation of target interactions through functional enrichment analysis and molecular docking approaches may help elucidate the potential pathways and molecular mechanisms underlying the effects of aspartame on AD [19,20].

In this study, network toxicology, bioinformatics, and molecular docking methodologies were integrated to explore the putative involvement of aspartame in the pathology of AD (Scheme 1). We identified 185 putative targets linking aspartame to AD through integrative analysis of multiple databases. A protein–protein interaction (PPI) network was constructed, followed by GO and KEGG pathway enrichment analyses to identify pathways significantly associated with AD. Molecular docking simulations were subsequently performed to predict the possible binding interactions between aspartame and its targets. Finally, in vitro assays were conducted to assess the impact of aspartame on BV2 microglial cell viability and the expression of key inflammatory cytokines, including TNF-α, IL-1β, and IL-6. By combining systematic network toxicology with multi-faceted molecular validation, this study seeks to explore the potential mechanisms linking aspartame to AD and to identify candidate targets for further investigation.

## Materials and methods

### Aspartame target collection

The canonical SMILES and standard structure of aspartame were determined through a search in the PubChem database. Potential targets of aspartame were predicted by employing multiple databases, including ChEMBL (Version 3313) (https://www.ebi.ac.uk/chembl/), STITCH (Version 5.0) (http://stitch.embl.de/), Swiss Target Prediction (https://swisstargetprediction.ch/), Similarity Ensemble Approach, and PharmMapper (Version 2017) (https://www.lilab-ecust.cn/pharmmapper/index.html), with the keyword “aspartame” and species restriction to Homo sapiens. The structures of the search results were examined, and consistency comparisons were conducted. Data integration and duplicate removal were performed for the potential targets, including ChEMBL numbers and STITCH codes. Target name standardization was carried out using the UniProt database.

### Collection of AD targets

AD-related targets were collected by querying the GeneCards (Version 5.19) (https://www.genecards.org/), OMIM (https://www.omim.org/), and DisGeNET (v7.0) (https://disgenet.com/) databases with the term “AD.”

### Screening of core targets and construction of PPI networks

Data regarding potential associations between aspartame exposure and AD were input into the STRING database (Version 12.0). The analysis was restricted to Homo sapiens, a minimum required interaction score of 0.9 (highest confidence) was applied, and disconnected nodes were excluded. Subsequently, we imported the STRING-derived information into Cytoscape (v3.10.1) to construct a protein-protein interaction (PPI) network. Core targets were then identified based on their degree values using the cytoHubba plugin. The average degree of the network was calculated as 2L/N, where L is the number of edges and N is the number of nodes.

### Functional analysis of target proteins and pathway enrichment

To investigate the biological functions of potential targets overlapping between aspartame and AD, we performed Gene Ontology (GO) and Kyoto Encyclopedia of Genes and Genomes (KEGG) pathway enrichment analyses using the DAVID database (v2023q4). The GO analysis encompassed biological process (BP), cellular component (CC), and molecular function (MF), allowing us to delineate the principal biological roles of the associated targets. Key pathways overlapping aspartame- and AD-associated targets were determined via KEGG enrichment analysis. A Benjamini–Hochberg adjusted p-value < 0.05 was used as the significance threshold to control the false discovery rate (FDR), and all visualizations were performed using the SRplot platform.

### Microarray data analysis

To validate the findings of this study, two microarray datasets (GSE157827) were obtained from the NCBI Gene Expression Omnibus (GEO) database, a public repository of high-throughput gene expression data. Differential expression between AD and healthy tissues was analyzed using GEO2R. Volcano plots were generated via the SRplot platform to visualize differentially expressed genes (DEGs), applying selection criteria of corrected p-value <0.05 and |log_2_ (fold change)| ≥ 1. The resulting DEGs were then compared with the identified hub genes, and the overlapping genes were selected for further investigation.

### Molecular docking analysis

To evaluate the binding affinity and interaction patterns between aspartame and its targets, we employed AutoDock Vina 1.2.2. The molecular structure of aspartame was obtained from the PubChem compound database (https://pubchem.ncbi.nlm.nih.gov/), and the protein structures of CCNE1, CDKN1A, CDKN1B, ERBB2, GSK3B, KDR, NOS3, and VEGFA were downloaded from the Protein Data Bank (PDB). We first converted both protein and ligand files to PDBQT format, during which water molecules were removed and polar hydrogen atoms were added. The grid box was centered to encompass the structural domains of each protein and to allow unrestricted molecular movement. The docking search space was set as a cubic space of 30 Å × 30 Å × 30 Å, with grid points spaced 0.05 nm apart. Molecular docking calculations were then carried out using AutoDock Vina 1.2.2.

### Cell viability assay

Cell viability was evaluated using the CCK-8 assay kit (Beyotime Biotechnology, China), strictly following the manufacturer’s instructions. BV2 murine microglial cells were maintained in Dulbecco’s Modified Eagle Medium (DMEM; Gibco) supplemented with 10% fetal bovine serum (FBS; Gibco) and incubated at 37 °C in a humidified atmosphere containing 5% CO_2_. BV2 cells were seeded at 1 × 10^4^ cells/well in 96-well plates, and cells were co-cultured with aspartame at concentrations ranging from 0.05 to 5 mg/mL for 24 hours. Then, the supernatant was replaced with 10% CCK-8 solution, and the plates were incubated at 37 °C for 0.5 h. Absorbance was then measured at 450 nm using a microplate reader.

### Detection of NO, TNF-α, IL1 β, and IL6

Cell treatment and inflammatory factor measurements were carried out in accordance with previous study [21]. BV2 cells were seeded at 1 × 10^4^ cells/well in regular cell culture dishes and treated with either LPS (100 ng/mL) or aspartame (1 mg/mL) for 12 hours. Thereafter, the culture supernatants were collected from each group. The levels of TNF-α (Solarbio, SEKM-0034), IL-6 (Solarbio, SEKM-0007), and IL-1β (Solarbio, SEKM-0002) were quantified by ELISA. Nitric oxide (NO) (Solarbio, BC1475) was determined using a nitric oxide assay kit (Solarbio, BC1475).

### qPCR analysis

Total RNA was isolated from cultured BV2 cells using Trizol (Invitrogen). Total RNA (1 μg) was reverse-transcribed into cDNA using the PrimeScript^TM^ RT reagent kit with gDNA Eraser (RR 047 A, TaKaRa, Otsu, Shiga, Japan). qPCR was performed using TB Green® Premix Ex Taq^TM^ (Tli RNaseH Plus) (RR820A, TaKaRa, Otsu, Shiga, Japan). PCR was conducted on a CFX 96^TM^ optical reaction module (Bio-Rad, Hercules, CA, USA). The primer sequences employed for qPCR are provided in Table 1.

**Table 1:** Primer sequences used for quantitative real-time PCR.

Gene	Sequences (5′-3′)
TNF-α	F: ATGTCTCAGCCTCTTCTCATTC
	R: GCTTGT CACTCGAATTTTGAGA
IL-1β	F: CACTACAGGCTCCGAGATGAACAAC
	R: TGTCGTTGCTTGGTTCCTCTTGTAC
IL-6	F: CTCCCAACAGACCTGTCTATAC
	R: CCATTGCACAACTCTTTTTCTCA
GSK-3β	F: 5′-TCGCTCAAGGTCACAAGAAAA-3′
	R:5′-CATCAGAGGAAGGAGAAAAC-3′
iNOS	F: 5′-ATCTTGGAGCGAGTTGTGGATTGTC-3′
	R:5′-TAGGTGAGGGCTTGGCTGAG-3′
IBA-1	F: 5′-ATCAACAAGCAATTCCTCGATGA
	R:5′-CAGCATTCGCTTCAAGGACATA
β-actin	F: 5’-GGCTGTATTCCCCTCCATCG-3’
	R:5’-TCCTATGGGAGAACGGCAGA-3’

### Statistical analysis

Results are expressed as mean ± standard deviation. Data analysis was performed using GraphPad Prism 5.0, and statistical comparisons between groups were conducted using one-way analysis of variance (one-way ANOVA with Dunnett’s post-hoc test). A value of P < 0.05 was considered statistically significant. P values reported are nominal and not adjusted for multiplicity across different endpoints. No formal correction for multiple comparisons was applied. Given that the qPCR experiments included three biological replicates and no formal power analysis was performed, these data should be regarded as exploratory and hypothesis-generating rather than confirmatory.

## Results

### Analysis of potential targets of aspartame and AD

We initially screened 218 aspartame-related targets from the ChEMBL, STITCH, SwissTargetPrediction, PubChem, and PharmMapper databases. Simultaneously, 33,284 AD-associated targets were retrieved from GeneCards, OMIM, and DisGeNET. After integrating these target sets and removing duplicates, we obtained 185 overlapping targets, which were considered potential targets relevant to both aspartame and AD. These results indicate that AD, as a complex neurological disorder, manifests broad molecular network perturbations and further imply that aspartame may modulate the nervous system through multiple signaling pathways.

### Identification of core targets and construction of the potential target interaction network

A protein-protein interaction (PPI) network was constructed using the STRING database with a confidence threshold set at 0.9. Among the 185 overlapping targets, 42 lacked interaction partners meeting this high-confidence criterion and were excluded as disconnected nodes. The resulting network comprised 143 nodes and 231 edges, with an average degree of 3.23 (calculated as 2 × 231 / 143). Cytoscape software was used to visualize the PPI network (Fig 1), which illustrates the specific connections among the 10 core AD-related targets identified through network analysis.

**Fig 1:**
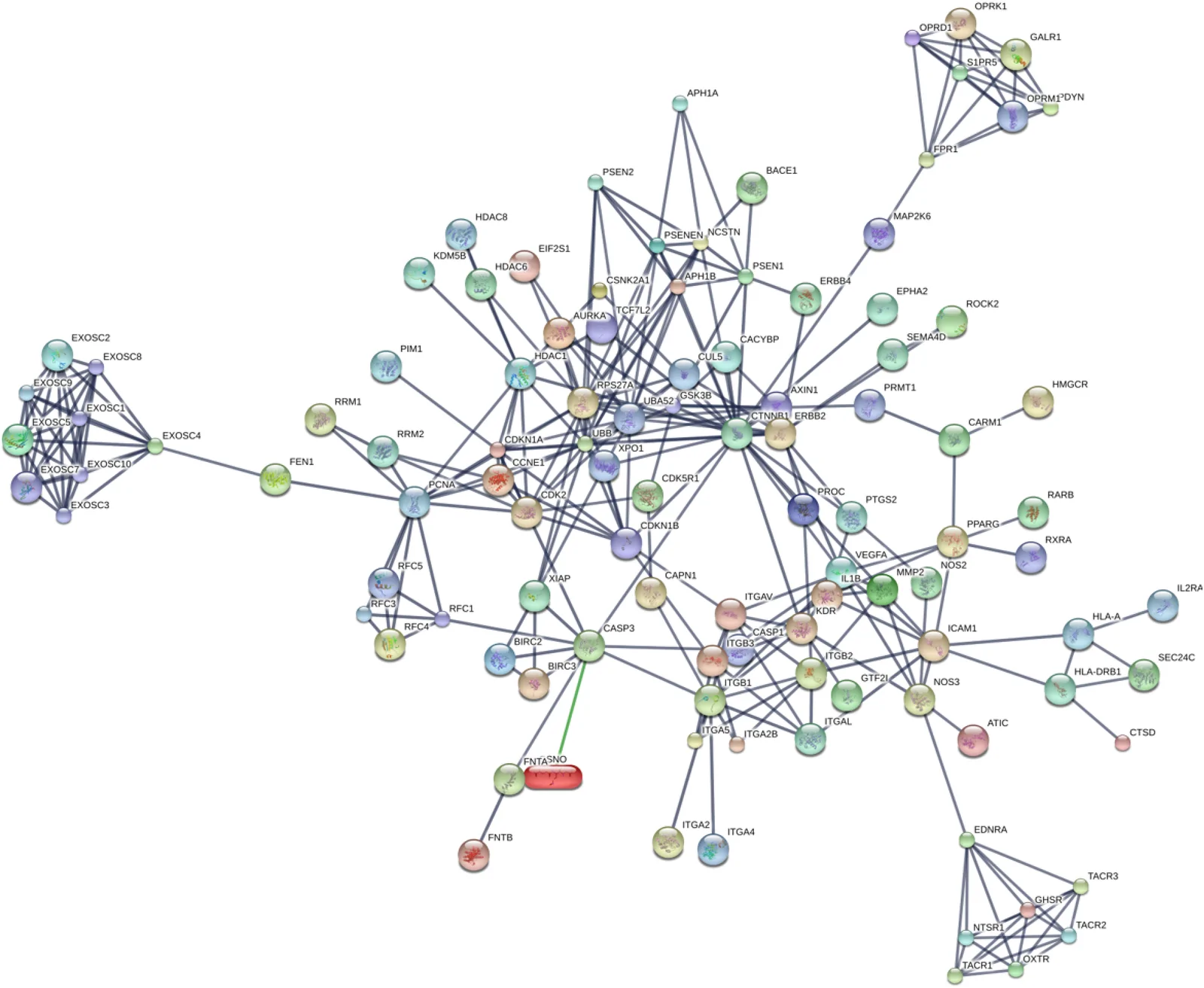
The PPI network of the potential targets and the core targets.

### GO functional enrichment analysis

GO analysis was performed on the 143 potential targets implicated in aspartame’s effects on AD (restricted to Homo sapiens), yielding 293 statistically significant GO terms; 196 BP terms, 56 CC terms, and 41 MF terms. GO terms were ranked by p-value, and the top 10 terms in each category were selected for visualization (Fig 2). The BP predominantly included positive regulation of gene expression, negative regulation of gene expression, negative regulation of transcription by RNA polymerase II, RNA processing, positive regulation of cell population proliferation, negative regulation of apoptotic process, ribosomal RNA processing, RNA catabolic process, heart development, and response to estradiol. The CC terms mainly encompassed nucleolar ribonuclease complex, cytoplasmic ribonuclease complex, nuclear ribonuclease complex, ribonuclease complex, gamma-secretase complex, presynaptic membrane, nucleoplasm, plasma membrane, nucleus, and endosome membrane. The MF terms primarily included 3′-5′ RNA exonuclease activity, protein binding, fibronectin binding, ubiquitin protein ligase binding, integrin binding, tachykinin receptor activity, co-receptor activity, C-X3-C chemokine binding, mRNA 3′-UTR AU-rich region binding, and neuropeptide binding.

**Fig 2:**
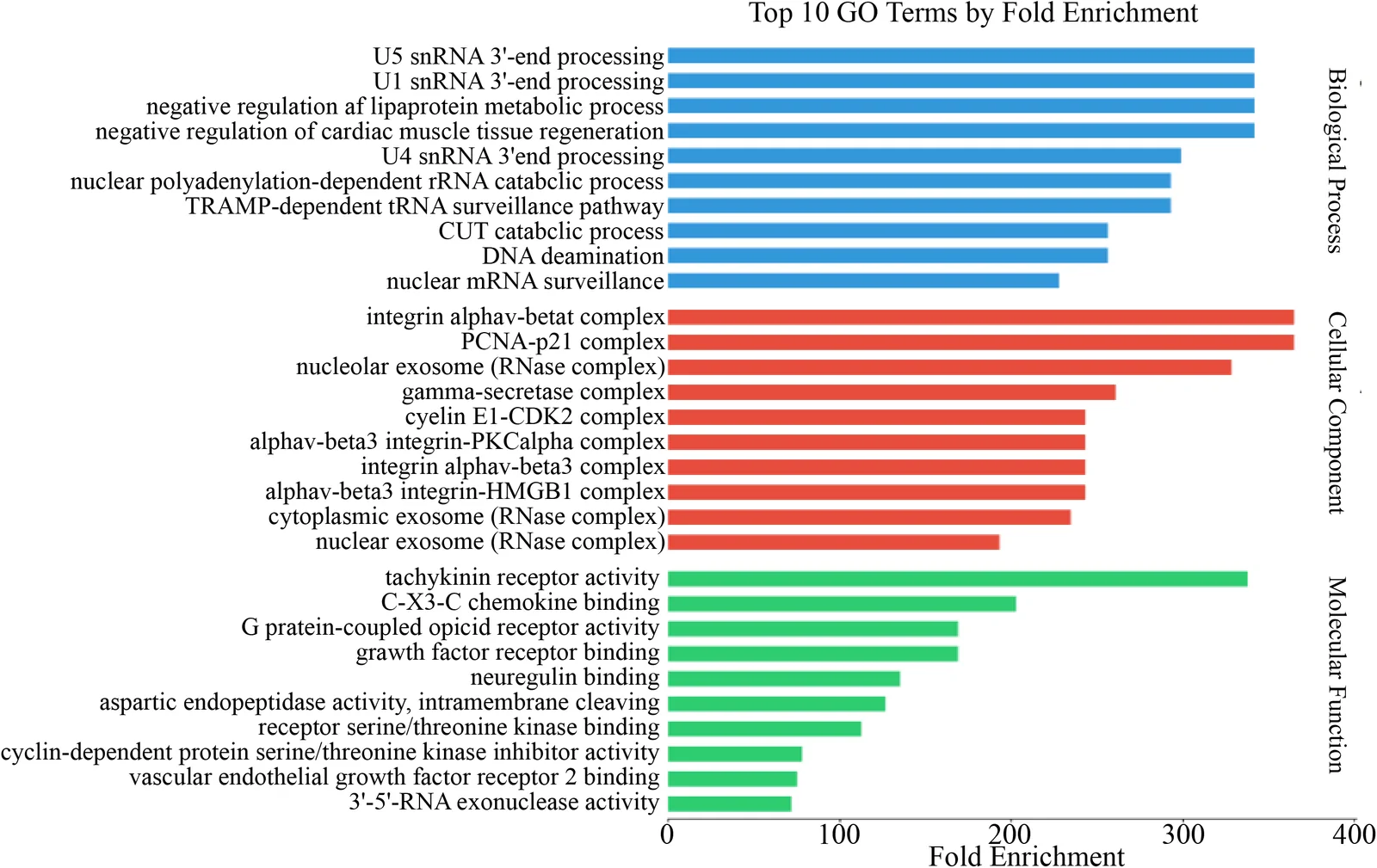
GO Enrichment analysis of potential targets (top 10).

### KEGG pathway enrichment analysis

The 143 target genes were subjected to KEGG pathway enrichment analysis using the DAVID platform, which identified 51 pathways enriched among the overlapping aspartame- and AD-associated targets (Fig 3). The 10 most enriched pathways are presented in Fig 3 and encompass RNA degradation, human papillomavirus infection, pathways in cancer, neuroactive ligand-receptor interaction, small cell lung cancer, fluid shear stress and atherosclerosis, proteoglycans in cancer, PI3K-Akt signaling pathway, the calcium signaling pathway, and Kaposi sarcoma-associated herpesvirus infection. Among these, the pathways most closely linked to AD were RNA degradation, neuroactive ligand-receptor interaction, the PI3K-Akt signaling pathway, and the calcium signaling pathway.

**Fig 3:**
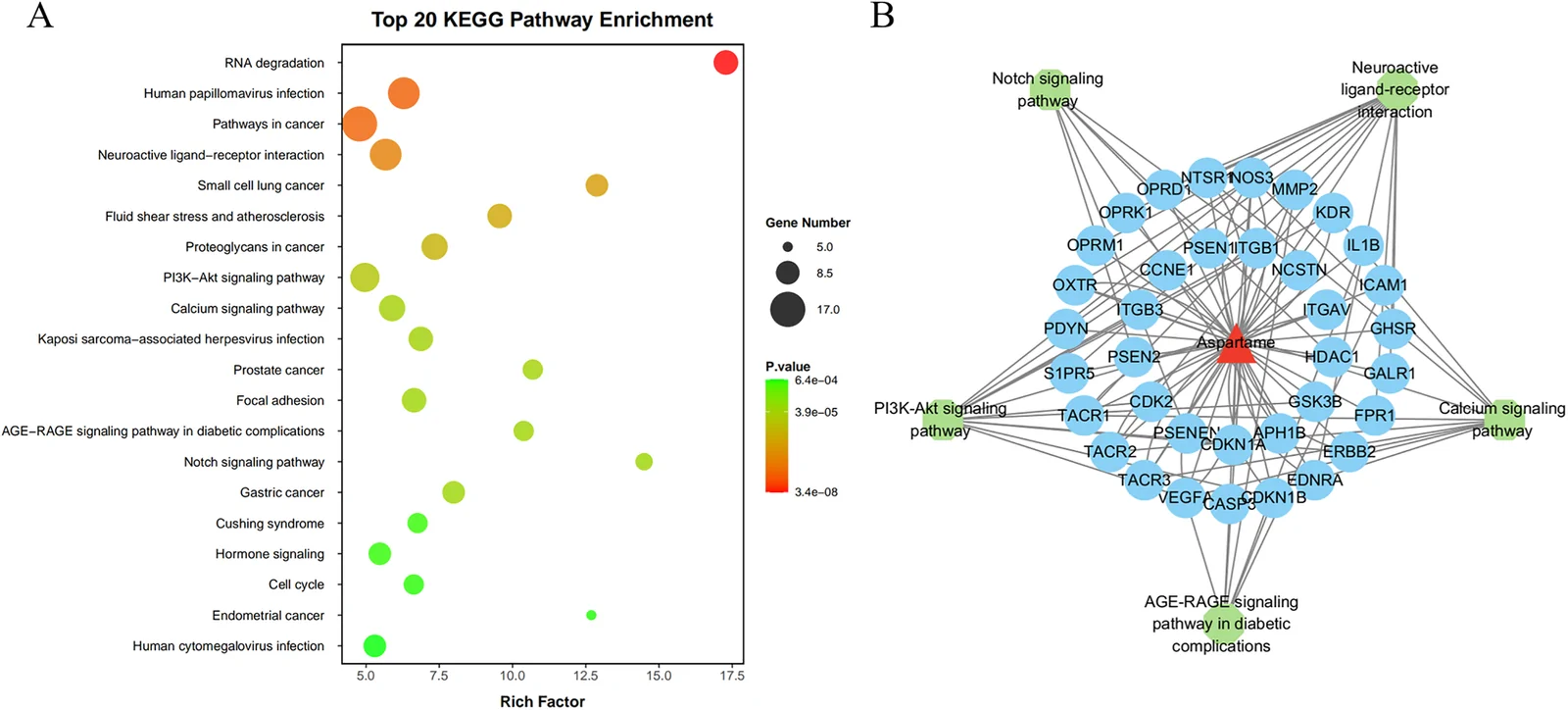
Enrichment analysis diagram. **(A)** KEGG enrichment analysis of potential targets (top 20); **(B)** network map of aspartame-related pathways and targets.

### Gene expression analysis in AD patients and healthy populations

To analyze the expression of the aforementioned signaling pathways and genes in AD patients, we performed single-cell transcriptomic analysis on the prefrontal cortex of 12 AD patients and 9 healthy controls using publicly available data (GSE157827) (Fig 4). Volcano plot results revealed 349 upregulated genes and 107 downregulated genes in AD patients compared to healthy individuals, with PI3K, AKT1, AKT2, AKT3, and iNOS showing statistically significant differential expression.

**Fig 4:**
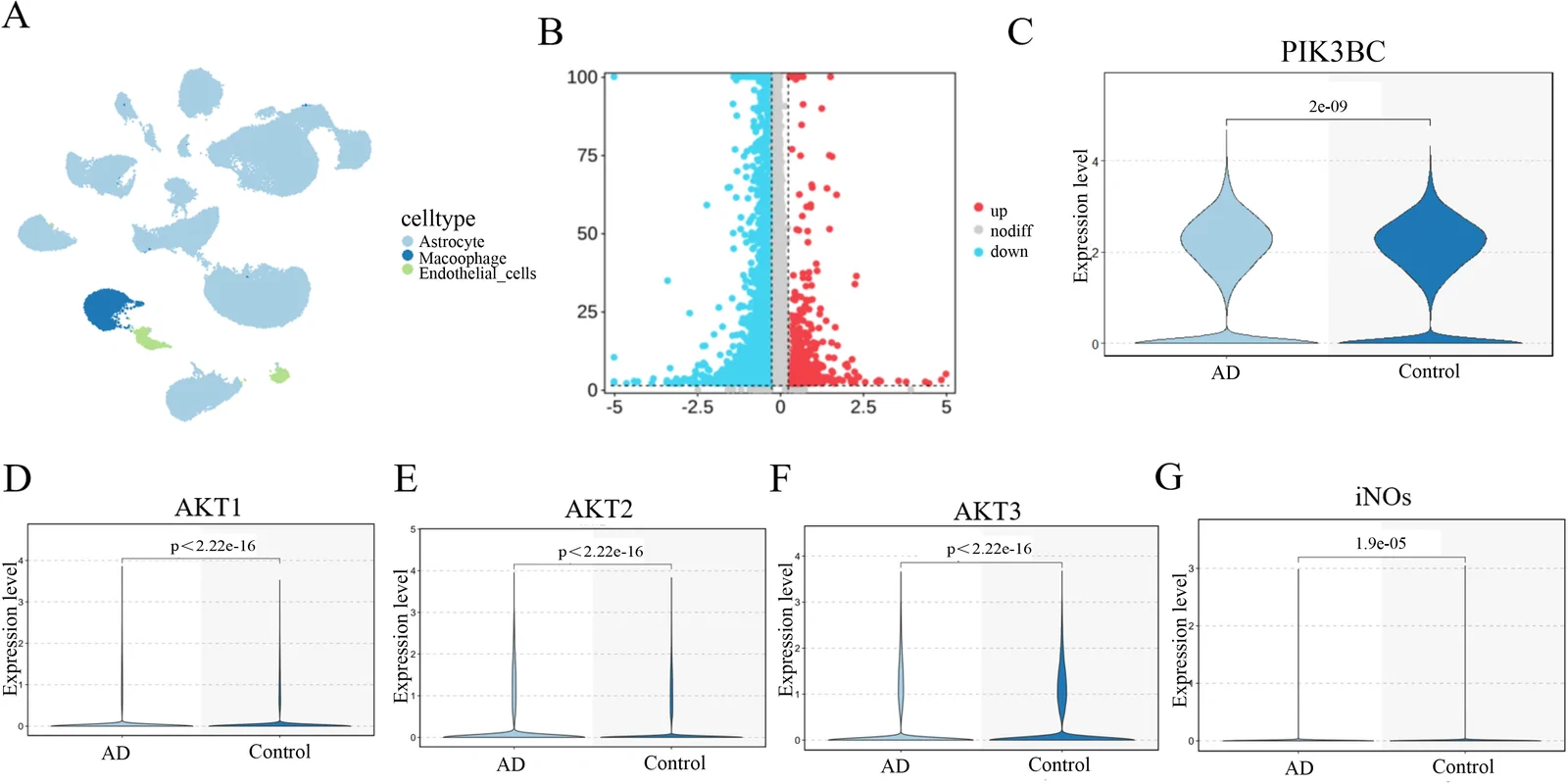
Single-cell RNA sequencing reveals gene expression signatures across specific cell types in the GSE157827 dataset. **(A)** Heatmap showing the average expression levels of differentially expressed genes in major cell types, including astrocytes, macrophages, and endothelial cells. **(B)** Bar plot displaying the number of genes upregulated, unchanged, and downregulated in astrocytes. The expression levels of **(C)** PIK3CB, **(D)** AKT1, **(E)** AKT2, **(F)** AKT3, and **(G)** iNOS.

### Molecular docking results

To evaluate the binding affinity between aspartame and its target proteins, we performed molecular docking using AutoDock Vina (version 1.2.2). The binding conformations and interaction modes of aspartame with the eight target proteins were obtained, and the binding energies of each complex were calculated (Fig 5 and Table 2). Our docking analysis predicted that aspartame may form hydrogen bonds and electrostatic interactions with its protein targets. The predicted binding energies of aspartame with CCNE1 (−4.049 kcal/mol), CDKN1A (−3.837 kcal/mol), CDKN1B (−3.356 kcal/mol), ERBB2 (−3.019 kcal/mol), GSK-3β (−5.002 kcal/mol), KDR (−5.613 kcal/mol), NOS3 (−4.046 kcal/mol), and VEGFA (−2.06 kcal/mol) are computational estimates that indicate varying binding affinities, with the most favorable predicted binding energies observed for GSK-3β and KDR and weaker predicted binding for VEGFA.

**Table 2:** Binding energy for target with aspartame.

Target	PBD Number	Hydrone	Binding energy
CCNE1	8VQ3	Aspartame	−4.049
CDKN1A	8GJF	Aspartame	−3.837
CDKN1B	7OR8	Aspartame	−3.356
ERBB2	1MFG	Aspartame	−3.019
GSK3B	1O6L	Aspartame	−5.002
KDR	4ASE	Aspartame	−5.613
NOS3	4D1P	Aspartame	−4.046
VEGFA	4KZN	Aspartame	−2.06

**Fig 5:**
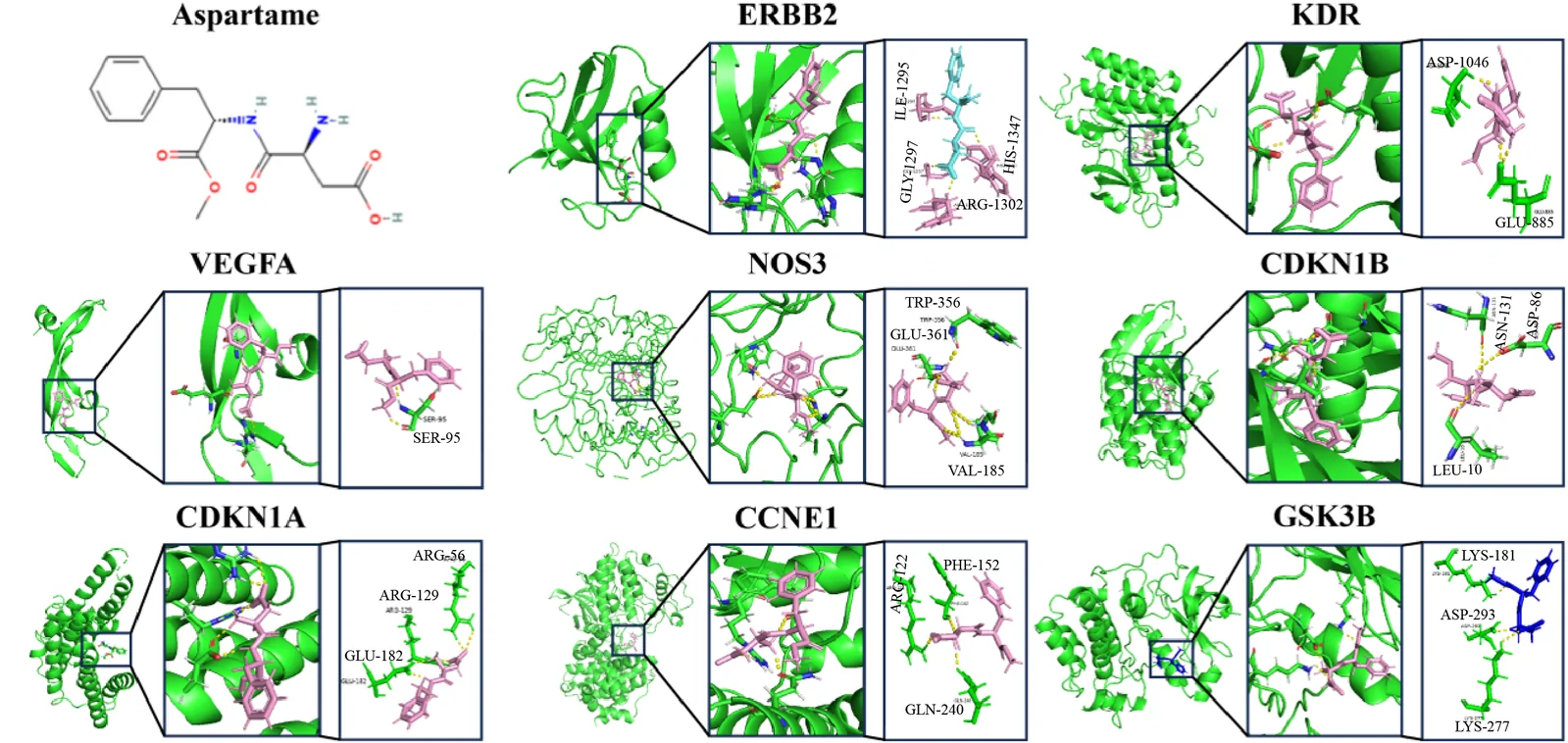
Molecular docking results with the aspartame binding energy for each target.

### Effects of aspartame on BV2 cell viability

We performed a CCK-8 assay to evaluate the effects of aspartame concentrations ranging from 0.05 to 5 mg/mL on BV2 cell viability. Compared with the control group, LPS treatment significantly reduced cell viability to approximately 82% (P < 0.01) (Fig 6). In the aspartame-treated groups, cell viability declined in a marked concentration-dependent manner. Specifically, the 0.05 mg/mL group maintained approximately 97% viability, showing no significant difference from the control and remaining significantly higher than the LPS-treated group. Viability decreased slightly to about 91% at 0.1 mg/mL and continued to fall as the concentration increased: approximately 76% at 0.5 mg/mL, 63% at 1 mg/mL, 51% at 2.5 mg/mL, and only about 32% at 5 mg/mL. Collectively, these findings demonstrate that aspartame exerts a concentration-dependent inhibitory effect on BV2 cell viability.

**Fig 6:**
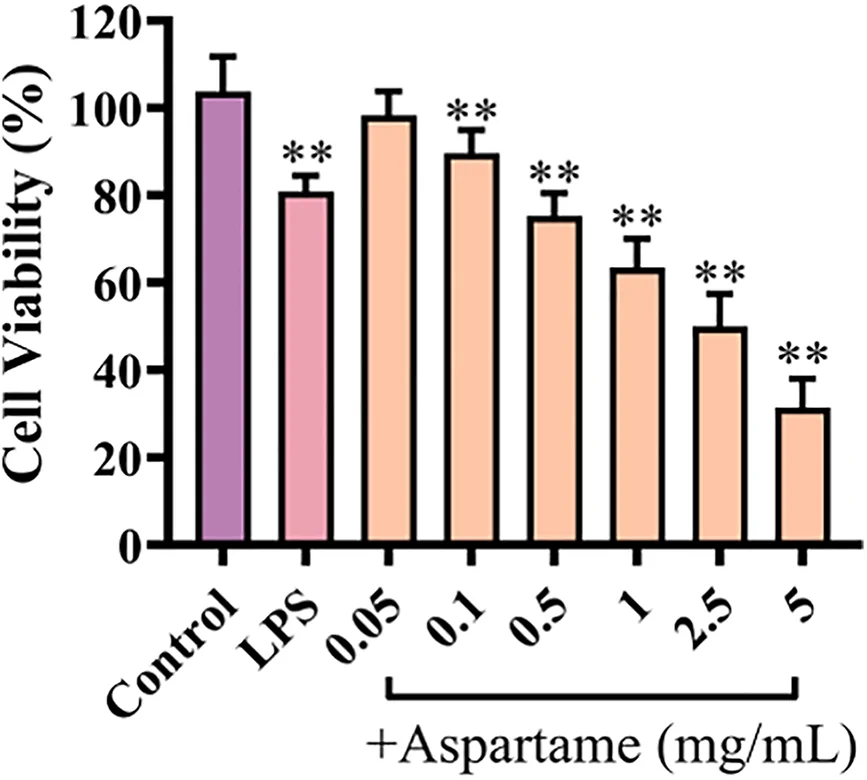
Effect of aspartame on cell viability. Data are presented as mean ± SD from six independent experiments (n = 6 per group). **P < 0.01 compared with the control group, by one-way ANOVA followed by Dunnett’s test.

### Effects of aspartame on NO, TNF-α, IL-1β, and IL-6 release in BV2 cells

To determine whether aspartame triggers nitric oxide (NO) production, we measured NO levels in the culture supernatants of BV2 cells. The NO concentration in the control group was 18.20 ± 1.62 μM, whereas LPS stimulation sharply elevated it to 46.22 ± 2.81 μM. Aspartame treatment induced a concentration-dependent increase in NO release, reaching 19.51 ± 1.23, 23.23 ± 2.29, 28.98 ± 2.50, 29.30 ± 1.96, 30.16 ± 2.01, and 34.69 ± 4.13 μM at 0.05, 0.1, 0.5, 1, 2.5, and 5 mg/mL, respectively (Fig 7A). These findings suggest that aspartame elicits a pro-inflammatory NO response in BV2 microglia, following the same direction as LPS-induced inflammation but at a lower magnitude.

**Fig 7:**
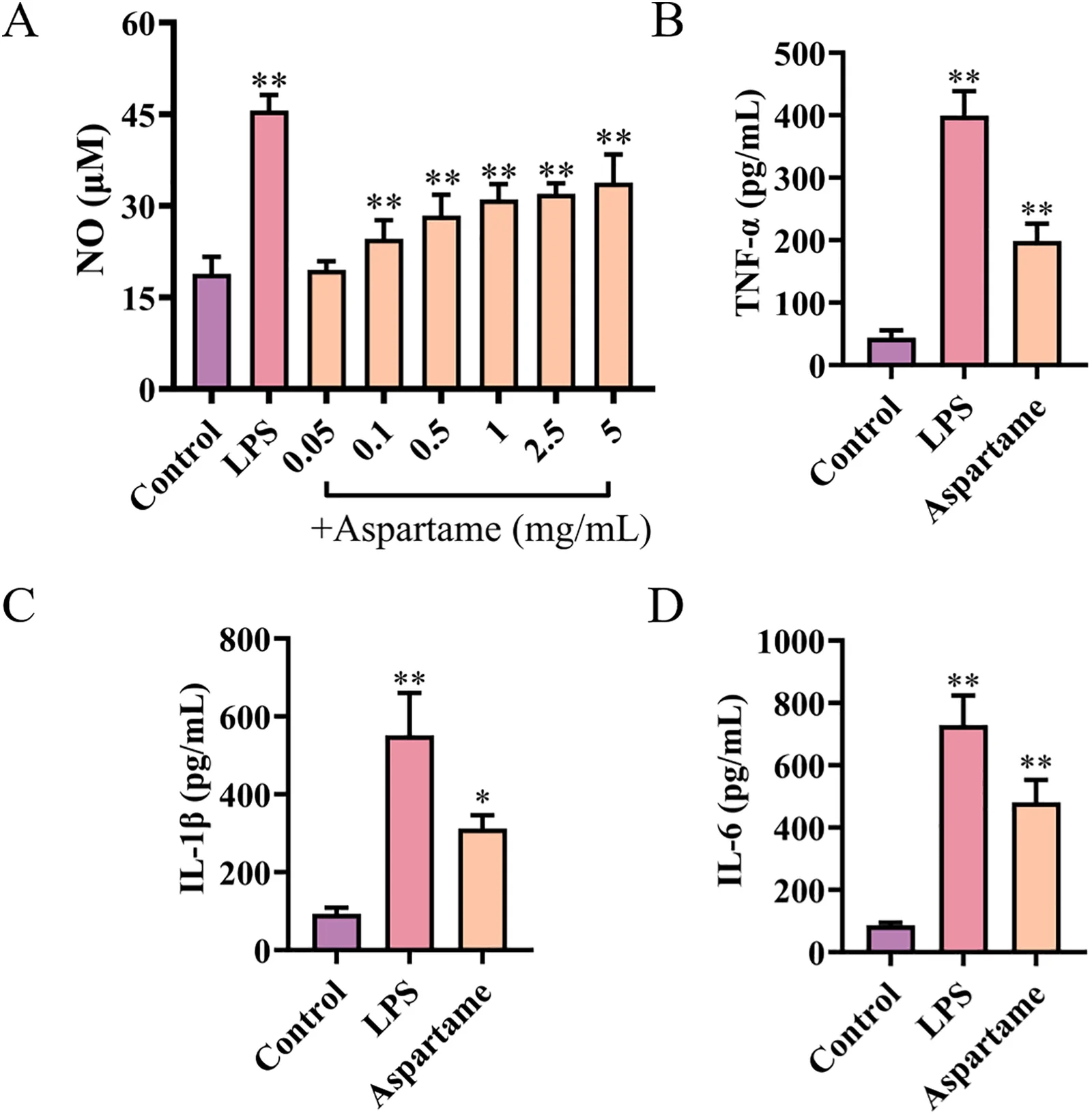
Effects of aspartame stimulation on the production of (A) NO, (B) TNF-α, (C) IL-1β, and (D) IL-6 in BV2 cells. Cell culture supernatants were collected after aspartame treatment and analyzed using an NO detection kit and ELISA kits. Data are presented as mean ± SD from four independent experiments (n = 4). *P < 0.05, **P < 0.01 versus control group, by one-way ANOVA followed by Dunnett’s test.

We next examined the secretion of TNF-α, IL-1β, and IL-6 by ELISA after treatment with 1 mg/mL aspartame. Basal levels of these cytokines in the control group were 44.64 ± 11.31 pg/mL, 86.06 ± 9.19 pg/mL, and 92.88 ± 16.28 pg/mL, respectively. LPS treatment markedly upregulated all three cytokines to 399.57 ± 38.82 pg/mL, 728.99 ± 93.11 pg/mL, and 555.24 ± 105.11 pg/mL. Correspondingly, the aspartame-treated group significantly increased TNF-α, IL-6, and IL-1β levels to 201.61 ± 25.28 pg/mL, 481.33 ± 70.51 pg/mL, and 312.22 ± 36.10 pg/mL (P < 0.01 vs. control) (Fig 7B–7D). Collectively, these results indicate that aspartame promotes a robust inflammatory cytokine response in BV2 microglia, providing in vitro evidence of microglial inflammatory activation.

### Effects of aspartame on TNF-α, IL-1β, IL-6, GSK-3β, iNOS, and IBA-1 mRNA expression in BV2 cells

To investigate the effects of aspartame on the expression of neuroinflammation-related genes in mouse BV2 cells, RT-qPCR was employed to quantify the relative mRNA expression levels of GSK-3β, iNOS, TNF-α, IL-6, IL-1β, and **IBA-1** across all experimental groups (Fig 8). Compared with the control group, aspartame treatment significantly upregulated the mRNA expression of GSK-3β (1.86 ± 0.13), iNOS (2.51 ± 0.15), TNF-α (6.88 ± 1.21), IL-6 (5.13 ± 0.26), IL-1β (5.77 ± 1.41), and **IBA-1 (1.28 ± 0.02)**. LPS treatment markedly elevated the mRNA expression of these six genes, yielding relative levels of 4.27 ± 0.28, 3.06 ± 0.17, 9.00 ± 1.41, 7.63 ± 1.25, 7.16 ± 1.84, and 4.86 ± 0.06, respectively. These findings indicate that aspartame triggered a consistent pro-inflammatory response in BV2 microglia, mirroring the direction of LPS-induced inflammation but at a considerably lower magnitude.

**Fig 8:**
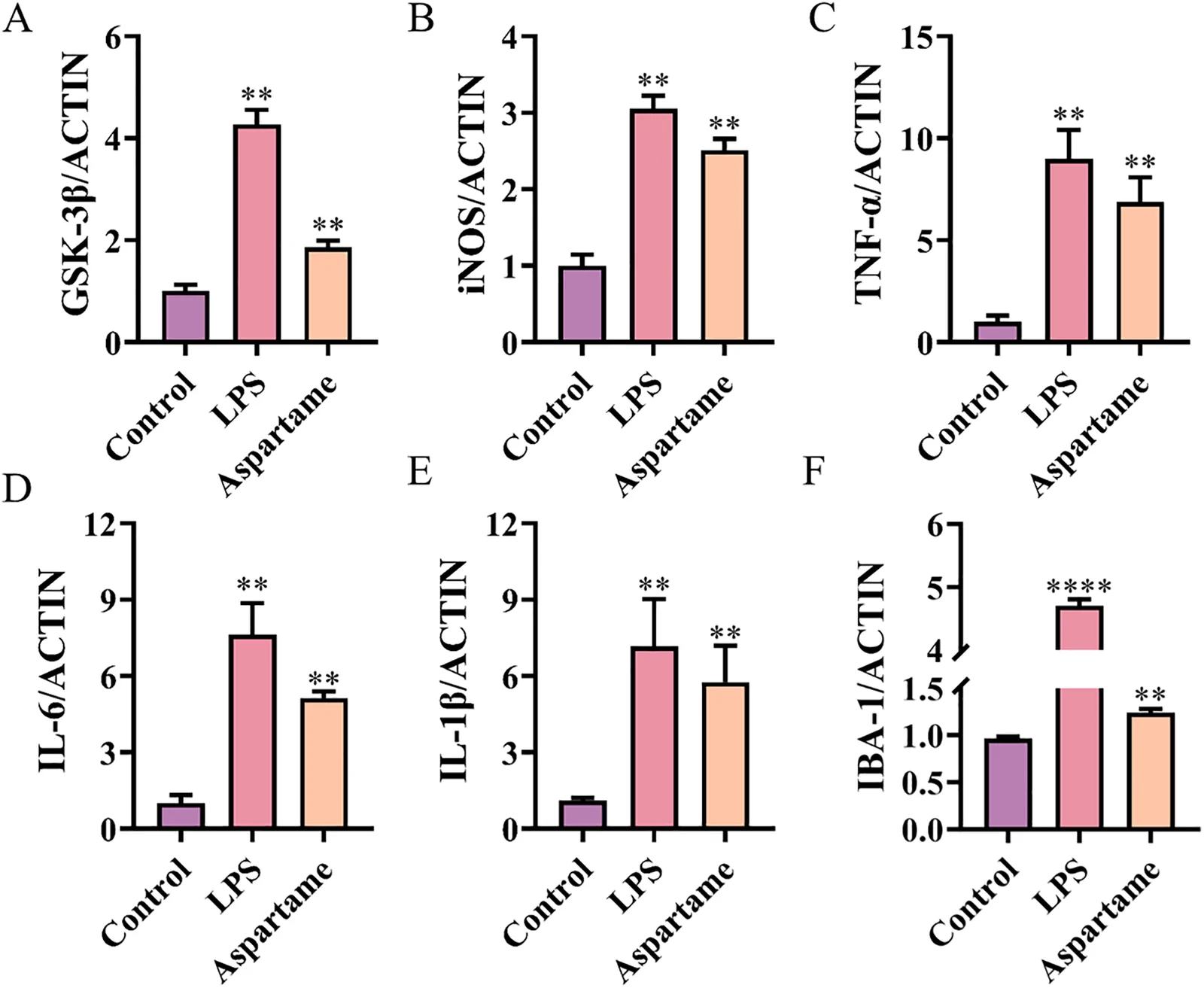
qPCR analysis of mRNA expression levels of (A) GSK-3β, (B) iNOS, (C) TNF-α, (D) IL-6, (E) IL-1β, (F) IBA-1. Data are presented as mean ± SD from three independent experiments (n = 3). **P < 0.01, ****P < 0.0001 versus the control group, by one-way ANOVA followed by Dunnett’s test.

## Discussion

This study employed network toxicology, molecular docking, and in vitro experiments to elucidate the potential neurotoxic effects of the artificial sweetener aspartame in AD and to characterize its associated molecular network.

Our network toxicology analysis identified 185 common targets linking aspartame and AD (Fig 1). The greater number of targets identified here, compared with prior aspartame-related studies in gastric cancer (85 targets) [22] and metabolic associated fatty liver disease (105 targets) [23], indicates more extensive connectivity within the AD-associated molecular network and suggests that aspartame may modulate neurological function through multiple signaling pathways [24]. These findings provide a new perspective and generate mechanistic hypotheses regarding the potential involvement of aspartame in AD-related pathways.

Our work serves as a significant network-level complement to the existing research. Griebsch et al. demonstrated that treating human SH-SY5Y neuroblastoma cells with aspartame and its three metabolites (aspartic acid, phenylalanine, and methanol) significantly induced oxidative stress and mitochondrial damage, and disrupted neuronal lipid homeostasis [10]. However, they did not systematically analyze the upstream signaling pathway perturbations at a network level. In this study, KEGG enrichment analysis revealed that the potential targets of aspartame are significantly enriched in the PI3K/Akt signaling pathway, calcium signaling pathway, and neuroactive ligand-receptor interaction pathway [9]. Further analysis of single-cell transcriptomic data showed that in the prefrontal cortex of AD patients, the expression of PIK3CB is significantly downregulated, whereas the transcript levels of AKT family members (AKT1, AKT2, AKT3) exhibit an upward trend. This observation may reflect a compensatory cellular feedback mechanism, in which transcriptional upregulation of AKT isoforms attempts to offset reduced kinase activity [25]. In 2025, Rekha et al. systematically pointed out that deficits in the PI3K/Akt signaling pathway are a key mechanism leading to the disinhibition of GSK-3β, thereby promoting Tau protein hyperphosphorylation [26]. Recent studies have also confirmed that pharmacological modulation of the PI3K/Akt/GSK-3β axis can ameliorate brain insulin resistance and mitigate Tau pathology and Aβ accumulation [27,28]. Notably, the binding energy between aspartame and GSK-3β was −5.002 kcal/mol (Fig 5), and aspartame significantly upregulated GSK-3β mRNA expression in BV2 microglial cells. We therefore propose that aspartame binds to PI3K subunits (e.g., PIK3CB), directly inhibiting pathway activity. This results in diminished Akt activity, leading to reduced inhibitory Ser9 phosphorylation of GSK-3β, while simultaneously increasing its expression at the transcriptional level [29]. This hypothesis requires further validation in subsequent studies using methods such as immunoblotting.

In the PPI network analysis, KDR, CCNE1, and GSK-3β emerged as core hub proteins with high connectivity, suggesting their potential involvement in aspartame-associated networks relevant to AD pathology. Our experimental focus was deliberately placed on GSK-3β. This prioritization was guided by the well-established role of GSK-3β as a central kinase driving microglial neuroinflammation, directly regulating the production of pro-inflammatory cytokines, iNOS, and nitric oxide [30,31]. In contrast, KDR (VEGFR2) functions primarily in endothelial angiogenesis and vascular permeability, with limited known involvement in canonical microglial inflammatory cascades [32], and CCNE1 (Cyclin E1) is predominantly characterized as a neuronal cell cycle regulator linked to neurodegeneration rather than microglial activation [33]. The differential pathway enrichment patterns-GSK-3β in neuroinflammation-related signaling versus KDR and CCNE1 in angiogenesis and cell cycle pathways—further support this selective approach. Nevertheless, the potential contributions of KDR and CCNE1 to aspartame-induced neurotoxicity cannot be discounted and may involve alternative pathological dimensions that are beyond the scope of the present microglial model. The present study was designed as an initial investigation for the neuroinflammatory hypothesis; therefore, comprehensive multi-target validation, including the functional characterization of KDR and CCNE1 in appropriate co-culture or in vivo models, is warranted in future work to fully delineate the neurotoxicological profile of aspartame.

**Scheme 1:**
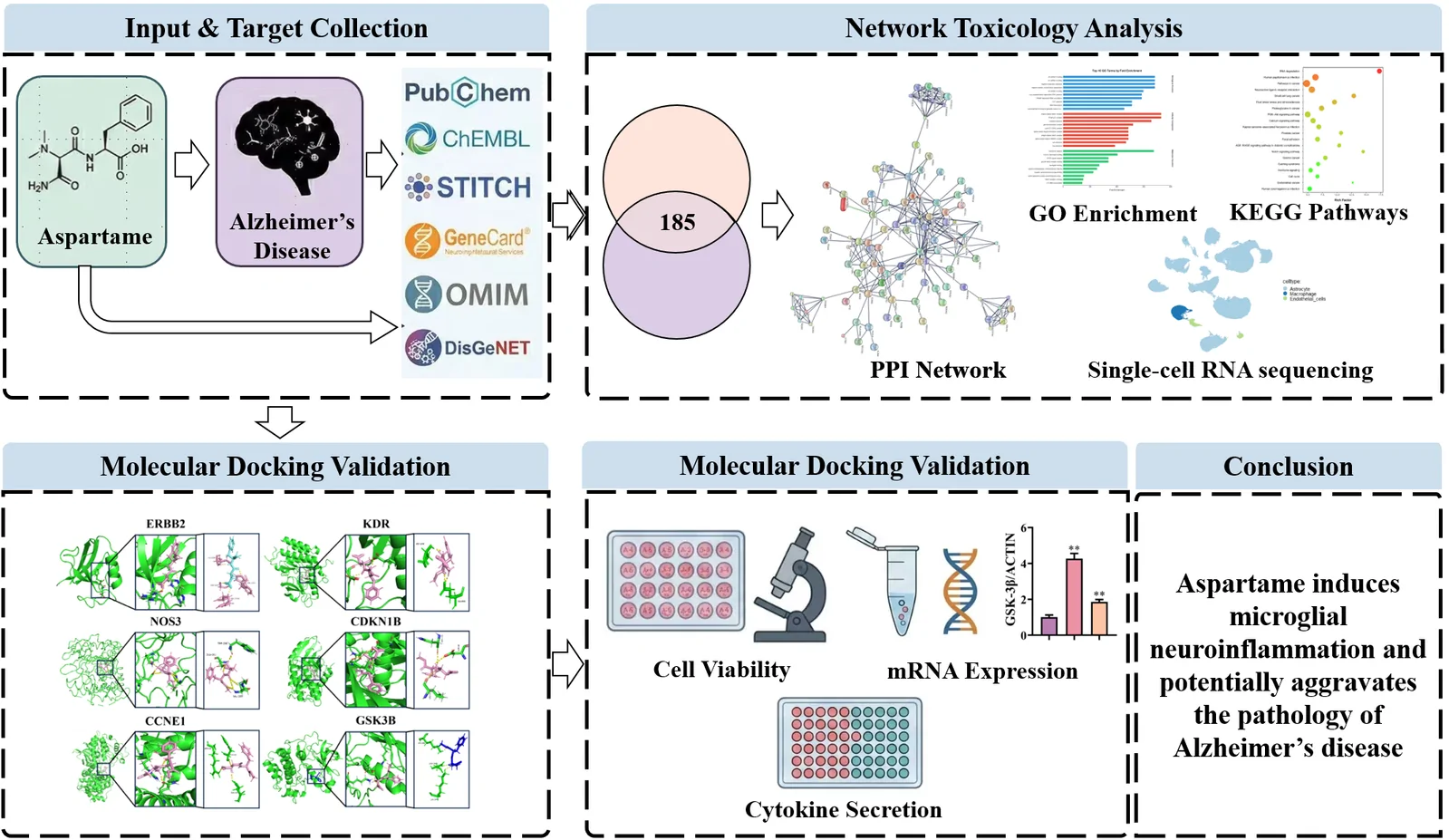
Schematic diagram of the integrated network toxicology and experimental validation strategy to investigate the neuroinflammatory potential of aspartame in Alzheimer’s disease (AD).

It is noteworthy that through network toxicology analysis, we found that aspartame’s potential neurotoxic effects are related to disturbances in RNA metabolic homeostasis and the γ-secretase complex. Among the KEGG enrichment results, the RNA degradation pathway had the highest enrichment, 3′-5′ RNA exonuclease activity ranked first in the GO molecular function enrichment, and the γ-secretase complex was significantly enriched in the cellular component category [34].

Recent studies have drawn attention to the role of aberrant RNA metabolism in the pathogenesis of AD. In 2007, Nunomura et al. proposed that RNA, due to its single-stranded structure and lack of histone protection, is more sensitive to oxidative stress than DNA, with RNA oxidative damage appearing in susceptible neurons in the early stages of AD [35]. In 2023, Castro-Hernández et al. found that the m6A RNA methylation modification level was significantly reduced in the brains of AD patients and aging animals, leading to impaired synthesis of synaptic function-related proteins like CAMKII and GLUA1 [36]. The RNA modification-translation regulation axis directly links RNA metabolism abnormalities to synaptic dysfunction in AD [37].

In this study, we not only observed the overall enrichment of the RNA degradation pathway, but also found a systematic enrichment of nucleic acid complexes (nucleolar, cytoplasmic, and nuclear nucleic acid complexes) in the GO cellular component analysis. These results suggest that aspartame may not only act on a single RNA metabolism node but may broadly interfere with multiple processes from RNA processing and modification to degradation [38]. Interestingly, the simultaneous enrichment of the γ-secretase complex suggests that aspartame may be computationally linked to both Aβ generation and RNA metabolite processing [39,40]; however, these predictions require experimental validation. This finding opens up a new direction for studying the neurotoxic mechanisms of aspartame.

Our in vitro experiments showed that aspartame inhibited BV2 cell viability in a concentration-dependent manner within the range of 0.5–5 mg/mL and significantly induced NO release as well as secretion of TNF-α, IL-1β, and IL-6. The mRNA expression levels of iNOS, TNF-α, IL-1β, IL-6, and GSK-3β were significantly upregulated, closely resembling the pattern observed in the LPS treatment group.

These results suggest that, in this in vitro model, aspartame may drive microglial polarization toward a pro-inflammatory M1-like phenotype, potentially involving methanol metabolites and aspartic acid-mediated excitotoxicity. However, whether these mechanisms lead to chronic, sustained release of pro-inflammatory factors or degenerative changes in neurons and glial cells cannot be concluded from the present study [41–43]. While aspartame induced dose-dependent NO production across 0.05–5 mg/mL in BV2 microglia, detailed mechanistic validation of GSK-3β mRNA and TNF-α, IL-1β, and IL-6 mRNA levels was performed at 1 mg/mL. This concentration maintained cell viability at approximately 63% (Fig 6), which represents a substantial reduction. Therefore, although this dose allowed us to examine inflammatory activation, we cannot exclude the possibility that cellular stress or cytotoxicity contributed to the observed increases in inflammatory markers. Accordingly, 1 mg/mL should not be considered a sub-cytotoxic concentration, and the mechanistic findings at this dose should be interpreted with this caveat. This targeted focus allowed us to explore a possible involvement of the NO/oxidative stress-GSK-3β/NF-κB cytokine signaling axis.

Prior studies have reported NO induction by aspartame in a dose-dependent manner and have linked these mediators to microglial activation in models of cognitive impairment and AD [44]. For instance, aspartame consumption was shown to shift microglia toward an activated phenotype via methanol-induced toxicity and aspartic acid-mediated excitotoxicity, resulting in sustained release of pro-inflammatory cytokines that contribute to cognitive decline [9]. Similarly, aspartame exacerbates cerebral ischemia–reperfusion injury through upregulation of TNF-α, IL-1β, and IL-6 mRNA alongside increased NF-κB activity [45]. These findings align with our observations and support the pro-inflammatory potential of aspartame in in vitro microglial models, although the concentrations used here are much higher than those expected from normal dietary exposure.

A recent study by Ge et al. (2026) also applied network toxicology and molecular docking to investigate the potential links between aspartame and Alzheimer’s disease [15]. That work provided a valuable computational prediction of targets and pathways. The present study differs in several important respects. First, we combined computational analysis with in vitro experimental validation, using BV2 microglial cells to demonstrate that aspartame triggers a dose-dependent pro-inflammatory response. Second, we focused specifically on microglial neuroinflammation and selected GSK-3β from the hub genes for further investigation, showing that aspartame upregulates GSK-3β mRNA and increases the secretion and mRNA expression of cytokines (TNF-α, IL-1β, and IL-6). However, these data do not establish that GSK-3β mediates these changes. Third, we compared the aspartame-induced response with the classical LPS-induced inflammatory profile, the resemblance in inflammatory readouts suggests a similar direction of response, but it does not demonstrate that the two treatments act through the same mechanism. The two studies are complementary: while Ge et al. established the computational groundwork, our experimental data provide initial in vitro evidence that aspartame can induce a pro-inflammatory response in BV2 microglia, with GSK-3β mRNA upregulation as a possible associated feature. The involvement of GSK-3β in mediating this response remains to be tested.

It is important to note that the observed cellular responses may be influenced not only by biochemical pathway activation, but also by the physicochemical properties of aspartame. Previous studies have reported that aspartame molecules can spontaneously assemble under physiological conditions to form amyloid-like nanofiber structures with regular β-sheet conformations [46]. These physical structures can induce cross-seeding effects on Aβ peptides, globular proteins, and aromatic metabolites, converting their native conformations into β-sheet-rich abnormal fibers, while directly causing red blood cell hemolysis, DNA damage, and cell apoptosis and necrosis. This study did not assess or rule out the self-assembly behavior of aspartame under culture conditions, and this potential confounding factor may influence the mechanistic interpretation of the in vitro results, which requires further investigation and control in future studies.

Although this study has revealed the potential neurotoxic effects of aspartame through network toxicology, bioinformatics analysis, molecular docking, and experimental validation, there are still some limitations. For example, there is a lack of direct evidence of post-translational modifications of proteins, and the effects of aspartame exposure on the phosphorylation states of key sites like Akt and GSK-3β were not assessed using immunoprecipitation-immunoblotting techniques [47]. The BV2 immortalized microglial cell line, although widely used for neuroinflammation screening, differs in its characteristics from primary and human microglia. A comparative review has shown that BV2 cells diverge from primary microglia in proliferation rate, migration capacity, and the magnitude of cytokine secretion upon stimulation [48, 49]. Their inflammatory responses can be either exaggerated or blunted. Therefore, the quantitative aspects of cytokine release measured here may not directly mirror the behavior of native brain microglia. An additional difference concerns transcriptomic identity: extended in vitro culture and immortalization are known to remodel microglial gene expression [50]. Cultured cell lines tend to lose the unique homeostatic gene signature that characterizes true microglia within the brain environment, meaning that the molecular context of our observations does not fully replicate the in vivo state. A further consideration is species origin. Recent transcriptomic studies have established that human microglia possess species-specific regulatory elements and are enriched for Alzheimer’s disease risk variants [51]. As a murine cell line, BV2 cells cannot capture these human-specific biological features.

The GSK-3β mRNA upregulation and pro-inflammatory response we observed therefore provide preliminary evidence of an inflammatory response accompanied by increased GSK-3β mRNA expression rather than a demonstrated mechanistic pathway; however, they do not constitute a quantitative prediction of the human microglial response. Moreover, the 1 mg/mL concentration used for mechanistic experiments reduced BV2 viability to approximately 63%, so cellular stress or cytotoxicity may have contributed to the inflammatory readouts. This concentration should therefore not be termed sub-cytotoxic, and future work should test lower, non-cytotoxic concentrations with appropriate controls. Confirmation in human iPSC-derived microglia, primary human cells, or brain organoid models will also be essential to assess the translational significance of this pathway. Additionally, our findings suggest that aspartame may interact with multiple pathways relevant to central nervous system function, including the PI3K-Akt signaling cascade, RNA metabolism, microglial-mediated neuroinflammation, and protein homeostasis [17,52,53]. These hypotheses are based on network predictions and high-concentration in vitro experiments, and they do not demonstrate that normal dietary consumption of aspartame causes RNA metabolic disturbances, protein homeostasis imbalance, or cellular stress in the central nervous system. Rather, our study highlights the need for further investigation into the neurological safety of food additives using physiologically relevant exposure models and in vivo validation.

## Conclusion

In summary, this study integrated network toxicology, molecular docking, and in vitro experiments to explore the potential neurotoxic mechanisms of the artificial sweetener aspartame in AD. Through network toxicology analysis, we identified 185 overlapping targets between aspartame and AD. Functional enrichment analysis revealed that these targets are significantly enriched in pathways related to RNA degradation, PI3K-Akt signaling, and calcium signaling. Molecular docking revealed varying binding affinities between aspartame and these core proteins, with GSK-3β and KDR showing the strongest interactions. In vitro experiments further demonstrated that aspartame concentration-dependently increased NO release, upregulated the secretion of pro-inflammatory cytokines (TNF-α, IL-1β, and IL-6), and elevated the mRNA expression of inflammation-related genes such as GSK-3β and iNOS in BV2 microglial cells. These findings suggest that aspartame may induce a pro-inflammatory response in BV2 microglial cells, accompanied by increased GSK-3β mRNA expression. This study provides a basis for further investigation into the neurological safety of aspartame and highlights the need for future studies using physiologically relevant exposure models.
