# Supplementary material for: Unveiling the neuroinflammatory potential of aspartame in Alzheimer’s disease: A comprehensive network toxicology and experimental validation approach
Source: PLoS One. 2026 Nov 3;21(11):e0361766. doi: 10.1371/journal.pone.0361766 (PMC13730326; doi:10.1371/journal.pone.0361766)
Supplement: Supplementary file 2 [file pone.0361766.s003.docx]

PONE-D-26-23444R1

Unveiling the Neuroinflammatory Potential of Aspartame in Alzheimer's Disease: A Comprehensive Network Toxicology and Experimental Validation Approach

PLOS One
Dear Dr. Sheng,
Thank you for submitting your manuscript to PLOS One. After careful consideration, we feel that it has merit but does not fully meet PLOS One’s publication criteria as it currently stands. Therefore, we invite you to submit a revised version of the manuscript that addresses the points raised during the review process.
Please submit your revised manuscript by Oct 14 2026 11:59PM. If you will need more time than this to complete your revisions, please reply to this message or contact the journal office at [plosone@plos.org](mailto:plosone@plos.org). When you're ready to submit your revision, log on to https://www.editorialmanager.com/pone/ and select the 'Submissions Needing Revision' folder to locate your manuscript file.
Please include the following items when submitting your revised manuscript:

- A letter that Responses to each point raised by the academic editor and reviewer(s). You should upload this letter as a separate file labeled 'Response to Reviewers'.
- A marked-up copy of your manuscript that highlights changes made to the original version. You should upload this as a separate file labeled 'Revised Manuscript with Track Changes'.
- An unmarked version of your revised paper without tracked changes. You should upload this as a separate file labeled 'Manuscript'.

If you would like to make changes to your financial disclosure, please include your updated statement in your cover letter. Guidelines for resubmitting your figure files are available below the reviewer comments at the end of this letter.
If applicable, we recommend that you deposit your laboratory protocols in protocols.io to enhance the reproducibility of your results. Protocols.io assigns your protocol its own identifier (DOI) so that it can be cited independently in the future. For instructions see: <https://journals.plos.org/plosone/s/submission-guidelines#loc-laboratory-protocols>. Additionally, PLOS One offers an option for publishing peer-reviewed Lab Protocol articles, which describe protocols hosted on protocols.io. Read more information on sharing protocols at <https://plos.org/protocols?utm_medium=editorial-email&utm_source=authorletters&utm_campaign=protocols>.

As the corResponseing author, your ORCID iD is verified in the submission system and will appear in the published article. PLOS supports the use of ORCID, and we encourage all coauthors to register for an ORCID iD and use it as well. Please encourage your coauthors to verify their ORCID iD within the submission system before final acceptance, as unverified ORCID iDs will not appear in the published article. *Only* the individual author can complete the verification step; PLOS staff *cannot* verify ORCID iDs on behalf of authors.
We look forward to receiving your revised manuscript.

Kind regards,

António Raposo
Academic Editor
PLOS One

Response: Thank you very much for your email and for giving us the opportunity to revise our manuscript. We are truly grateful for your careful consideration and for the constructive comments provided by you and the reviewer. These comments have been extremely helpful in improving the clarity, caution, and overall quality of our work. We have carefully revised the manuscript in response to all the comments. A detailed point-by-point response to each comment is provided in the separate “Response to Reviewers” file. We have also prepared a marked-up copy of the manuscript with track changes and an unmarked version, as requested.

Journal Requirements:

1. If the reviewer comments include a recommendation to cite specific previously published works, please review and evaluate these publications to determine whether they are relevant and should be cited. There is no requirement to cite these works unless the editor has indicated otherwise.

Response: We have carefully reviewed the reviewer comments for this round and note that the reviewer did not recommend any specific previously published works for citation. We have therefore not added any references on this basis. We sincerely appreciate your reminder, which helps ensure that our reference list remains appropriate and fully compliant with the journal’s policy.

2. Please review your reference list to ensure that it is complete and correct. If you have cited papers that have been retracted, please include the rationale for doing so in the manuscript text, or remove these references and replace them with relevant current references. Any changes to the reference list should be mentioned in the rebuttal letter that accompanies your revised manuscript. If you need to cite a retracted article, indicate the article’s retracted status in the References list and also include a citation and full reference for the retraction notice.
Response: We have thoroughly checked the reference list for completeness and accuracy. We confirm that no retracted articles are cited in our manuscript.

**Additional Editor Comments:**
(1) The revised manuscript is clearly improved, and the authors have addressed most of the concerns raised in the previous round. The additional IBA-1 data, clarification of the aspartame concentration used in the qPCR and ELISA experiments, revision of the molecular docking interpretation, and the expanded discussion of the limitations of the BV2 model have all strengthened the paper. I still think that a few points need to be corrected before the manuscript can be accepted.

Response: We sincerely thank the Editor for the careful review and for the positive assessment of our revised manuscript. We agree that a few points still require correction before the manuscript can be accepted, and we will address them carefully in the next revision.

(2) The main issue remains the interpretation of the aspartame concentrations used in vitro. The authors now acknowledge that the tested concentrations are much higher than those expected after normal dietary exposure, which is important. However, some parts of the Discussion still go too far in linking these findings to possible human neurological risk. The arguments concerning chronic exposure, blood-brain barrier dysfunction, metabolite accumulation, or higher local brain exposure are interesting possibilities, but they are not demonstrated by the present study. I would therefore suggest keeping the interpretation more firmly at the level of a mechanistic in vitro model and avoiding implications regarding usual human consumption.

Response: We sincerely thank the Editor for this insightful comment. The in vitro concentrations should remain firmly at the level of a mechanistic cell model and should not be extended to imply risks from usual human consumption. In the revised Discussion, we have carefully moderated all statements that could be read as linking our findings to human neurological risk. Specifically, we have replaced phrases such as “experimental evidence for the involvement of aspartame in the pathogenesis of AD” with “generate mechanistic hypotheses regarding the potential involvement of aspartame in AD-related pathways, although further validation is required.” We have also removed the claim that our findings underscore the pro-inflammatory potential of aspartame at “pathophysiologically relevant exposures” and instead state that the concentrations used are much higher than those expected from normal dietary exposure. The statement that aspartame may “synergistically accelerate the pathological progression of AD” has been revised to emphasize that this is a computational prediction requiring experimental validation and should not be interpreted as direct evidence of accelerated AD pathology. Furthermore, we have deleted the sentence implying that long-term aspartame consumption may provoke RNA metabolic disturbances, protein homeostasis imbalance, and cellular stress in the central nervous system, and replaced it with a clear statement that our hypotheses are based on network predictions and high-concentration in vitro experiments and do not demonstrate such effects from normal dietary consumption. We have also added a caveat that the 1 mg/mL concentration reduced cell viability to approximately 63%, so we cannot exclude the possibility that cellular stress or cytotoxicity contributed to the inflammatory responses. Throughout the revised Discussion, we now consistently describe the work as providing initial in vitro evidence in a BV2 cell model and call for future validation using physiologically relevant exposure models and in vivo systems.

We believe these changes fully address the Editor’s concern while preserving the scientific value of our study. The revision is highlighted in red from lines 10-11 on page 7, lines 27-30 on page 19, lines 1on page 20, lines 27-30 on page 21, lines 1-2 on page 22.
(3) I also remain concerned about the use of 1 mg/mL aspartame for the mechanistic experiments. At this concentration, cell viability was approximately 63%. This is a substantial reduction in viability, and it is difficult to exclude the possibility that cellular stress or cytotoxicity contributed to the increase in inflammatory markers. For this reason, I would avoid describing 1 mg/mL as a “sub-cytotoxic” concentration and would add this point explicitly to the limitations.

Response: We sincerely thank the Editor for this important and constructive comment. 1 mg/mL aspartame used for the mechanistic experiments cannot be regarded as sub-cytotoxic, because cell viability was reduced to approximately 63%. We also agree that, at this level of viability reduction, it is difficult to exclude the possibility that cellular stress or cytotoxicity contributed to the increases in inflammatory markers. In the revised manuscript, we have therefore removed the phrase “without excessive cytotoxicity” from the Discussion and replaced it with a clear statement that this concentration reduced cell viability to approximately 63%, that cellular stress or cytotoxicity cannot be excluded as a contributing factor, and that the mechanistic findings at this concentration should be interpreted with this caveat. In addition, we have explicitly added this point to the Limitations section. The revised text now states that 1 mg/mL should not be described as a sub-cytotoxic concentration, that the inflammatory responses observed at this dose may partly reflect a stress response, and that future studies should include a wider range of lower, non-cytotoxic concentrations together with rigorous cytotoxicity controls to better distinguish direct pro-inflammatory effects from secondary responses to cellular damage.

We believe these changes directly address the Editor’s concern and ensure that our conclusions remain appropriately cautious and transparent. The revision is highlighted in red from lines 27-30 on page 21, lines 1-2 on page 22.
(4)The statistical section has improved, particularly with the clarification that Dunnett’s test was used. However, I do not think the statement that the qPCR experiments were “sufficiently powered” is justified when only three biological replicates were used and no formal power analysis was performed. This sentence should be removed or softened, and the exploratory nature of these data should be stated more clearly.

Response: We sincerely thank the Editor for this careful statistical comment. In the revised Statistical Analysis section, we now explicitly state that the qPCR data should be regarded as exploratory and hypothesis-generating rather than confirmatory. These changes make the exploratory nature of the qPCR data clear and ensure that our statistical reporting is appropriately cautious and consistent throughout the manuscript. We are grateful to the Editor for pointing this out. The revision is highlighted in red from lines 6-9 on page 8.
(5) Some of the wording in the Results and Discussion should also be moderated. For example, statements suggesting that the experiments provide “direct experimental evidence” for a role of aspartame in Alzheimer’s disease-associated neuroinflammation are stronger than the model can support. The data show an inflammatory response in an immortalized murine microglial cell line under relatively high in vitro concentrations of aspartame, but they do not directly demonstrate an effect on Alzheimer’s disease pathogenesis.

Response: We sincerely thank you for this careful comment. Our data show an inflammatory response in an immortalized murine microglial cell line under relatively high in vitro concentrations of aspartame, but they do not directly demonstrate an effect on Alzheimer’s disease pathogenesis. We have therefore moderated the relevant wording in the Results and Discussion sections. In the Results, we removed the phrase “direct experimental evidence” and revised the sentence to state that the findings provide “in vitro evidence of microglial inflammatory activation.” In the Discussion, we also softened the description of our experimental data as “the first biological evidence” and now describe them as “initial in vitro evidence” in a BV2 cell model. These changes keep our interpretation firmly at the level of a mechanistic in vitro model and avoid overstating the translational implications of the study. We are grateful to the Editor for this helpful guidance. The revision is highlighted in red from lines 3 on page 15.
(6) I also noticed an inconsistency in the revision documents. The cover letter states that corrections for multiple comparisons were included, whereas the response to the statistical comment states that no formal multiplicity correction was applied across endpoints and that the reported p-values are nominal. This should be made consistent before resubmission.

Response: We sincerely thank you for identifying this inconsistency between the revision documents. We fully agree that the cover letter and the response to the statistical comment should not contradict each other, and we apologize for the confusion caused by the wording in the cover letter.

To clarify the manuscript does not include formal corrections for multiple comparisons. The p-values reported in our study are nominal and were not adjusted for multiplicity across endpoints. The statement in the cover letter referring to “corrections for multiple comparisons” was inaccurate and did not reflect the actual statistical approach used in the study. To ensure full consistency between the manuscript and our response, we have revised the Statistical Analysis section to state explicitly: “No formal correction for multiple comparisons was applied.” This addition makes the manuscript’s statistical declaration unambiguous and fully aligned with our response to the statistical comment. The manuscript now clearly describes the exploratory nature of the data and the absence of formal multiplicity correction, and we have verified that no other section of the manuscript contains a conflicting statement on this point.

We are grateful to the Editor for catching this inconsistency and for giving us the opportunity to correct it before resubmission. The revision is highlighted in red.
(7) The manuscript is now much closer to being suitable for publication. I do not think additional major experiments are necessary, but the remaining issues concerning translational interpretation, cytotoxicity, statistical caution, and wording should be addressed carefully.
Response: We are very grateful for your encouraging assessment and for confirming that no additional major experiments are needed. We also appreciate your guidance on the remaining issues, which we have taken seriously. Following your suggestion, we went through the entire manuscript once more and made a number of careful adjustments to ensure that our interpretation, statistical reporting, and wording are all appropriately cautious and consistent.

For translational interpretation, we softened several statements that could have been read as going beyond what our data can support. For instance, the Introduction now says that we seek to “explore the potential mechanisms linking aspartame to AD” instead of “uncover the potential pathological mechanisms.” In the Results, the molecular docking section no longer describes all targets as showing “stable binding interactions”; it now states that the targets display “varying binding affinities, with GSK3β and KDR showing the strongest interactions and VEGFA exhibiting weaker binding.” We also removed the word “sustained” from the NO response description, since our experiment did not measure long-term effects. For cytotoxicity, we have made it explicit in both the Discussion and the Limitations that the 1 mg/mL concentration reduced BV2 viability to approximately 63%, that cellular stress or cytotoxicity may have contributed to the inflammatory readouts, and that this concentration should not be called sub-cytotoxic. We also suggest that future studies test lower, non-cytotoxic concentrations with proper controls. For statistical caution, we confirmed that the Statistical Analysis section now clearly states that no formal correction for multiple comparisons was applied, that the reported p-values are nominal, and that the qPCR data are exploratory and hypothesis-generating given the three biological replicates and the absence of a formal power analysis. Finally, we removed the remaining overstatement in the Conclusion regarding “the potential risks of long-term aspartame consumption for individuals at high risk of AD.” The Conclusion now says that the study provides a basis for further investigation and highlights the need for future studies using physiologically relevant exposure models.

We believe these changes make the manuscript’s conclusions fully supported by the data and appropriately cautious. Thank you again for your helpful guidance. The revision is highlighted in red.
