# Supplementary material for: Unveiling the neuroinflammatory potential of aspartame in Alzheimer’s disease: A comprehensive network toxicology and experimental validation approach
Source: PLoS One. 2026 Nov 3;21(11):e0361766. doi: 10.1371/journal.pone.0361766 (PMC13730326; doi:10.1371/journal.pone.0361766)
Supplement: Supplementary file 3 [file pone.0361766.s004.docx]

PONE-D-26-23444R2

Unveiling the Neuroinflammatory Potential of Aspartame in Alzheimer's Disease: A Comprehensive Network Toxicology and Experimental Validation Approach

PLOS One

Dear Dr. Sheng,

Thank you for submitting your manuscript to PLOS One. After careful consideration, we feel that it has merit but does not fully meet PLOS One’s publication criteria as it currently stands. Therefore, we invite you to submit a revised version of the manuscript that addresses the points raised during the review process.

Please submit your revised manuscript by Nov 12 2026 11:59PM. If you will need more time than this to complete your revisions, please reply to this message or contact the journal office at plosone@plos.org. When you're ready to submit your revision, log on to https://www.editorialmanager.com/pone/ and select the 'Submissions Needing Revision' folder to locate your manuscript file.

Please include the following items when submitting your revised manuscript:

A letter that responds to each point raised by the academic editor and reviewer(s). You should upload this letter as a separate file labeled 'Response to Reviewers'.

A marked-up copy of your manuscript that highlights changes made to the original version. You should upload this as a separate file labeled 'Revised Manuscript with Track Changes'.

An unmarked version of your revised paper without tracked changes. You should upload this as a separate file labeled 'Manuscript'.

If you would like to make changes to your financial disclosure, please include your updated statement in your cover letter. Guidelines for resubmitting your figure files are available below the reviewer comments at the end of this letter.

If applicable, we recommend that you deposit your laboratory protocols in protocols.io to enhance the reproducibility of your results. Protocols.io assigns your protocol its own identifier (DOI) so that it can be cited independently in the future. For instructions see: https://journals.plos.org/plosone/s/submission-guidelines#loc-laboratory-protocols. Additionally, PLOS One offers an option for publishing peer-reviewed Lab Protocol articles, which describe protocols hosted on protocols.io. Read more information on sharing protocols at https://plos.org/protocols?utm_medium=editorial-email&utm_source=authorletters&utm_campaign=protocols.

As the corresponding author, your ORCID iD is verified in the submission system and will appear in the published article. PLOS supports the use of ORCID, and we encourage all coauthors to register for an ORCID iD and use it as well. Please encourage your coauthors to verify their ORCID iD within the submission system before final acceptance, as unverified ORCID iDs will not appear in the published article. Only the individual author can complete the verification step; PLOS staff cannot verify ORCID iDs on behalf of authors.

We look forward to receiving your revised manuscript.

Kind regards,

António Raposo

Academic Editor

PLOS One

Additional Editor Comments:

Thank you for taking the time to revise the manuscript again. I have read the revised version together with your responses to my previous comments, and I appreciate the care you have taken in addressing the concerns that remained after the last round.

I am particularly pleased to see that the interpretation of the experimental findings is now much more cautious. The limitations associated with the relatively high aspartame concentrations are clearer, and the manuscript now openly acknowledges that the reduced cell viability at 1 mg/mL makes it difficult to rule out a contribution from cellular stress or cytotoxicity to the inflammatory response. I also think the changes to the statistical section are helpful, especially the clarification that the qPCR findings should be considered exploratory rather than confirmatory. These changes have addressed my main concerns from the previous round.

Response: We sincerely thank you for your encouraging comments and for your careful assessment of our revised manuscript. We are delighted to hear that you are pleased with the more cautious interpretation of the experimental findings and that the main scientific concerns from the previous round have been addressed. We also appreciate your confirmation that no further experiments are required at this stage. We have carefully revised the manuscript according to the remaining points, and we provide a point-by-point response below. All changes have been highlighted in the revised manuscript.

There are just a few points that I think still need some attention.

(1) The first concerns the Abstract. Although the Discussion and Conclusions are now appropriately cautious, a couple of statements in the Abstract still seem somewhat stronger than the data allow. In particular, saying that aspartame “may induce microglial neuroinflammation through multiple signaling pathways” and that the study provides “critical insights into the neurological safety of aspartame” could still be read as extending the findings beyond this particular in vitro model. I would suggest bringing these sentences into line with the more measured language now used elsewhere in the manuscript.

Response: We sincerely thank the Editor for this thoughtful comment. We fully agree that the Abstract should use the same measured language as the Discussion and Conclusions, and we appreciate the Editor pointing out these two statements. We have revised the Abstract accordingly to avoid extending our findings beyond the in vitro model.

In the revised Abstract, the sentence now clearly limits the conclusion to BV2 microglial cells and explicitly states that the relevance to human neurological safety remains to be established. The sentence replaces “critical insights” with “a basis for further investigation,” which is consistent with the cautious tone used throughout the revised manuscript. We believe these changes fully address the Editor’s concern while preserving the scientific message of the study. The revision is highlighted in red from lines 1-4 on page 2.

(2) Along the same lines, I would avoid saying that molecular docking “validated” favorable interactions. Docking provides a prediction of possible binding interactions rather than experimental validation, so a term such as “predicted” or “suggested” would be more accurate.

Response: We sincerely thank the Editor for this helpful comment. The molecular docking provides a prediction of possible binding interactions rather than experimental validation, and we appreciate the suggestion to use more accurate wording. We have therefore replaced “validated” with “predicted” in the Abstract, so that the description of the docking results is consistent throughout the paper. This change ensure that the docking results are described as computational predictions rather than experimental validation, in line with the Editor’s comment. The revision is highlighted in red from line 23 on page 1.

(3) I also noticed that the Results still refer to “pathways significantly associated with aspartame-induced AD.” I think this should be changed. The study does not show that aspartame induces AD, and this wording does not really reflect the more careful interpretation that the authors have adopted in this revision. Referring instead to pathways enriched among the overlapping aspartame- and AD-associated targets would avoid this problem.

Response: We sincerely thank the Editor for this careful observation. We agree that the phrase “pathways significantly associated with aspartame-induced AD” is inaccurate, because our study does not demonstrate that aspartame induces AD. We have therefore revised this sentence to refer to pathways enriched among the overlapping aspartame- and AD-associated targets, which more accurately reflects the exploratory nature of our analysis. We are grateful to the Editor for helping us align the terminology with the more cautious interpretation adopted in this revision. The revision is highlighted in red from lines 15-16 on page 10.

(4) There are also two small technical points that would be worth checking. The Methods describe a STRING interaction score of 0.9 as “medium confidence,” which does not seem to be the correct description for such a stringent threshold. In addition, the PPI network is reported as having 143 nodes and 231 edges, followed by “degree of 23.464.” It is not clear to me what this value represents, so I would ask the authors to check the calculation and specify the network metric being reported.

Response: We sincerely thank the Editor for these two careful technical observations. We apologize for the inaccurate description. In the STRING database, the confidence score levels are defined as follows: 0.15 (low confidence), 0.40 (medium confidence), 0.70 (high confidence), and 0.90 (highest confidence). Because we applied a minimum interaction score of 0.9, the correct description is “highest confidence,” not “medium confidence.” We have corrected this in the Methods section.

We also thank the Editor for asking us to clarify this value. The network contains 143 nodes and 231 edges. The quantity we originally reported as “degree of 23.464” was not the average degree. The average degree of an undirected network is defined as: ⟨k⟩ = 2L / N, where L is the number of edges and N is the number of nodes. For our network, this gives: ⟨k⟩ = 2 × 231 / 143 ≈ 3.23. This means that, on average, each node in the PPI network is connected to approximately 3.23 other nodes. We have corrected the Results section to report the correct metric and have explicitly named it as the average degree, so that its meaning is unambiguous. The revision is highlighted in red from lines 12-13 on page 5, lines 4-5 on page 9.

We are grateful to the Editor for catching these errors. We have carefully verified all other numerical values and network metrics in the manuscript to ensure that no similar inconsistencies remain.

(5) Finally, there are still a few grammatical and typographical issues throughout the text. Nothing here affects my assessment of the work, but another careful read through the manuscript would be worthwhile before publication.

Response: We sincerely thank the reviewer for this careful reading. We have performed another thorough read-through of the entire manuscript and corrected the grammatical and typographical issues throughout the text. All language-related corrections have been highlighted in red in the revised manuscript for easy identification. These changes are limited to grammar, wording, and typographical formatting and do not alter the scientific content, data interpretation, or conclusions of the study. We hope the revised manuscript is now more polished and suitable for publication.

I appreciate the way the authors have responded to the comments throughout the revision process. The main scientific concerns I raised previously have, in my view, been addressed, and I do not see a need for further experiments at this stage. The remaining points are relatively minor and should be straightforward to correct.

Response: We sincerely thank the reviewer for the positive and encouraging comments. We are grateful that the main scientific concerns raised in the previous round have been addressed and that no further experiments are deemed necessary at this stage. We have carefully addressed all remaining minor points, including the grammatical and typographical issues, and all corresponding revisions have been highlighted in red in the revised manuscript. We hope the revised manuscript is now acceptable for publication.
