# Supplementary material for: Unveiling the neuroinflammatory potential of aspartame in Alzheimer’s disease: A comprehensive network toxicology and experimental validation approach
Source: PLoS One. 2026 Nov 3;21(11):e0361766. doi: 10.1371/journal.pone.0361766 (PMC13730326; doi:10.1371/journal.pone.0361766)
Supplement: Supplementary file 4 [file pone.0361766.s005.docx]

PONE-D-26-23444R3

Unveiling the Neuroinflammatory Potential of Aspartame in Alzheimer's Disease: A Comprehensive Network Toxicology and Experimental Validation Approach

PLOS One

Dear Dr. Sheng,

Thank you for submitting your manuscript to PLOS One. After careful consideration, we feel that it has merit but does not fully meet PLOS One’s publication criteria as it currently stands. Therefore, we invite you to submit a revised version of the manuscript that addresses the points raised during the review process.

Please submit your revised manuscript by Oct 30 2026 11:59PM. If you will need more time than this to complete your revisions, please reply to this message or contact the journal office at plosone@plos.org. When you're ready to submit your revision, log on to https://www.editorialmanager.com/pone/ and select the 'Submissions Needing Revision' folder to locate your manuscript file.

Please include the following items when submitting your revised manuscript:

A letter that responds to each point raised by the academic editor and reviewer(s). You should upload this letter as a separate file labeled 'Response to Reviewers'.

A marked-up copy of your manuscript that highlights changes made to the original version. You should upload this as a separate file labeled 'Revised Manuscript with Track Changes'.

An unmarked version of your revised paper without tracked changes. You should upload this as a separate file labeled 'Manuscript'.

If you would like to make changes to your financial disclosure, please include your updated statement in your cover letter. Guidelines for resubmitting your figure files are available below the reviewer comments at the end of this letter.

If applicable, we recommend that you deposit your laboratory protocols in protocols.io to enhance the reproducibility of your results. Protocols.io assigns your protocol its own identifier (DOI) so that it can be cited independently in the future. For instructions see: https://journals.plos.org/plosone/s/submission-guidelines#loc-laboratory-protocols. Additionally, PLOS One offers an option for publishing peer-reviewed Lab Protocol articles, which describe protocols hosted on protocols.io. Read more information on sharing protocols at https://plos.org/protocols?utm_medium=editorial-email&utm_source=authorletters&utm_campaign=protocols.

As the corresponding author, your ORCID iD is verified in the submission system and will appear in the published article. PLOS supports the use of ORCID, and we encourage all coauthors to register for an ORCID iD and use it as well. Please encourage your coauthors to verify their ORCID iD within the submission system before final acceptance, as unverified ORCID iDs will not appear in the published article. Only the individual author can complete the verification step; PLOS staff cannot verify ORCID iDs on behalf of authors.

We look forward to receiving your revised manuscript.

Kind regards,

António Raposo

Academic Editor

PLOS One

Journal Requirements:

1. If the reviewer comments include a recommendation to cite specific previously published works, please review and evaluate these publications to determine whether they are relevant and should be cited. There is no requirement to cite these works unless the editor has indicated otherwise.

2. Please review your reference list to ensure that it is complete and correct. If you have cited papers that have been retracted, please include the rationale for doing so in the manuscript text, or remove these references and replace them with relevant current references. Any changes to the reference list should be mentioned in the rebuttal letter that accompanies your revised manuscript. If you need to cite a retracted article, indicate the article’s retracted status in the References list and also include a citation and full reference for the retraction notice.

Additional Editor Comments:

Thank you for taking the time to revise the manuscript again. I have read the new version alongside your responses, and the changes have addressed most of the points raised in my previous assessment. The Abstract is more cautious, and the corrections to the STRING confidence threshold and average network degree are clear.

A few passages still need attention before the manuscript can be accepted.

(1) First, some of the wording corrected in the Abstract and Results remains elsewhere in the paper. The Introduction still says that molecular docking was used to “validate the binding affinities” (page 3, lines 25–27), and the Methods still refer to “aspartame-induced AD” (page 5, line 16). Please revise these passages too. Docking predicts possible interactions, and the present study does not show that aspartame induces Alzheimer’s disease.

Response: We sincerely thank the Editor for pointing this out. The wording in the Introduction and Methods should be consistent with the more careful language used elsewhere in the revised manuscript. Molecular docking can only predict possible binding interactions rather than experimentally validate them, and our study does not demonstrate that aspartame induces Alzheimer’s disease. We have therefore revised both passages accordingly. In the Introduction, we changed the sentence “Molecular docking simulations were subsequently performed to validate the binding affinities between aspartame and its targets” to “Molecular docking simulations were subsequently performed to predict the possible binding interactions between aspartame and its targets.” In the Methods, we changed the phrase “potential targets involved in aspartame-induced AD” to “potential targets overlapping between aspartame and AD,” which more accurately reflects that these targets are shared between the two datasets rather than evidence that aspartame causes AD. These changes are fully consistent with the revised Abstract, Results, and Discussion. The modified text has been highlighted in red from line 26 on page 3 and lines 16-17 on page 5 for your convenience. We are grateful to the Editor for helping us eliminate these remaining inconsistencies.

(2) The point I would particularly like you to check concerns GSK-3β protein expression. The Discussion refers to its experimental assessment on page 19, lines 25–26, and later states that GSK-3β and downstream cytokines were upregulated at both protein and mRNA levels (page 20, lines 19–20). I could find the qPCR results for GSK-3β, but I could not find a corresponding protein assay or protein expression results. If only GSK-3β mRNA was measured, please state this consistently and remove the references to its protein expression. The cytokine measurements by ELISA can be described separately.

Response: We sincerely thank the Editor for this careful and important observation. GSK-3β protein expression was not measured; only its mRNA level was assessed. After re-checking the relevant passages, we confirm that we did not perform any protein assay for GSK-3β. The only protein-level measurements in our study were the ELISA-based quantification of secreted cytokines (TNF-α, IL-1β, and IL-6), which are separate from GSK-3β.

We have therefore revised the Discussion to state consistently that GSK-3β was assessed only at the mRNA level. Specifically, in the paragraph comparing our work with Ge et al., the sentence “showing that aspartame upregulates GSK-3β and downstream cytokines at both the protein and mRNA levels” has been changed to “showing that aspartame upregulates GSK-3β mRNA and increases the secretion and mRNA expression of downstream cytokines (TNF-α, IL-1β, and IL-6).” In the Limitations section, the phrase “The GSK-3β activation and pro-inflammatory response we observed” has been changed to “The GSK-3β mRNA upregulation and pro-inflammatory response we observed.” We have also checked the rest of the manuscript and removed any other wording that could be read as referring to GSK-3β protein expression or activation. The cytokine measurements by ELISA are now described separately and clearly, without linking them to GSK-3β protein. All modified text has been highlighted in red from line 19-20 on page 20, and 24 on page 21 in the revised manuscript. We are grateful to the Editor for catching this inconsistency, which has helped us make the manuscript more accurate and transparent.

(3) This also affects a couple of mechanistic statements. The phrases “robust elucidation” of the NO/oxidative stress–GSK-3β/NF-κB axis (page 20, lines 1–3) and “GSK-3β activation […] we observed” (page 21, line 24) go beyond the measurements presented. An increase in GSK-3β mRNA does not establish activation of the protein. Please describe the proposed pathway as a possible explanation for the findings, rather than a mechanism demonstrated by this study.

Response: We sincerely thank the Editor for this thoughtful comment. We agree that an increase in GSK-3β mRNA does not establish activation of the protein, and we should not have used language implying that the NO/oxidative stress–GSK-3β/NF-κB axis was definitively elucidated or that GSK-3β activation was observed. We have therefore revised these mechanistic statements to present the proposed pathway as a possible explanation rather than a demonstrated mechanism. In the Discussion, the sentence “This targeted focus enabled robust elucidation of the NO/oxidative stress-GSK-3β/NF-κB cytokine signaling axis” has been changed to “This targeted focus allowed us to explore a possible involvement of the NO/oxidative stress–GSK-3β/NF-κB cytokine signaling axis.” Similarly, the phrase “The GSK-3β activation and pro-inflammatory response we observed” has been changed to “The GSK-3β mRNA upregulation and pro-inflammatory response we observed.” We have also checked the rest of the manuscript for any other wording that could be read as claiming GSK-3β activation and have revised it accordingly. All modified text has been highlighted in red from lines 1- 2, page 20. We are grateful to the Editor for helping us keep our mechanistic interpretation strictly within the limits of our data.

(4) Finally, please give the text and figure legends another careful read, particularly the punctuation around the Figure 3 citation and the significance symbols and thresholds in the Figure 8 legend.

Response: We sincerely thank the Editor for this final careful reminder. We have now given the text and all figure legends another thorough read, with particular attention to the punctuation around the Figure 3 citation and to the significance symbols and thresholds in the Figure 8 legend. In the Results, the Figure 3 citation previously read “... overlapping aspartame- and AD-associated targets. (Fig 3). The 10 most enriched pathways...”, which was awkwardly punctuated and placed the citation after a full stop. We have corrected this to “... overlapping aspartame- and AD-associated targets (Fig 3). The 10 most enriched pathways...”, so that the citation now sits naturally before the closing period. For Figure 8, we have carefully revised the entire legend; the significance symbols and thresholds have been revised to “**P < 0.01, ****P < 0.0001”. We have also checked the other figure legends for similar issues and have made the necessary corrections. All modified text has been highlighted in red in the revised manuscript for your convenience. We are grateful to the Editor for encouraging this final careful read, which has helped us improve the accuracy and professionalism of the manuscript.
