# Supplementary material for: Unveiling the neuroinflammatory potential of aspartame in Alzheimer’s disease: A comprehensive network toxicology and experimental validation approach
Source: PLoS One. 2026 Nov 3;21(11):e0361766. doi: 10.1371/journal.pone.0361766 (PMC13730326; doi:10.1371/journal.pone.0361766)
Supplement: Supplementary file 5 [file pone.0361766.s006.docx]

PONE-D-26-23444R4

Unveiling the Neuroinflammatory Potential of Aspartame in Alzheimer's Disease: A Comprehensive Network Toxicology and Experimental Validation Approach

PLOS One

Dear Dr. Sheng,

Thank you for submitting your manuscript to PLOS One. After careful consideration, we feel that it has merit but does not fully meet PLOS One’s publication criteria as it currently stands. Therefore, we invite you to submit a revised version of the manuscript that addresses the points raised during the review process.

Please submit your revised manuscript by Nov 08 2026 11:59PM. If you will need more time than this to complete your revisions, please reply to this message or contact the journal office at plosone@plos.org. When you're ready to submit your revision, log on to https://www.editorialmanager.com/pone/ and select the 'Submissions Needing Revision' folder to locate your manuscript file.

Please include the following items when submitting your revised manuscript:

A letter that responds to each point raised by the academic editor and reviewer(s). You should upload this letter as a separate file labeled 'Response to Reviewers'.

A marked-up copy of your manuscript that highlights changes made to the original version. You should upload this as a separate file labeled 'Revised Manuscript with Track Changes'.

An unmarked version of your revised paper without tracked changes. You should upload this as a separate file labeled 'Manuscript'.

If you would like to make changes to your financial disclosure, please include your updated statement in your cover letter. Guidelines for resubmitting your figure files are available below the reviewer comments at the end of this letter.

If applicable, we recommend that you deposit your laboratory protocols in protocols.io to enhance the reproducibility of your results. Protocols.io assigns your protocol its own identifier (DOI) so that it can be cited independently in the future. For instructions see: https://journals.plos.org/plosone/s/submission-guidelines#loc-laboratory-protocols. Additionally, PLOS One offers an option for publishing peer-reviewed Lab Protocol articles, which describe protocols hosted on protocols.io. Read more information on sharing protocols at https://plos.org/protocols?utm_medium=editorial-email&utm_source=authorletters&utm_campaign=protocols.

As the corresponding author, your ORCID iD is verified in the submission system and will appear in the published article. PLOS supports the use of ORCID, and we encourage all coauthors to register for an ORCID iD and use it as well. Please encourage your coauthors to verify their ORCID iD within the submission system before final acceptance, as unverified ORCID iDs will not appear in the published article. Only the individual author can complete the verification step; PLOS staff cannot verify ORCID iDs on behalf of authors.

We look forward to receiving your revised manuscript.

Kind regards,

António Raposo

Academic Editor

PLOS One

Journal Requirements:

If the reviewer comments include a recommendation to cite specific previously published works, please review and evaluate these publications to determine whether they are relevant and should be cited. There is no requirement to cite these works unless the editor has indicated otherwise.

Please review your reference list to ensure that it is complete and correct. If you have cited papers that have been retracted, please include the rationale for doing so in the manuscript text, or remove these references and replace them with relevant current references. Any changes to the reference list should be mentioned in the rebuttal letter that accompanies your revised manuscript. If you need to cite a retracted article, indicate the article’s retracted status in the References list and also include a citation and full reference for the retraction notice.

Additional Editor Comments:

There are still a few passages that need attention before I can accept the manuscript.

(1) The main remaining issue is in the Discussion (page 20, lines 19–26). Although the wording about protein expression has been corrected, this paragraph still refers to “functional confirmation,” “downstream cytokines,” a “shared neuroinflammatory mechanism,” and effects occurring “through a GSK-3β-associated pathway.” These expressions go beyond what was measured. The experiments show an increase in GSK-3β mRNA alongside changes in inflammatory markers, but they do not establish that GSK-3β mediates those changes. Similarly, the resemblance to the LPS response does not demonstrate that the two treatments act through the same mechanism. Please revise this paragraph to present GSK-3β involvement as a possible explanation that requires further testing.

Response: We sincerely thank the Editor for this careful and important comment. The experiments show an increase in GSK-3β mRNA alongside changes in inflammatory markers, but they do not demonstrate that GSK-3β mediates those changes, and the resemblance to the LPS response does not prove that the two treatments act through the same mechanism. We have therefore revised this paragraph to present GSK-3β involvement as a possible explanation that requires further testing. Specifically, we replaced “functional confirmation” with “further investigation,” removed the term “downstream cytokines” and simply referred to the measured cytokines, changed the statement about a “shared neuroinflammatory mechanism” to a more cautious description of a similar direction of response without claiming a common mechanism, and replaced “through a GSK-3β-associated pathway” with a description of GSK-3β mRNA upregulation as a possible associated feature whose mediating role remains to be tested. The revised paragraph now reads as follows, with the changes highlighted in red (page 20, lines 21-30). We believe this revision keeps our interpretation strictly within the limits of the data and fully addresses the Editor’s concern.

(2) The same point applies to the phrase “provide a valid proof-of-concept for a mechanistic pathway” in the Limitations section (page 21, lines 25–27). Please describe this more cautiously as preliminary evidence of an inflammatory response accompanied by increased GSK-3β mRNA expression. The Conclusion should follow the same approach: “systematically elucidate” and “through a multi-target network in vitro” still suggest that the proposed mechanisms have been established experimentally.

Response: We sincerely thank the Editor for this further careful comment. We agree that the phrase “provide a valid proof-of-concept for a mechanistic pathway” in the Limitations section overstates what our data can support, and that the words “systematically elucidate” and “through a multi-target network in vitro” in the Conclusion still imply that the proposed mechanisms have been experimentally established. Our experiments show an inflammatory response accompanied by increased GSK-3β mRNA expression, but they do not establish a mechanistic pathway or a multi-target network in vitro. We have therefore revised both the Limitations and the Conclusion to describe our findings more cautiously as preliminary evidence of an inflammatory response accompanied by increased GSK-3β mRNA expression, and to present the proposed mechanisms as hypotheses that require further testing. All modified text has been highlighted in red (page 21, lines 28-29; page 22, lines 16, 25-26) in the revised manuscript. We are grateful to the Editor for helping us keep our conclusions strictly within the limits of our data.

(3) Please also check the molecular docking Results (page 12, lines 11–16). The sentence beginning “Our results demonstrated that aspartame forms…” should describe predicted interactions, and the discussion of binding affinities should make clear that these are computational estimates.

Response: We thank the Editor for this additional careful observation. In the original sentence, “Our results demonstrated that aspartame forms hydrogen bonds and strong electrostatic interactions with its protein targets” implied experimental observation, which docking cannot provide. Likewise, the listing of binding energies did not make clear that these values are calculated predictions. We have therefore revised this passage accordingly. The first sentence now reads: “Our docking analysis predicted that aspartame may form hydrogen bonds and electrostatic interactions with its protein targets.” The second sentence now reads: “The predicted binding energies of aspartame with CCNE1 (-4.049 kcal/mol), CDKN1A (-3.837 kcal/mol), CDKN1B (-3.356 kcal/mol), ERBB2 (-3.019 kcal/mol), GSK-3β (-5.002 kcal/mol), KDR (-5.613 kcal/mol), NOS3 (-4.046 kcal/mol), and VEGFA (-2.06 kcal/mol) are computational estimates that indicate varying binding affinities, with the most favorable predicted binding energies observed for GSK-3β and KDR and weaker predicted binding for VEGFA.” These changes make it clear that the docking results are predictions and that the binding energies are computational estimates, in line with the Editor’s comment. All modified text has been highlighted in red in the revised manuscript (page 12, lines 11-17). We are grateful to the Editor for helping us describe the docking results with appropriate precision.

(4) Finally, there is an incomplete sentence in the functional enrichment Methods (page 5, lines 19–21), beginning “Key pathways overlapping aspartame- and AD-associated targets threshold…”. Please restore the sentence and specify the significance threshold used, including whether it refers to nominal or adjusted p-values.

Response: We thank the Editor for catching this incomplete sentence. We apologize for the error. We have now restored the sentence and have also specified the significance threshold clearly, including whether it refers to nominal or adjusted p-values. In our KEGG enrichment analysis using the DAVID database, we used a Benjamini-Hochberg adjusted p-value < 0.05 as the threshold for identifying significantly enriched pathways. This is an adjusted p-value, not a nominal p-value. We have therefore revised the Methods sentence to read as follows, with the restored and clarified text highlighted in red: “Key pathways overlapping aspartame- and AD-associated targets were determined via KEGG enrichment analysis. A Benjamini–Hochberg adjusted p-value < 0.05 was used as the significance threshold to control the false discovery rate (FDR), and all visualizations were performed using the SRplot platform” This revision restores the grammatical structure of the sentence and makes explicit that the threshold refers to adjusted p-values (page 5, lines 20-22). All modified text has been highlighted in red in the revised manuscript. We are grateful to the Editor for identifying this error and for giving us the opportunity to correct it.
