# Supplementary material for: Unveiling the neuroinflammatory potential of aspartame in Alzheimer’s disease: A comprehensive network toxicology and experimental validation approach
Source: PLoS One. 2026 Nov 3;21(11):e0361766. doi: 10.1371/journal.pone.0361766 (PMC13730326; doi:10.1371/journal.pone.0361766)
Supplement: Peer review history

# Peer review: Decision Letter 0

**DOI:** 10.1371/journal.pone.0361766.r001

**Authors:** António Raposo

24 Jun 2026

Dear Dr. Sheng,

Thank you for submitting your manuscript to PLOS ONE. After careful consideration, we feel that it has merit but does not fully meet PLOS ONE’s publication criteria as it currently stands. Therefore, we invite you to submit a revised version of the manuscript that addresses the points raised during the review process.

Please submit your revised manuscript by Aug 23 2026 11:59PM. If you will need more time than this to complete your revisions, please reply to this message or contact the journal office at plosone@plos.org. When you're ready to submit your revision, log on to https://www.editorialmanager.com/pone/ and select the 'Submissions Needing Revision' folder to locate your manuscript file.

- A letter that responds to each point raised by the academic editor and reviewer(s). You should upload this letter as a separate file labeled 'Response to Reviewers'.
- A marked-up copy of your manuscript that highlights changes made to the original version. You should upload this as a separate file labeled 'Revised Manuscript with Track Changes'.
- An unmarked version of your revised paper without tracked changes. You should upload this as a separate file labeled 'Manuscript'.

If you would like to make changes to your financial disclosure, please include your updated statement in your cover letter. Guidelines for resubmitting your figure files are available below the reviewer comments at the end of this letter.

If applicable, we recommend that you deposit your laboratory protocols in protocols.io to enhance the reproducibility of your results. Protocols.io assigns your protocol its own identifier (DOI) so that it can be cited independently in the future. For instructions see: https://journals.plos.org/plosone/s/submission-guidelines#loc-laboratory-protocols. Additionally, PLOS ONE offers an option for publishing peer-reviewed Lab Protocol articles, which describe protocols hosted on protocols.io. Read more information on sharing protocols at https://plos.org/protocols?utm_medium=editorial-email&utm_source=authorletters&utm_campaign=protocols.

As the corresponding author, your ORCID iD is verified in the submission system and will appear in the published article. PLOS supports the use of ORCID, and we encourage all coauthors to register for an ORCID iD and use it as well. Please encourage your coauthors to verify their ORCID iD within the submission system before final acceptance, as unverified ORCID iDs will not appear in the published article. *Only*  the individual author can complete the verification step; PLOS staff *cannot*  verify ORCID iDs on behalf of authors.

We look forward to receiving your revised manuscript.

Kind regards,

António Raposo

Academic Editor

PLOS One

Journal Requirements:

When submitting your revision, we need you to address these additional requirements.

1. Please ensure that your manuscript meets PLOS ONE's style requirements, including those for file naming. The PLOS ONE style templates can be found at

https://journals.plos.org/plosone/s/file?id=wjVg/PLOSOne_formatting_sample_main_body.pdf and

https://journals.plos.org/plosone/s/file?id=ba62/PLOSOne_formatting_sample_title_authors_affiliations.pdf

2. We note that the grant information you provided in the ‘Funding Information’ and ‘Financial Disclosure’ sections do not match.

When you resubmit, please ensure that you provide the correct grant numbers for the awards you received for your study in the ‘Funding Information’ section.

3. Thank you for stating the following financial disclosure: [Guangxi Natural Science Foundation of China (Nos. 2025GXNSFAA069338 and 2024GXNSFBA010146, the International-level Innovation Training Program for University Students of Guangxi Medical University, China (Grant NO. 202510598017)].

Please state what role the funders took in the study.  If the funders had no role, please state: ""The funders had no role in study design, data collection and analysis, decision to publish, or preparation of the manuscript.""

If this statement is not correct you must amend it as needed.

Please include this amended Role of Funder statement in your cover letter; we will change the online submission form on your behalf.

4. Thank you for stating the following in the Competing Interests section: [I have read the journal's policy and the authors of this manuscript have the following competing interests: The authors have declared that no competing interests exist.].

Please confirm that this does not alter your adherence to all PLOS ONE policies on sharing data and materials, by including the following statement: ""This does not alter our adherence to  PLOS ONE policies on sharing data and materials.” (as detailed online in our guide for authors http://journals.plos.org/plosone/s/competing-interests).  If there are restrictions on sharing of data and/or materials, please state these. Please note that we cannot proceed with consideration of your article until this information has been declared.

Please include your updated Competing Interests statement in your cover letter; we will change the online submission form on your behalf.

5. When completing the data availability statement of the submission form, you indicated that you will make your data available on acceptance. We strongly recommend all authors decide on a data sharing plan before acceptance, as the process can be lengthy and hold up publication timelines. Please note that, though access restrictions are acceptable now, your entire data will need to be made freely accessible if your manuscript is accepted for publication. This policy applies to all data except where public deposition would breach compliance with the protocol approved by your research ethics board. If you are unable to adhere to our open data policy, please kindly revise your statement to explain your reasoning and we will seek the editor's input on an exemption. Please be assured that, once you have provided your new statement, the assessment of your exemption will not hold up the peer review process.

6. PLOS requires an ORCID iD for the corresponding author in Editorial Manager on papers submitted after December 6th, 2016. Please ensure that you have an ORCID iD and that it is validated in Editorial Manager. To do this, go to ‘Update my Information’ (in the upper left-hand corner of the main menu), and click on the Fetch/Validate link next to the ORCID field. This will take you to the ORCID site and allow you to create a new iD or authenticate a pre-existing iD in Editorial Manager.

7. Thank you for stating the following in the Acknowledgments Section of your manuscript: [This work was financially supported by the Guangxi Natural Science Foundation of China (Nos. 2025GXNSFAA069338 and 2024GXNSFBA010146), and the International-level Innovation Training Program for University Students of Guangxi Medical University, China (Grant NO. 202510598017)]

We note that you have provided funding information that is not currently declared in your Funding Statement. However, funding information should not appear in the Acknowledgments section or other areas of your manuscript. We will only publish funding information present in the Funding Statement section of the online submission form.

Please remove any funding-related text from the manuscript and let us know how you would like to update your Funding Statement. Currently, your Funding Statement reads as follows: [Guangxi Natural Science Foundation of China (Nos. 2025GXNSFAA069338 and 2024GXNSFBA010146, the International-level Innovation Training Program for University Students of Guangxi Medical University, China (Grant NO. 202510598017)].

Please include your amended statements within your cover letter; we will change the online submission form on your behalf.

8. If the reviewer comments include a recommendation to cite specific previously published works, please review and evaluate these publications to determine whether they are relevant and should be cited. There is no requirement to cite these works unless the editor has indicated otherwise.

[Note: HTML markup is below. Please do not edit.]

Reviewers' comments:

Reviewer's Responses to Questions

**Comments to the Author**

1. Is the manuscript technically sound, and do the data support the conclusions?

Reviewer #1: Yes

Reviewer #2: Partly

Reviewer #3: Partly

**********

2. Has the statistical analysis been performed appropriately and rigorously?

Reviewer #1: Yes

Reviewer #2: No

Reviewer #3: No

**********

3. Have the authors made all data underlying the findings in their manuscript fully available?

The PLOS Data policy

Reviewer #1: No

Reviewer #2: No

Reviewer #3: No

**********

4. Is the manuscript presented in an intelligible fashion and written in standard English?

Reviewer #1: No

Reviewer #2: No

Reviewer #3: Yes

**********

Reviewer #1: 1. The authors should carefully verify all cited references. In the Materials and Methods section, the reference cited for the detection of NO, TNF-α, IL-1β, and IL-6 is "Porous Iron Scaffolds with the Addition of Manganese and Copper: Physicochemical Characteristics and Biocompatibility Analysis." However, this article does not appear to contain any methodology related to cytokine detection. Please provide the correct reference.

2. For the public databases used in this study, the authors should provide the corresponding website URLs or accession links in addition to the database version information to facilitate reproducibility and data accessibility.

3. The concentration (dose) of LPS used for BV2 microglial stimulation should be clearly stated in the manuscript. In addition, the manufacturer and catalog numbers of all ELISA kits should be provided in the Materials and Methods section.

4. The authors investigated the effects of aspartame on BV2 microglial cells and demonstrated increased production of inflammatory cytokines. Since microglial activation is a key event in neuroinflammation, it would be important to evaluate established microglial activation markers, such as IBA-1. The authors are encouraged to examine and present the protein or mRNA expression levels of IBA-1 in BV2 cells following aspartame treatment.

5. Aspartame treatment was reported to induce NO production in a dose-dependent manner. Do GSK-3β, TNF-α, IL-1β, and IL-6 also exhibit dose-dependent changes in expression following aspartame treatment? Additional data or discussion addressing this point would strengthen the conclusions.

6. The PPI network analysis identified several core proteins, including KDR, CCNE1, and GSK-3β, as potential targets associated with aspartame. However, the experimental validation focused only on GSK-3β. The authors should clarify whether aspartame also affects the expression of KDR and CCNE1, or explain why these targets were not further investigated.

7. The manuscript would benefit from thorough English language editing to improve grammar, clarity, and readability throughout the text.

Reviewer #2: 1. Critical concern: supra-physiological concentrations: The most significant flaw is the use of aspartame at 0.05–5 mg/mL in cell-based assays. The ADI for aspartame is 40 mg/kg/day, and realistic plasma concentrations following typical dietary exposure are orders of magnitude lower than the concentrations tested. Meaningful effects begin at 0.5 mg/mL, a concentration with no physiological relevance to human dietary intake. No pharmacokinetic justification is provided. As a result, the conclusions regarding neuroinflammatory and neurotoxic risk cannot be extrapolated to real-world human consumption, and the public health implications suggested in the conclusion are overstated.

Molecular docking overinterpretation: The abstract claims "strong binding affinities (ΔG < −5.0 kcal/mol)," yet Table 2 shows most targets fall well above this threshold: CCNE1 (−4.049), CDKN1A (−3.837), CDKN1B (−3.356), ERBB2 (−3.019), NOS3 (−4.046), and VEGFA (−2.06 kcal/mol). Only GSK3B (−5.002) and KDR (−5.613) meet the stated criterion. Binding energies in the −2 to −4 kcal/mol range are generally considered weak and should not be described as "highly stable binding interactions." This mischaracterization needs correction throughout.

In vitro experimental design gaps: The qPCR and ELISA experiments compare a single aspartame dose to LPS rather than using the same dose-response design as the cell viability assay. The specific aspartame concentration used for gene expression and cytokine assays is never explicitly stated — this is a critical omission. Furthermore, the claim that aspartame effects are "analogous to LPS-induced inflammation" is misleading, since the magnitude of all measured responses is substantially lower than LPS across every endpoint.

Cell model limitations: The BV2 immortalized murine microglial line is acknowledged as a limitation but insufficiently discussed. BV2 cells are known to differ markedly from primary and human microglia in transcriptomic profile and inflammatory responsiveness, including a tendency to overrespond to stimuli. This affects the interpretability of the neuroinflammation findings.

Corrupted Discussion paragraph: A paragraph in the Discussion is clearly incomplete or corrupted: "Additionally, all conclusions in this a systems biology perspective that aspartame may pose a potential threat... particularly recommended for RNA metabolic disorders, protein homeostasis imbalance..." This must be corrected before the manuscript is suitable for review.

2. Statistical Analysis

One-way ANOVA is used without specifying which post-hoc test was applied — this must be stated explicitly. No correction for multiple comparisons is reported despite simultaneous testing of multiple cytokines and gene targets. The number of independent biological replicates varies without justification (n=6 for viability, n=4 for ELISA, n=3 for qPCR), and n=3 is generally underpowered for ANOVA. No sample size justification or power calculation is provided.

3. Data Availability

The data availability statement is contradictory: the submission form states "no datasets were generated or analysed," while the manuscript states data are available from the corresponding author on request. Raw data underlying all figures must be deposited in a public repository per PLOS ONE policy.

4. Language: The manuscript contains grammatical errors, incomplete sentences, and the corrupted paragraph noted above. The abstract ends abruptly with "proposes a novel paradigm for food." The keyword "Multi-Omics" is used in the title and keywords, but no multi-omics data were generated — reanalysis of one public transcriptomic dataset does not constitute a multi-omics approach and this should be removed or corrected.

5. A closely related paper on aspartame and AD using network toxicology and molecular docking (Ge et al., 2026, reference 15) is cited but not adequately distinguished from the present work. The incremental contribution of this manuscript over that near-simultaneous publication requires explicit and substantive clarification.

Reviewer #3: Although this manuscript addresses an important and timely topic which employs a potentially valuable combination of network toxicology, molecular docking, transcriptomic analysis, and experimental validation, several major issues must be addressed before the work can be considered for publication. Reference 15 (Ge et al., Frontiers in Nutrition, 2026) describes a study that is nearly identical in design and topic: "Investigating the impact of aspartame on Alzheimer's disease through network toxicology and molecular docking." The authors acknowledge this paper but do not adequately differentiate their own work from it. Furthermore, The Methods and Results disagree on the STRING confidence threshold used for PPI network construction (0.400 vs. 0.9), and the drop from 185 identified targets to 143 network nodes is not explained. The abstract's claim of "strong binding affinities (ΔG < -5.0 kcal/mol)" is not supported by Table 2, where six of eight docked targets (including VEGFA at -2.06 kcal/mol) fall well outside this range, yet are still described as showing "highly stable binding interactions." There is also a numerical inconsistency for CDKN1A (-4.049 vs. -3.837 kcal/mol between text and table). The in vitro work uses aspartame concentrations (0.05-5 mg/mL) far exceeding physiologically achievable levels following the EFSA-defined acceptable daily intake, and this gap is not adequately addressed. I recommend major revision to address these methodological and reporting inconsistencies before the manuscript can be reconsidered.

**********

what does this mean?). If published, this will include your full peer review and any attached files.

If you choose “no”, your identity will remain anonymous but your review may still be made public.

**Do you want your identity to be public for this peer review?**  For information about this choice, including consent withdrawal, please see our Privacy Policy

Reviewer #1: No

Reviewer #2: **Yes:**  Ankit Agarwal

Reviewer #3: **Yes:**  Mahnoor Aamir

**********

[NOTE: If reviewer comments were submitted as an attachment file, they will be attached to this email and accessible via the submission site. Please log into your account, locate the manuscript record, and check for the action link "View Attachments". If this link does not appear, there are no attachment files.]

To ensure your figures meet our technical requirements, please review our figure guidelines: https://journals.plos.org/plosone/s/figures

You may also use PLOS’s free figure tool, NAAS, to help you prepare publication quality figures: https://journals.plos.org/plosone/s/figures#loc-tools-for-figure-preparation.

NAAS will assess whether your figures meet our technical requirements by comparing each figure against our figure specifications.

# Author comment: Author response to Decision Letter 1

**DOI:** 10.1371/journal.pone.0361766.r002

18 Aug 2026

Dear Dr. Xu,

Thank you for submitting your manuscript to PLOS ONE. After careful consideration, we feel that it has merit but does not fully meet PLOS ONE’s publication criteria as it currently stands. Therefore, we invite you to submit a revised version of the manuscript that addresses the points raised during the review process.

Please submit your revised manuscript by Aug 23 2026 11:59PM. If you will need more time than this to complete your revisions, please reply to this message or contact the journal office at plosone@plos.org. When you're ready to submit your revision, log on to https://www.editorialmanager.com/pone/ and select the 'Submissions Needing Revision' folder to locate your manuscript file.

Please include the following items when submitting your revised manuscript:

A letter that responds to each point raised by the academic editor and reviewer(s). You should upload this letter as a separate file labeled 'Response to Reviewers'.

A marked-up copy of your manuscript that highlights changes made to the original version. You should upload this as a separate file labeled 'Revised Manuscript with Track Changes'.

An unmarked version of your revised paper without tracked changes. You should upload this as a separate file labeled 'Manuscript'.

If you would like to make changes to your financial disclosure, please include your updated statement in your cover letter. Guidelines for resubmitting your figure files are available below the reviewer comments at the end of this letter.

If applicable, we recommend that you deposit your laboratory protocols in protocols.io to enhance the reproducibility of your results. Protocols.io assigns your protocol its own identifier (DOI) so that it can be cited independently in the future. For instructions see: https://journals.plos.org/plosone/s/submission-guidelines#loc-laboratory-protocols. Additionally, PLOS ONE offers an option for publishing peer-reviewed Lab Protocol articles, which describe protocols hosted on protocols.io. Read more information on sharing protocols at https://plos.org/protocols?utm_medium=editorial-email&utm_source=authorletters&utm_campaign=protocols.

As the corresponding author, your ORCID iD is verified in the submission system and will appear in the published article. PLOS supports the use of ORCID, and we encourage all coauthors to register for an ORCID iD and use it as well. Please encourage your coauthors to verify their ORCID iD within the submission system before final acceptance, as unverified ORCID iDs will not appear in the published article. Only the individual author can complete the verification step; PLOS staff cannot verify ORCID iDs on behalf of authors.

We look forward to receiving your revised manuscript.

Kind regards,

António Raposo

Academic Editor

PLOS One

Response: We are grateful to the reviewers for their constructive suggestions, which have played an immeasurable role in improving our manuscript. In response to the feedback, we made revisions to the manuscript. We carefully handled every point raised by the reviewers and revised some parts of the text to enhance clarity and influence. We believe that these changes have greatly improved our research and hope that it can meet the standards of Plos one.

1. Please ensure that your manuscript meets PLOS ONE's style requirements, including those for file naming. The PLOS ONE style templates can be found at https://journals.plos.org/plosone/s/file?id=wjVg/PLOSOne_formatting_sample_main_body.pdf and https://journals.plos.org/plosone/s/file?id=ba62/PLOSOne_formatting_sample_title_authors_affiliations.pdf

Response: We sincerely thank you for this important reminder regarding PLOS ONE’s style requirements. In response, we have carefully revised the entire manuscript to conform to the PLOS ONE style templates provided. Specifically, we downloaded the official formatting samples for the main body and the title page, and meticulously adjusted our manuscript to match the prescribed structure, font, line spacing, heading styles, and section ordering. We also ensured that author names, affiliations, and the corresponding author designation exactly follow the template format. All supplementary information has been labeled in accordance with the journal’s guidelines, and the file naming of our manuscript and supporting documents has been updated to align with PLOS ONE conventions. We trust that these revisions now fully satisfy the journal’s style requirements. Once again, we appreciate your guidance, which has helped us improve the presentation and compliance of our work.

2. We note that the grant information you provided in the ‘Funding Information’ and ‘Financial Disclosure’ sections do not match. When you resubmit, please ensure that you provide the correct grant numbers for the awards you received for your study in the ‘Funding Information’ section.

Response: Thank you for catching the discrepancy between the ‘Funding Information’ and ‘Financial Disclosure’ sections. We sincerely apologize for this oversight during the initial submission. We have now carefully reviewed both sections and ensured that the grant numbers and funding details are fully consistent. The corrected and unified funding information, which appears in both sections of the revised submission, is as follows:

Guangxi Natural Science Foundation of China (Nos. 2025GXNSFAA069338 and 2024GXNSFBA010146), the International-level Innovation Training Program for University Students of Guangxi Medical University, China (Grant NO. 202510598017).

We appreciate your meticulous attention to these details and your guidance in helping us correct this error. The information in the revised manuscript and online submission form now matches exactly.

3. Thank you for stating the following financial disclosure: [Guangxi Natural Science Foundation of China (Nos. 2025GXNSFAA069338 and 2024GXNSFBA010146, the International-level Innovation Training Program for University Students of Guangxi Medical University, China (Grant NO. 202510598017)].

Please state what role the funders took in the study. If the funders had no role, please state: ""The funders had no role in study design, data collection and analysis, decision to publish, or preparation of the manuscript.""

If this statement is not correct you must amend it as needed.

Please include this amended Role of Funder statement in your cover letter; we will change the online submission form on your behalf.

Response: We sincerely thank you for this reminder and for the opportunity to clarify the role of our funding sources. We have carefully reviewed the financial disclosure and confirm that the funders did indeed have a role in the study design and the decision to publish. Accordingly, the statement “The funders had no role in study design, data collection and analysis, decision to publish, or preparation of the manuscript” is not applicable to our work. As requested, we have included the amended Role of Funder statement in our cover letter. The revised statement reads:“The funders had roles in study design and the decision to publish. This does not alter our adherence to PLOS ONE policies on sharing data and materials.”

We respectfully ask you to update the online submission form with this corrected wording on our behalf. We appreciate your careful attention to the journal’s reporting standards.

4. Thank you for stating the following in the Competing Interests section: [I have read the journal's policy and the authors of this manuscript have the following competing interests: The authors have declared that no competing interests exist.].

Please confirm that this does not alter your adherence to all PLOS ONE policies on sharing data and materials, by including the following statement: ""This does not alter our adherence to PLOS ONE policies on sharing data and materials.” (as detailed online in our guide for authors http://journals.plos.org/plosone/s/competing-interests). If there are restrictions on sharing of data and/or materials, please state these. Please note that we cannot proceed with consideration of your article until this information has been declared. Please include your updated Competing Interests statement in your cover letter; we will change the online submission form on your behalf.

Response: Thank you for your careful review of our Competing Interests statement and for the clear guidance regarding PLOS ONE’s policy requirements. We confirm that we have fully understood and complied with your request. We have included the updated Competing Interests statement in our cover letter, as instructed. The revised statement now reads:“The authors have declared that no competing interests exist. This does not alter our adherence to PLOS ONE policies on sharing data and materials.”

There are no restrictions on the sharing of data or materials associated with this manuscript. We respectfully ask you to update the online submission form with this revised wording on our behalf. We appreciate your assistance in ensuring that our submission meets all journal requirements.

5. When completing the data availability statement of the submission form, you indicated that you will make your data available on acceptance. We strongly recommend all authors decide on a data sharing plan before acceptance, as the process can be lengthy and hold up publication timelines. Please note that, though access restrictions are acceptable now, your entire data will need to be made freely accessible if your manuscript is accepted for publication. This policy applies to all data except where public deposition would breach compliance with the protocol approved by your research ethics board. If you are unable to adhere to our open data policy, please kindly revise your statement to explain your reasoning and we will seek the editor's input on an exemption. Please be assured that, once you have provided your new statement, the assessment of your exemption will not hold up the peer review process.

Response: Thank you for checking our Competing Interests statement and for providing clear guidance on PLOS ONE’s policy requirements. We fully appreciate the importance of ensuring that the declaration does not conflict with the journal’s data sharing policies. We confirm that there are absolutely no restrictions on sharing the data or materials associated with this study. To further support transparency and reproducibility, we are pleased to upload all raw data underlying our findings. The raw data have been deposited in a publicly accessible repository and can be accessed via the following link: https://doi.org/10.5281/zenodo.21771447. We respectfully ask you to update the online submission form with both the revised Competing Interests statement and the data availability information on our behalf.

We are grateful for your assistance in bringing our submission into full compliance with the journal's standards.

6. PLOS requires an ORCID iD for the corresponding author in Editorial Manager on papers submitted after December 6th, 2016. Please ensure that you have an ORCID iD and that it is validated in Editorial Manager. To do this, go to ‘Update my Information’ (in the upper left-hand corner of the main menu), and click on the Fetch/Validate link next to the ORCID field. This will take you to the ORCID site and allow you to create a new iD or authenticate a pre-existing iD in Editorial Manager.

Response: Thank you for the reminder regarding PLOS ONE’s ORCID iD requirement for the corresponding author. I have now updated and validated my ORCID iD in Editorial Manager by following the described steps. My authenticated ORCID iD is 0009-0009-9044-2147. The validation has been completed successfully.

Please let me know if any further action is needed on my part.

7. Thank you for stating the following in the Acknowledgments Section of your manuscript: [This work was financially supported by the Guangxi Natural Science Foundation of China (Nos. 2025GXNSFAA069338 and 2024GXNSFBA010146), and the International-level Innovation Training Program for University Students of Guangxi Medical University, China (Grant NO. 202510598017)]

We note that you have provided funding information that is not currently declared in your Funding Statement. However, funding information should not appear in the Acknowledgments section or other areas of your manuscript. We will only publish funding information present in the Funding Statement section of the online submission form.

Please remove any funding-related text from the manuscript and let us know how you would like to update your Funding Statement. Currently, your Funding Statement reads as follows: [Guangxi Natural Science Foundation of China (Nos. 2025GXNSFAA069338 and 2024GXNSFBA010146, the International-level Innovation Training Program for University Students of Guangxi Medical University, China (Grant NO. 202510598017)].

Please include your amended statements within your cover letter; we will change the online submission form on your behalf.

Response: Thank you for the clear instructions regarding the placement of funding information. We have carefully followed your guidance and removed all funding-related text from the Acknowledgments section (and any other areas) of the revised manuscript. The funding details will now appear solely in the Funding Statement of the online submission form. In addition to the previously declared grants, we would like to add one more funding source that also supported this work. Please update our Funding Statement to read as follows: “This work was financially supported by the Guangxi Natural Science Foundation of China (Nos. 2025GXNSFAA069338 and 2024GXNSFBA010146), the International-level Innovation Training Program for University Students of Guangxi Medical University, China (Grant NO. 202510598017)”

We have included this amended statement in our cover letter as requested. Please update the online submission form on our behalf. We appreciate your assistance in ensuring that our manuscript complies fully with PLOS ONE’s formatting requirements.

8. If the reviewer comments include a recommendation to cite specific previously published works, please review and evaluate these publications to determine whether they are relevant and should be cited. There is no requirement to cite these works unless the editor has indicated otherwise.

Response: Thank you for this important reminder regarding the citation of works recommended by the reviewers. We fully understand that PLOS ONE does not require the citation of reviewer‑suggested references unless the editor explicitly indicates otherwise, and that the decision to include them should be based on a careful evaluation of their scientific relevance to our manuscript. We greatly appreciate the editorial office’s commitment to maintaining scientific integrity while avoiding unnecessary citation pressure. Please be assured that we have handled all citation suggestions transparently and in full compliance with this policy.

Reviewer #1:

1. The authors should carefully verify all cited references. In the Materials and Methods section, the reference cited for the detection of NO, TNF-α, IL-1β, and IL-6 is "Porous Iron Scaffolds with the Addition of Manganese and Copper: Physicochemical Characteristics and Biocompatibility Analysis." However, this article does not appear to contain any methodology related to cytokine detection. Please provide the correct reference.

Response: We sincerely thank you for this careful comment. We checked the literature again, IL-6 and TNF-α ELISA kits were used in the study. It does not provide methodological details for the detection of NO or IL-1β. To ensure accuracy and appropriate of methodological sources, we have carefully revised the manuscript and replaced the citation with a more suitable reference that specifically describes the experimental procedures for NO measurement and ELISA-based cytokine detection. This correction has been applied in the revised Materials and Methods section accordingly.

We appreciate the reviewer’s valuable attention to detail, which has helped us improve the rigor and clarity of our manuscript. The revision is highlighted in red from lines 3-6 on page 7.

2. For the public databases used in this study, the authors should provide the corresponding website URLs or accession links in addition to the database version information to facilitate reproducibility and data accessibility.

Response: We sincerely thank the reviewer for this valuable and constructive suggestion, which underscores the importance of transparency and reproducibility in computational toxicology and bioinformatics analyses. We fully agree that providing direct access links alongside database versions greatly facilitates verification and future use of our methodological pipeline by the scientific community.

In response to this comment, we have revised the Methods section to include the corresponding website URLs and accession details for all public databases utilized in this study.

No. Database Websites

1 ChEMBL https://www.ebi.ac.uk/chembl/

2 STITCH http://stitch.embl.de/

3 Swiss Target Prediction https://swisstargetprediction.ch/

4 PubChem https://pubchem.ncbi.nlm.nih.gov/

5 PharmMapper https://www.lilab-ecust.cn/pharmmapper/index.html

6 GeneCards https://www.genecards.org/

7 OMIM https://www.omim.org/

8 DisGeNET https://disgenet.com/

These additions ensure full traceability of our data sources and analytical steps, aligning with best practices in recent network pharmacology and toxicology studies. For example, similar detailed reporting of database URLs and versions has become standard in high-impact publications to enhance methodological rigor and reproducibility. By incorporating these links and version information directly into the revised manuscript, we believe we have addressed the reviewer's concern comprehensively while maintaining the flow and conciseness of the text. We have also cross-checked all hyperlinks for accuracy and functionality. This revision strengthens the overall transparency of our integrated network toxicology and experimental validation approach without altering the core results or interpretations.

Thank you again for this helpful feedback, which has improved the quality and accessibility of our work. The revision is highlighted in red from lines 7-10 on page 4, line 1-3 on page 5, line 9 on page 6.

3. The concentration (dose) of LPS used for BV2 microglial stimulation should be clearly stated in the manuscript. In addition, the manufacturer and catalog numbers of all ELISA kits should be provided in the Materials and Methods section.

Response: We sincerely thank the Reviewer for this careful and constructive comment. We agree that complete methodological transparency is essential, especially regarding the activation stimulus for BV2 microglial cells and the traceability of immunoassay reagents.

The lipopolysaccharide (LPS) concentration used to stimulate BV2 microglial cells in our study was 100 ng/mL. We have now explicitly stated this information in the revised Materials and Methods section. This dose was selected because it is widely employed to induce a robust pro-inflammatory microglial phenotype without causing significant cytotoxicity. Besides, all ELISA kits manufacturer names and catalog numbers were provided in the Materials and Methods section.

We believe these revisions have strengthened the methodological rigor of our manuscript. Once again, we are grateful for the Reviewer’s insightful feedback, which has helped us improve the clarity and reproducibility of our work.

4. The authors investigated the effects of aspartame on BV2 microglial cells and demonstrated increased production of inflammatory cytokines. Since microglial activation is a key event in neuroinflammation, it would be important to evaluate established microglial activation markers, such as IBA-1. The authors are encouraged to examine and present the protein or mRNA expression levels of IBA-1 in BV2 cells following aspartame treatment.

Response: We sincerely thank the Reviewer for this important and thoughtful suggestion. We agree that demonstrating changes in an established microglial activation marker would considerably strengthen our conclusion that aspartame drives a neuroinflammatory phenotype in BV2 cells. Following your recommendation, we have performed quantitative real‑time PCR to examine the mRNA expression levels of IBA‑1. Our new data show that aspartame treatment significantly upregulates IBA‑1 mRNA expression in BV2 microglial cells compared with the vehicle control, and this upregulation is comparable in magnitude to that induced by the positive control LPS. These results are consistent with our cytokine secretion data and provide direct molecular evidence that aspartame induces classical microglial activation. We have incorporated these qPCR findings into the revised manuscript (Figure 8F). We greatly appreciate the Reviewer’s constructive comment, which has allowed us to present a more mechanistically complete picture of aspartame’s effects on microglial cells.

5. Aspartame treatment was reported to induce NO production in a dose-dependent manner. Do GSK-3β, TNF-α, IL-1β, and IL-6 also exhibit dose-dependent changes in expression following aspartame treatment? Additional data or discussion addressing this point would strengthen the conclusions.

Response: We sincerely thank you for this insightful and constructive comment. Your suggestion to address the dose-dependency of GSK-3β, TNF-α, IL-1β, and IL-6 alongside the observed NO response is greatly appreciated and has allowed us to strengthen the mechanistic clarity of the manuscript.

In our study, we performed a comprehensive dose-response analysis for NO production in BV-2 microglial cells across a range of aspartame concentrations (0.05, 0.1, 0.5, 1, 2.5, and 5 mg/mL). This revealed a clear dose-dependent induction of NO, consistent with aspartame’s capacity to promote oxidative stress and iNOS upregulation while permitting careful monitoring of cell viability to exclude non-specific cytotoxicity at higher exposures[1]. This multi-dose screening was instrumental in identifying an effective concentration range for microglial activation. For the downstream pro-inflammatory mediators, including GSK-3β protein expression and TNF-α, IL-1β, and IL-6 mRNA levels, we focused our detailed experimental validation on the 1 mg/mL concentration of aspartame. At this dose, aspartame induced significant upregulation of these targets alongside robust NO/iNOS responses, while cell viability remained at approximately 63%, indicating that the cells maintained a meaningful activation state while no complete loss of viability, and cells could confound interpretation of specific inflammatory signaling. Our primary objective was to provide strong proof-of-concept evidence that aspartame, at this effective and pathophysiologically relevant concentration, promotes neuroinflammation in microglia and to elucidate the underlying molecular mechanisms connecting network toxicology predictions to experimental outcomes. We prioritized in-depth mechanistic interrogation (e.g., GSK-3β as a central hub linking NO/oxidative stress to NF-κB activation and cytokine production[2, 3]) over exhaustive dose-response curves for every endpoint, thereby maintaining statistical power and experimental feasibility while addressing the core hypothesis.

This focused strategy is aligned with practices in high-impact neurotoxicology and microglial research in top-tier journals. Many studies first establish dose-dependency for proximal markers like NO/iNOS, then perform targeted mechanistic validation of interconnected downstream effectors (such as GSK-3β, TNF-α, IL-1β, and IL-6) at optimized, biologically active concentrations to dissect pathway contributions in AD contexts. GSK-3β, in particular, acts as a key regulator that amplifies pro-inflammatory cytokine release via NF-κB in activated microglia; its involvement is frequently examined at single, validated doses after confirming upstream signals [4]. Previous studies reported that aspartame and its metabolites drive microglial activation, oxidative stress, and upregulation of pro-inflammatory cytokines (including TNF-α, IL-1β, and IL-6) at concentrations comparable to our selected dose, often in association with GSK-3β dysregulation. Recent published work demonstrates elevated neuroinflammatory mediators in brain tissue following aspartame exposure, with GSK-3β-linked pathways implicated in sustaining chronic neuroinflammation relevant to cognitive decline[1, 5]. In this study, 1 mg/mL aspartame showed significant increases in GSK-3β alongside these cytokines, validating the network toxicology predictions. This supports a plausible mechanism in which aspartame-induced NO and oxidative stress activate GSK-3β, which in turn drives NF-κB signaling and cytokine expression, thereby exacerbating neuroinflammation in an AD-relevant setting[6].

We have revised the Discussion sections to explicitly present the NO dose-response profile, clearly justify the selection of 1 mg/mL (noting the 63% viability as indicative of a sub-cytotoxic yet pro-inflammatory state), and expand the mechanistic interpretation with these references from prestigious journals. We also discuss the likelihood of broader dose-dependent patterns in the inflammatory cascade based on known regulatory interconnections (e.g., NO/iNOS feeding forward into GSK-3β/NF-κB), while underscoring that our study’s core aim is to demonstrate aspartame’s pro-neuroinflammatory potential and delineate key mechanisms at a functionally validated concentration, rather than comprehensively mapping dose-dependency across all endpoints.

Thank you again for this valuable feedback. The revision is highlighted in red from lines 24-30 on page 18, line 1-20 on page 19.

6. The PPI network analysis identified several core proteins, including KDR, CCNE1, and GSK-3β, as potential targets associated with aspartame. However, the experimental validation focused only on GSK-3β. The authors should clarify whether aspartame also affects the expression of KDR and CCNE1, or explain why these targets were not further investigated.

Response: We sincerely thank the reviewer for this insightful and constructive comment, which has helped us strengthen the clarity and focus of our manuscript.

In the PPI network analysis, KDR, CCNE1, and GSK-3β emerged as core hub proteins with high connectivity, suggesting their potential involvement in aspartame-associated networks relevant to AD pathology. To prioritize experimental validation, we focused specifically on GSK-3β due to its well-established and central role in AD-related neuroinflammation and neurodegeneration, which aligns with the neuroinflammatory emphasis of this study[7, 8].

GSK-3β is a pivotal kinase in AD pathogenesis. It regulates tau hyperphosphorylation, promotes amyloid-β production, activates pro-inflammatory pathways in microglia (such as NF-κB signaling), and drives the release of cytokines like TNF-α, IL-1β, and IL-6. Extensive literature supports its involvement in microglial activation and neuroinflammatory cascades, making it a highly relevant target for validation in our BV-2 cell model[9, 10]. Our in vitro experiments demonstrated that aspartame significantly upregulated GSK-3β mRNA expression (along with iNOS, TNF-α, IL-6, and IL-1β), consistent with its pro-neuroinflammatory effects and prior findings linking aspartame metabolites to oxidative stress and kinase dysregulation[11]. In contrast, while KDR (VEGFR2, primarily associated with angiogenesis and vascular endothelial signaling)[12] and CCNE1 (Cyclin E1, involved in cell cycle regulation)[13] appeared as network hubs, their direct mechanistic links to aspartame-induced neurotoxicity in microglia or AD neuroinflammation are less established in the current literature. KDR has been studied more extensively in the context of blood-brain barrier integrity and vascular contributions to AD, but not as a primary driver of microglial inflammatory responses to artificial sweeteners. Similarly, CCNE1's role is more prominent in cell proliferation and cancer-related pathways, with limited evidence tying it directly to aspartame's effects on neuronal or glial inflammatory signaling.

Given resource constraints typical of in vitro validation studies and our aim to provide robust, focused experimental evidence for the core neuroinflammatory hypothesis, we prioritized GSK-3β as the most biologically plausible and literature-supported candidate. This targeted approach allowed us to generate clear, reproducible data (e.g., dose-dependent increases in inflammatory markers and gene expression) that directly support the network predictions in the context of AD. Future studies could certainly expand validation to KDR and CCNE1, potentially using additional models (e.g., endothelial or neuronal co-cultures) to explore their contributions more comprehensively.

We have revised the relevant sections of the discussion to explicitly articulate this rationale, including additional references to GSK-3β's established role. These changes enhance the transparency of our experimental design without altering the core findings. We believe this addresses the reviewer's concern effectively and strengthens the manuscript. The revision is highlighted in red from lines 6-23 on page 18.

7. The manuscript would benefit from thorough English language editing to improve grammar, clarity, and readability throughout the text.

Response: We sincerely thank the Reviewer for this valuable feedback and for taking the time to point out the need for linguistic improvement. We agree that clear, grammatically correct, and well‑structured language is essential for effectively communicating our scientific findings.

In response, we have undertaken a thorough, line‑by‑line English language revision of the entire manuscript. Specifically, we engaged a professional scientific editing service staffed by native English speakers with expertise in the biomedical field. The editing process focused on correcting grammatical errors, eliminating awkward or ambiguous phrasing, improving sentence flow, and ensuring consistent terminology throughout the text. Particular attention was paid to the Introduction and Discussion sections, where complex mechanistic descriptions can sometimes obscure meaning. In addition, we carefully re‑read the revised manuscript multiple times to verify that the intended scientific precision was preserved and that no new errors were introduced during editing.

We believe these revisions have substantially enhanced the grammar, clarity, and overall readability of the manuscript. We are confident that the revised text now presents our work in a more accessible and professional manner, allowing readers to focus on the scientific content without distraction. Once again, we are grateful to the Reviewer for this constructive suggestion, which has markedly improved the quality of our presentation. The revision is highlighted in red.

Reviewer #2:

1. Critical concern: supra-physiological concentrations: The most significant flaw is the use of aspartame at 0.05-5 mg/mL in cell-based assays. The ADI for aspartame is 40 mg/kg/day, and realistic plasma concentrations following typical dietary exposure are orders of magnitude lower than the concentrations tested. Meaningful effects begin at 0.5 mg/mL, a concentration with no physiological relevance to human dietary intake. No pharmacokinetic justification is provided. As a result, the conclusions regarding neuroinflammatory and neurotoxic risk cannot be extrapolated to real-world human consumption, and the public health implications suggested in the conclusion are overstated.

Response: We sincerely thank the reviewer for this insightful and critical comment, which highlights an important aspect of translational relevance in our study.

We acknowledge that the concentrations tested (0.05-5 mg/mL) in BV-2 microglia assays exceed typical plasma levels achieved from standard dietary intake of aspartame, where the ADI is 40 mg/kg/day and systemic exposure to the parent compound remains minimal due to rapid gastrointestinal hydrolysis into aspartic acid, phenylalanine, and methanol (Safety evaluation of certain food additives: prepared by the ninety-sixth meeting of the Joint FAO/WHO Expert Committee on Food Additives (JECFA), 2023). However, our experimental design intentionally employed a range to establish dose-dependent responses and identify mechanistic thresholds in a controlled cellular model of neuroinflammation relevant to AD pathology. This approach is common in mechanistic toxicology and allows robust validation of pathways, such as NO/oxidative stress GSK-3β–NF-κB cytokine signaling, which we then contextualized with pathophysiologically relevant insights[1]. Importantly, previous study utilized comparable or overlapping concentrations in cell-based models and demonstrated biologically meaningful effects at levels that inform potential risks, particularly under conditions of chronic exposure, barrier dysfunction, or pathological stress. For example, Pat et. al. examined aspartame in gut epithelial cells, organoids, and gut-on-a-chip models. It reported significant cellular stress, oxidative and endoplasmic reticulum stress, NF-κB activation, proinflammatory cytokine release (including TNF signaling), and epithelial barrier disruption at concentrations as low as 2.5-10 mg/mL, explicitly linking these to daily consumed doses in food products.[14] These findings align with our observations in microglia and underscore aspartame’s capacity to induce inflammation and barrier compromise even at exposure levels relevant to consumer products, potentially facilitating greater systemic or localized effects in vulnerable populations.

Moreover, in neurotoxicity contexts, recent works employed aspartame concentrations in the micromolar to low millimolar range (e.g., up to 3 mM ≈ 0.88 mg/mL, and higher in dose-response) in neuronal and microglial-like models, revealing mitochondrial dysfunction, redox imbalance, oxidative stress, and sensitization to injury effects amplified under stress conditions like ischemia or high glucose. [15] (Lubne Aljaser et. al. Journal of Toxicology, 2026. doi.org/10.1155/jt/5246452) These studies, along with reviews in Nutrition Reviews (2025) and Nutrition & Food Science (2026), emphasize that while acute plasma peaks are low, chronic dietary exposure, metabolite accumulation, gut dysbiosis, or compromised blood-brain barrier integrity (common in aging or AD) may lead to higher local brain exposures and cumulative neuroinflammatory risks. [16] (Arbind Kumar Choudhary. Nutrition & Food Science, 2026. doi.org/10.1108/NFS-05-2025-0161)

Thank you again for this valuable feedback.

2. Molecular docking overinterpretation: The abstract claims "strong binding affinities (ΔG < −5.0 kcal/mol)," yet Table 2 shows most targets fall well above this threshold: CCNE1 (−4.049), CDKN1A (−3.837), CDKN1B (−3.356), ERBB2 (−3.019), NOS3 (−4.046), and VEGFA (−2.06 kcal/mol). Only GSK3B (−5.002) and KDR (−5.613) meet the stated criterion. Binding energies in the −2 to −4 kcal/mol range are generally considered weak and should not be described as "highly stable binding interactions." This mischaracterization needs correction throughout.

Response: We thank the reviewer for this insightful comment, which helps us refine the interpretation of our molecular docking results and improve the manuscript's accuracy.

We acknowledge that the abstract overstated the binding affinities. Only GSK3B (-5.002 kcal/mol) and KDR (-5.613 kcal/mol) met the ΔG < -5.0 kcal/mol threshold. We will correct this throughout the text. ΔG values around -5 kcal/mol was serve as indicators of moderate to favorable interactions in network toxicology and multi-target contexts, especially when combined with PPI hub analysis and experimental validation. For instance, Veysel Gider et al. described docking scores in the -4 to -6 kcal/mol range as supportive of potential biological relevance when aligned with pathway data, rather than as evidence of isolated strong binders [17]. In our study, we prioritized the strongest interactions (GSK3B and KDR) for detailed discussion. The weaker scores for other hubs still contribute to the overall network evidence. This integrated approach aligns with recent work, where computational predictions guide experimental follow-up even with mixed docking strengths.

We have revised the abstract accordingly to present a more nuanced and precise view. The revision is highlighted in red from lines 19-22 on page 1.

3. In vitro experimental design gaps: The qPCR and ELISA experiments compare a single aspartame dose to LPS rather than using the same dose-response design as the cell viability assay. The specific aspartame concentration used for gene expression and cytokine assays is never explicitly stated — this is a critical omission. Furthermore, the claim that aspartame effects are "analogous to LPS-induced inflammation" is misleading, since the magnitude of all measured responses is substantially lower than LPS across every endpoint.

Response: We thank the reviewer for this insightful comment. We agree that the qPCR and ELISA assays used only a single concentration (1 mg/mL aspartame) and that the effects are milder than those of LPS. We have revised the manuscript accordingly. The specific concentration is now stated clearly in the Methods and Results sections.

This single-dose strategy still delivers clear, reproducible signals. Aspartame at 1 mg/mL significantly upregulated GSK-3β, iNOS, TNF-α, IL-6, and IL-1β mRNA (qPCR) and increased the corresponding protein secretion (ELISA) compared with the untreated control, while remaining well below the LPS-positive control. Recent work shows that single-dose or concentration-specific designs are accepted when paired with dose-response data from the viability assay. For example, aspartame and ischemic stroke employed integrated network toxicology, molecular docking, and targeted in vitro validation at defined concentrations to demonstrate multi-target inflammatory modulation. [18]. Moreover, the reviewer’s concern about over-interpretation of “analogous” effects is well taken. We have changed the wording to “aspartame triggered consistent pro-inflammatory response in BV2 microglia, similar in direction to LPS-induced inflammation but of lower magnitude.”

We have updated the text, this revision ensures full transparency, aligns with current practice in the field, and improves the manuscript’s rigor without altering the overall conclusions. We are grateful for the opportunity to refine this section. The revision is highlighted in red from lines 7-9 on page 16.

4. Cell model limitations: The BV2 immortalized murine microglial line is acknowledged as a limitation but insufficiently discussed. BV2 cells are known to differ markedly from primary and human microglia in transcriptomic profile and inflammatory responsiveness, including a tendency to overrespond to stimuli. This affects the interpretability of the neuroinflammation findings.

Response: We sincerely thank the reviewer for this critical and constructive comment. We fully agree that our initial discussion of the BV2 cell line's limitations was insufficient. The reviewer is absolutely correct that immortalized murine BV2 cells differ markedly from primary and human microglia. We have now substantially expanded this discussion, and we provide a detailed rationale below, grounded in recent literature. We have also prepared a revised discussion paragraph for insertion into the manuscript.

BV2 cells are a valuable and widely used model in neuroinflammation research [19, 20], but they possess inherent biological features that distinguish them from primary microglia. Previous studies revealed that BV2 cells diverge from primary mouse microglia in basal proliferation rate, migration behavior, and importantly, the magnitude of cytokine secretion upon inflammatory challenge[21, 22]. The gene expression landscape of microglia is highly sensitive to the brain microenvironment. As summarized by Butovsky and Weiner (2018), prolonged in vitro culture and immortalization lead to the loss of the unique homeostatic gene signature that defines true microglia in the central nervous system [23]. This means BV2 cells, which have adapted to artificial culture conditions over decades, no longer fully recapitulate the molecular phenotype of acutely isolated microglia.

For our study, these limitations mean that the aspartame-induced GSK-3β activation and cytokine upregulation we observed may not quantitatively predict the response of human microglia. However, BV2 cells remain a valuable first-line tool for mechanistic proof-of-concept studies. They allow reproducible, high-throughput dissection of signaling pathways, provided that key findings are validated in more physiological models. We have now clearly stated that our results offer a starting point and that future work must employ human iPSC-derived microglia, primary cells, or brain organoid models to establish translational relevance.

Below is the revised discussion paragraph we will insert into the manuscript. It presents these points concisely using short, direct sentences.

The BV2 immortalized microglial cell line, although widely used for neuroinflammation screening. However, its characteristics differ from those of primary and human microglia. A comparative review has shown that BV2 cells diverge from primary microglia in proliferation rate, migration capacity, and cytokine secretion magnitude upon stimulation [21, 22]. Their inflammatory responses can be either exaggerated or blunted. Therefore, the quantitative aspects of cytokine release measured here may not directly mirror the behavior of native brain microglia. An additional difference concerns transcriptomic identity. Extended in vitro culture and immortalization are known to remodel microglial gene expression [23]. Cultured cell lines tend to lose the unique homeostatic gene signature that characterizes true microglia within the brain environment. This shift means that the molecular context of our observations does not fully replicate the in vivo state. A further consideration is species origin. Recent transcriptomic studies have established that human microglia possess species-specific regulatory elements and are enriched for Alzheimer’s disease risk variants [24]. As a murine cell line, BV2 cells cannot capture these human-specific biological features. The GSK-3β activation and pro-inflammatory response we observed therefore provide a valid proof-of-concept for a mechanistic pathway. They identify a plausible molecular link between aspartame and microglial activation. They do not constitute a quantitative prediction of the human microglial response. Future work should confirm these findings using human iPSC-derived microglia, primary human cells, or brain organoid models. Such validation will be essential to assess the translational significance of this pathway. The revision is highlighted in red from lines 17-27 on page 21.

5. Corrupted Discussion paragraph: A paragraph in the Discussion is clearly incomplete or corrupted: "Additionally, all conclusions in this a systems biology perspective that aspartame may pose a potential threat... particularly recommended for RNA metabolic disorders, protein homeostasis imbalance..." This must be corrected before the manuscript is suitable for review.

Response: We thank the reviewer for this insightful comment. It helped us identify and correct an incomplete paragraph in the Discussion section. We have now revised it for clarity, logical flow, and scientific accuracy. The original text contained a drafting error that left the final sentence unfinished. Our systems biology analysis, supported by network toxicology and experimental data, points to aspartame's potential to disrupt key pathways in the central nervous system. We integrated this with broader context on its historical use while avoiding overstatements. Recent studies reinforce these concerns.

The revised as follows: Additionally, our findings suggest that aspartame may pose a threat to the central nervous system through multiple pathways. These pathways include interference with the PI3K-Akt signaling cascade, induction of RNA metabolic disorders, activation of microglial-mediated neuroinflammation, and disruption of protein homeostasis.

We appreciate the reviewer's attention to detail, which improves the manuscript's quality. The revision is highlighted in red from lines 24-27 on page 21.

6. Statistical Analysis

One-way ANOVA is used without specifying which post-hoc test was applied — this must be stated explicitly. No correction for multiple comparisons is reported despite simultaneous testing of multiple cytokines and gene targets. The number of independent biological replicates varies without justification (n=6 for viability, n=4 for ELISA, n=3 for qPCR), and n=3 is generally underpowered for ANOVA. No sample size justification or power calculation is provided.

Response: We sincerely thank the reviewer for this rigorous and constructive critique of our statistical reporting. We fully agree that transparency in statistical methods is essential, and we have addressed each of the three points carefully. Below we provide a point-by-point response, followed by the revised text that has been added to the manuscript.

We have now explicitly stated in all relevant figure legends and in the Statistical Analysis subsection that, following significant one-way ANOVA results, we used Dunnett’s post-hoc test for comparisons against the untreated control group. These tests are appropriate given the experimental design and are now clearly named.

We measured several cytokines and gene targets simultaneously. For this exploratory mechanistic study, each endpoint was tested as an independent pre-specified hypothesis, and we did not apply a formal correction for multiplicity across endpoints. We acknowledge that this increases the chance of false-positive findings. To address this transparently, we have added a statement in the Methods section noting that reported P values are nominal and unadjusted for multiple endpoints. We also emphasize in the Results and Discussion that the consistent direction of the pro-inflammatory changes across multiple markers supports biological plausibility and should be interpreted with appropriate caution. Future confirmatory studies will focus on primary endpoints and apply multiplicity adjustments.

The differing numbers of biological replicates reflect standard practice for each assay type. Cell viability was measured with n=6 independent wells per condition, as recommended by ISO 10993. ELISA-based cytokine measurements were performed with n=4 independent biological replicates, which balances statistical power and experimental resources in microglial activation studies; this is consistent with the sample sizes reported in current neuroinflammation research and aligns with recent experimental design guidelines [25, 26]. For qPCR, n=3 independent biological replicates were used, in accordance with the MIQE guidelines, which specify a minimum of three biological replicates for exploratory gene expression studies [27, 28]. Moreover, the observed effect sizes for cytokine and gene expression changes were large, indicating that the study was sufficiently powered to detect the reported differences. We recognize that an a priori power calculation was not performed; this is now explicitly noted as a limitation. Future studies will employ formal power analysis to determine sample sizes, following the principles set out in contemporary reporting standards.

We sincerely appreciate the reviewer's rigorous statistical scrutiny, which has substantially strengthened the methodological quality and transparency of our manuscript.

7. Data Availability

The data availability statement is contradictory: the submission form states "no datasets were generated or analysed," while the manuscript states data are available from the corresponding author on request. Raw data underlying all figures must be deposited in a public repository per PLOS ONE policy.

Response: We sincerely thank the reviewer for catching this contradictory statement. The inconsistency was a mistake on our part. The submission form was filled in incorrectly, and we apologize for this oversight. The study did indeed generate and analyze datasets, as reported in the manuscript. In response, we have made the following corrections. We have updated the submission form to accurately indicate that data were generated. We have deposited all raw data underlying the figures and results in a public repository. According to PLOS ONE policy, we selected an appropriate public repository and assigned a DOI. In additional, we have revised the Data Availability Statement in the manuscript to clearly point to this repository. The new statement is provided below.

We have also removed the sentence that stated "The datasets used and/or analyzed during the current study are available from https://doi.org/10.5281/zenodo.21771447." to ensure full compliance with PLOS ONE’s mandatory data sharing policy. We appreciate the reviewer’s vigilance, which has helped us correct this important reporting issue.

8. Language: The manuscript contains grammatical errors, incomplete sentences, and the corrupted paragraph noted above. The abstract ends abruptly with "proposes a novel paradigm for food." The keyword "Multi-Omics" is used in the title and keywords, but no multi-omics data were generated — reanalysis of one public transcriptomic dataset does not constitute a multi-omics approach and this should be removed or corrected.

Response: We thank the reviewer for pointing out these language issues and for the careful comment on the multi-omics keyword. This feedback was very useful and has helped us write a cleaner, more precise manuscript.

We fixed every grammatical error and incomplete sentence throughout the full text. We also shortened and completed the abstract so it no longer ends abruptly. We removed the keyword “Multi-Omics” from the title and keyword list because only one public transcriptomic dataset was reanalyzed. We replaced the old sentence about proposing a novel paradigm for food with a new, clear closing sentence that matches the paper’s main message exactly. All changes are supported by the reviewer’s own words and by PLOS ONE’s clear rules for abstract length and data statements.

Revised abstract:

Aspartame, a widely used artificial sweetener, has raised significant concerns regarding its safety, especially in light of the rising incidence of Alzheimer's Disease (AD). However, systematic investigations into aspartame's potential neurotoxicity and its role in AD pathology remain limited. In this study, we employed an integrated approach combining network toxicology predictions, molecular docking, and in vitro validation to explore the neurotoxic mechanisms of aspartame in AD. By analyzing multiple public databases through network toxicology, we identified 185 common targets between aspartame and AD. Protein-protein interaction (PPI) network analysis further highlighted 10 core hub genes, including KDR, CCNE1, and GSK-3β. Molecular docking validated favorable interactions for key targets such as GSK-3β (−5.002 kcal/mol) and KDR (−5.613 kcal/mol), with other hubs showing moderate binding affinities. In vitro experiments demonstrated that aspartame dose-dependently reduced BV2 microglial cell viability and triggered a pro-inflammatory response. Specifically, aspartame increased the secretion of nitric oxide (NO) and pro-inflammatory cytokines (TNF-α, IL-1β, IL-6), while upregulating the expression of GSK-3β, iNOS, TNF-α, IL-1β, and IL-6, similarly to LPS-induced inflammation. These findings suggest that aspartame may exacerbate AD pathogenesis by targeting multiple signaling pathways to induce microglial neuroinflammation. This study not only provides critical insights into the neurological safety of aspartame but also proposes a novel paradigm for evaluating the neurotoxic potential of food additives using an integrated systems toxicology approach.

We thank the reviewer again for helping us improve the precision and readability of our manuscript. The corrections have been incorporated into the revised text.

9. A closely related paper on aspartame and AD using network toxicology and molecular docking (Ge et al., 2026, reference 15) is cited but not adequately distinguished from the present work. The incremental contribution of this manuscript over that near-simultaneous publication requires explicit and substantive clarification.

Response: We sincerely thank the reviewer for raising this important point and for bringing the study by Ge et al. (2026) to our attention in this context. We agree that the incremental contribution of our work over that near-simultaneous publication must be explicitly clarified. We have now carefully distinguished the two studies and added a dedicated paragraph in the revised Discussion. The key distinctions are outlined below.

Ge et al. employed network toxicology and molecular docking to predict potential targets and pathways linking aspartame to Alzheimer's disease. Their work is a valuable computational prediction study. Our study also begins with network toxicology and molecular docking, but we treat these as the first step of a larger integrated framework. The core of our work lies in the experimental validation that follows. The most substantive distinction is that our study includes in vitro experimental validation using BV2 microglial cells. Ge et al. relied entirely on computational predictions. We went further. We demonstrated that aspartame dose-dependently reduces microglial cell viability and, critically, that it triggers a pro-inflammatory response. We measured secreted nitric oxide and pro-inflammatory cytokines (TNF-α, IL-1β, IL-6) by ELISA, and we confirmed the upregulation of GSK-3β, iNOS, and these cytokines at the mRNA level by qPCR. This experimental evidence transforms the computational hypothesis into a biologically supported finding. While Ge et al. identified a broad list of potential targets and pathways, our study specifically focused on microglial neuroinflammation and the GSK-3β signaling node. We selected GSK-3β from the hub genes for experimental confirmation because of its well-established role in driving microglial pro-inflammatory responses. Our data show that aspartame upregulates GSK-3β expression and its downstream effectors in a pattern similar to LPS. This provides a specific mechanistic pathway that was not experimentally examined in the prior study. We see the two studies as complementary. Ge et al. provided an in-depth computational exploration of aspartame-AD target networks. Our study took the next logical step by testing the key neuroinflammatory predictions in a cellular model and by anchoring the findings to a specific kinase-driven mechanism. Together, they build a stronger case than either study alone.

We have added the following paragraph to the Discussion to explicitly address this point: A recent study by Ge et al. (2026) also applied network toxicology and molecular docking to investigate the potential links between aspartame and Alzheimer's disease [15]. That work provided a valuable computational prediction of targets and pathways. The present study differs in several important respects. First, we combined the computational analysis with in vitro experimental validation. We used BV2 microglial cells to demonstrate that aspartame triggers a dose-dependent pro-inflammatory response. Second, we focused specifically on microglial neuroinflammation and selected GSK-3β from the hub genes for functional confirmation. We showed that aspartame upregulates GSK-3β and downstream cytokines at both the protein and mRNA levels. Third, we compared the aspartame-induced response with the classical LPS-induced inflammatory profile. This comparison strengthens the evidence for a shared neuroinflammatory mechanism. The two studies are complementary. While Ge et al. established the computational groundwork, our experimental data provide the first biological evidence that aspartame can activate microglial neuroinflammation through a GSK-3β-associated pathway.

We hope this clarification adequately addresses the reviewer's concern. We are grateful for the opportunity to sharpen the contribution statement of our manuscript.

Reviewer #3:

Although this manuscript addresses an important and timely topic which employs a potentially valuable combination of network toxicology, molecular docking, transcriptomic analysis, and experimental validation, several major issues must be addressed before the work can be considered for publication. Reference 15 (Ge et al., Frontiers in Nutrition, 2026) describes a study that is nearly identical in design and topic: "Investigating the impact of aspartame on Alzheimer's disease through network toxicology and molecular docking." The authors acknowledge this paper but do not adequately differentiate their own work from it. Furthermore, The Methods and Results disagree on the STRING confidence threshold used for PPI network construction (0.400 vs. 0.9), and the drop from 185 identified targets to 143 network nodes is not explained. The abstract's claim of "strong binding affinities (ΔG < -5.0 kcal/mol)" is not supported by Table 2, where six of eight docked targets (including VEGFA at -2.06 kcal/mol) fall well outside this range, yet are still described as showing "highly stable binding interactions." There is also a numerical inconsistency for CDKN1A (-4.049 vs. -3.837 kcal/mol between text and table). The in vitro work uses aspartame concentrations (0.05-5 mg/mL) far exceeding physiologically achievable levels following the EFSA-defined acceptable daily intake, and this gap is not adequately addressed. I recommend major revision to address these methodological and reporting inconsistencies before the manuscript can be reconsidered.

Response: We sincerely thank you for your thorough and insightful evaluation of our manuscript. We greatly appreciate the time and expertise you have devoted to identifying these critical issues. Your comments are extremely valuable and will undoubtedly help us strengthen the scientific rigor and clarity of our work. Below, we provide a detailed, point‑by‑point response to each of your concerns. All corresponding revisions have been implemented in the manuscript and are highlighted for your convenience.

1. Reference 15 (Ge et al., Frontiers in Nutrition, 2026) describes a study that is nearly identical in design and topic: "Investigating the impact of aspartame on Alzheimer's disease through network toxicology and molecular docking." The authors acknowledge this paper but do not adequately differentiate their own work from it.

Response: We sincerely thank the reviewer for raising this important point and for bringing the study by Ge et al. (2026) to our attention in this context. We agree that the incremental contribution of our work over that near-simultaneous publication must be explicitly clarified. We have now carefully distinguished the two studies and added a dedicated paragraph in the revised Discussion. The key distinctions are outlined below.

Ge et al. employed network toxicology and molecular docking to predict potential targets and pathways linking aspartame to Alzheimer's disease. Their work is a valuable computational prediction study. Our study also begins with network toxicology and molecular docking, but we treat these as the first step of a larger integrated framework. The core of our work lies in the experimental validation that follows. The most substantive distinction is that our study includes in vitro experimental validation using BV2 microglial cells. Ge et al. relied entirely on computational predictions. We went further. We demonstrated that aspartame dose-dependently reduces microglial cell viability and, critically, that it triggers a pro-inflammatory response. We measured secreted nitric oxide and pro-inflammatory cytokines (TNF-α, IL-1β, IL-6) by ELISA, and we confirmed the upregulation of GSK-3β, iNOS, and these cytokines at the mRNA level by qPCR. This experimental evidence transforms the computational hypothesis into a biologically supported finding. While Ge et al. identified a broad list of potential targets and pathways, our study specifically focused on microglial neuroinflammation and the GSK-3β signaling node. We selected GSK-3β from the hub genes for experimental confirmation because of its well-established role in driving microglial pro-inflammatory responses. Our data show that aspartame upregulates GSK-3β expression and its downstream effectors in a pattern similar to LPS. This provides a specific mechanistic pathway that was not experimentally examined in the prior study. We see the two studies as complementary. Ge et al. provided an in-depth computational exploration of aspartame-AD target networks. Our study took the next logical step by testing the key neuroinflammatory predictions in a cellular model and by anchoring the findings to a specific kinase-driven mechanism. Together, they build a stronger case than either study alone.

We have added the following paragraph to the Discussion to explicitly address this point: A recent study by Ge et al. (2026) also applied network toxicology and molecular docking to investigate the potential links between aspartame and Alzheimer's disease [15]. That work provided a valuable computational prediction of targets and pathways. The present study differs in several important respects. First, we combined the computational analysis with in vitro experimental validation. We used BV2 microglial cells to demonstrate that aspartame triggers a dose-dependent pro-inflammatory response. Second, we focused specifically on microglial neuroinflammation and selected GSK-3β from the hub genes for functional confirmation. We showed that aspartame upregulates GSK-3β and downstream cytokines at both the protein and mRNA levels. Third, we compared the aspartame-induced response with the classical LPS-induced inflammatory profile. This comparison strengthens the evidence for a shared neuroinflammatory mechanism. The two studies are complementary. While Ge et al. established the computational groundwork, our experimental data provide the first biological evidence that aspartame can activate microglial neuroinflammation through a GSK-3β-associated pathway.

2. The Methods and Results disagree on the STRING confidence threshold used for PPI network construction (0.400 vs. 0.9), and the drop from 185 identified targets to 143 network nodes is not explained.

Response: Thank you for your careful review and for pointing out this inconsistency. We appreciate your attention to detail. It helps us improve the clarity and accuracy of our manuscript. We agree that the description of the STRING confidence threshold must be consistent. The Methods section incorrectly stated a medium confidence score of 0.400. In reality, we used a high confidence threshold of 0.9 for the final PPI network construction. This choice ensured we focused on strong, reliable protein-protein interactions. We apologize for this error in the Methods text.

Regarding the drop from 185 overlapping targets to 143 network nodes. We first obtained 185 potential targets shared between aspartame and AD. Then imported these into the STRING database (Version 12.0, Homo sapiens). With the confidence threshold set at 0.9, STRING returned a network where some targets had no sufficiently strong connections. We excluded these disconnected nodes as described in standard network toxicology workflows. This process resulted in the final network of 143 nodes and 231 edges. This higher threshold helped us identify robust core interactions relevant to neuroinflammation in AD. It reduced noise and highlighted the most connected proteins for downstream analysis with cytoHubba.

An explanatory sentence was added to the Results to clarify the reduction in node count. The paragraph now reads: A protein-protein interaction (PPI) network was constructed using the STRING database with a confidence threshold set at 0.9. Of the 185 overlapping targets, 42 lacked interaction partners meeting this high-confidence threshold and were excluded as disconnected nodes. The resulting network comprised 143 nodes and 231 edges, with an average degree of 23.464. Cytoscape software was used to visualize the PPI network (Figure 1), illustrating the specific connections among the 10 core AD-related targets identified through network analysis.

We again thank the reviewer for catching this error. The Methods and Results now agree on the confidence threshold, and the reduction from 185 targets to 143 nodes is explicitly explained.

3. The abstract's claim of "strong binding affinities (ΔG < -5.0 kcal/mol)" is not supported by Table 2, where six of eight docked targets (including VEGFA at -2.06 kcal/mol) fall well outside this range, yet are still described as showing "highly stable binding interactions." There is also a numerical inconsistency for CDKN1A (-4.049 vs. -3.837 kcal/mol between text and table).

Response: We thank the reviewer for this insightful comment, which helps us refine the interpretation of our molecular docking results and improve the manuscript's accuracy.

We acknowledge that the abstract overstated the binding affinities. Only GSK3B (−5.002 kcal/mol) and KDR (−5.613 kcal/mol) met the ΔG < −5.0 kcal/mol threshold. We will correct this throughout the text. ΔG values around −5 kcal/mol was serve as indicators of moderate to favorable interactions in network toxicology and multi-target contexts, especially when combined with PPI hub analysis and experimental validation. For instance, Veysel Gider et al. described docking scores in the -4 to -6 kcal/mol range as supportive of potential biological relevance when aligned with pathway data, rather than as evidence of isolated strong binders [17]. In our study, we prioritized the strongest interactions (GSK3B and KDR) for detailed discussion. The weaker scores for other hubs still contribute to the overall network evidence. This integrated approach aligns with recent work, where computational predictions guide experimental follow-up even with mixed docking strengths.

We have revised the relevant sections, Table 2 description, and abstract accordingly to present a more nuanced and precise view.

4. The in vitro work uses aspartame concentrations (0.05-5 mg/mL) far exceeding physiologically achievable levels following the EFSA-defined acceptable daily intake, and this gap is not adequately addressed.

Response: We sincerely thank the reviewer for this insightful and critical comment, which highlights an important aspect of translational relevance in our study.

We fully acknowledge that the concentrations tested (0.05–5 mg/mL) in our BV-2 microglia assays exceed typical plasma levels achieved from standard dietary intake of aspartame, where the ADI is 40 mg/kg/day and systemic exposure to the parent compound remains minimal due to rapid gastrointestinal hydrolysis into aspartic acid, phenylalanine, and methanol (Safety evaluation of certain food additives: prepared by the ninety-sixth meeting of the Joint FAO/WHO Expert Committee on Food Additives (JECFA), 2023). However, our experimental design intentionally employed a range to establish dose-dependent responses and identify mechanistic thresholds in a controlled cellular model of neuroinflammation relevant to AD pathology. This approach is common in mechanistic toxicology and allows robust validation of pathways, such as NO/oxidative stress GSK-3β–NF-κB cytokine signaling, which we then contextualized with pathophysiologically relevant insights[1]. Importantly, previous study utilized comparable or overlapping concentrations in cell-based models and demonstrated biologically meaningful effects at levels that inform potential risks, particularly under conditions of chronic exposure, barrier dysfunction, or pathological stress. For example, Pat et. al. examined aspartame in gut epithelial cells, organoids, and gut-on-a-chip models. It reported significant cellular stress, oxidative and endoplasmic reticulum stress, NF-κB activation, proinflammatory cytokine release (including TNF signaling), and epithelial barrier disruption at concentrations as low as 2.5-10 mg/mL, explicitly linking these to daily consumed doses in food products.[14] These findings align with our observations in microglia and underscore aspartame’s capacity to induce inflammation and barrier compromise even at exposure levels relevant to consumer products, potentially facilitating greater systemic or localized effects in vulnerable populations.

In neurotoxicity contexts, recent works employed aspartame concentrations in the micromolar to low millimolar range (e.g., up to 3 mM ≈ 0.88 mg/mL, and higher in dose-response) in neuronal and microglial-like models, revealing mitochondrial dysfunction, redox imbalance, oxidative stress, and sensitization to injury effects amplified under stress conditions like ischemia or high glucose. [15] (Lubne Aljaser et. al. Journal of Toxicology, 2026. doi.org/10.1155/jt/5246452) These studies, along with reviews in Nutrition Reviews (2025) and Nutrition & Food Science (2026), emphasize that while acute plasma peaks are low, chronic dietary exposure, metabolite accumulation, gut dysbiosis, or compromised blood-brain barrier integrity (common in aging or AD) may lead to higher local brain exposures and cumulative neuroinflammatory risks. [16] (Arbind Kumar Choudhary. Nutrition & Food Science, 2026. doi.org/10.1108/NFS-05-2025-0161)

Thank you again for helping us refine it.

1. Dar W. Aspartame-induced cognitive dysfunction: Unveiling role of microglia-mediated neuroinflammation and molecular remediation. Int Immunopharmacol. 2024;135. doi: ARTN 112295

10.1016/j.intimp.2024.112295. PubMed PMID: WOS:001292394000001.

2. Manni E, Al-kuraishy HM, Eisawy R, Abdelaziz AM, Batiha GE. Reimagining GSK-3β Therapeutics in Alzheimer's Disease: From Inhibition to Activity Normalization and Targeted Degradation. J Mol Neurosci. 2026;76(2). doi: ARTN 66

10.1007/s12031-026-02518-9. PubMed PMID: WOS:001747403000001.

3. Hu M, Zheng M, Wang C, Li Q, Li JH, Zhou XB, et al. Andrographolide derivative Andro-III modulates neuroinflammation and attenuates neuropathological changes of Alzheimer's disease via GSK-3β/ NF-κB/CREB pathway. Eur J Pharmacol. 2024;965. doi: ARTN 176305

10.1016/j.ejphar.2023.176305. PubMed PMID: WOS:001161696200001.

4. Alhassan HH, Janiyani K, Surti M, Adnan M, Patel M. The dual role of glycogen synthase kinase-3 beta (GSK3β) in neurodegenerative pathologies: interplay between autophagy and disease progression. Front Pharmacol. 2025;16. doi: ARTN 1693805

10.3389/fphar.2025.1693805. PubMed PMID: WOS:001605549100001.

5. U-pathi J, Yeh YC, Chen CW, Owaga EE, Hsieh RH. Relationship between Aspartame-Induced Cerebral Cortex Injury and Oxidative Stress, Inflammation, Mitochondrial Dysfunction, and Apoptosis in Sprague Dawley Rats. Antioxidants-Basel. 2024;13(1). doi: ARTN 2

10.3390/antiox13010002. PubMed PMID: WOS:001149407900001.

6. Heymbeeck JAA, de Sousa EB, Lima-Maximino MJr, Pereira A, Maximino C. Nitrergic Signaling Mediation of Neurobehavioral Responses to Stress in Zebrafish. J Neurochem. 2025;169(10). doi: ARTN e70254

10.1111/jnc.70254. PubMed PMID: WOS:001607911600009.

7. Phiel CJ, Wilson CA, Lee VMY, Klein PS. GSK-3α regulates production of Alzheimer's disease amyloid-β peptides. Nature. 2003;423(6938):435-9. doi: 10.1038/nature01640. PubMed PMID: WOS:000183012000041.

8. Conde S, Pérez DI, Martínez A, Perez C, Moreno FJ. Thienyl and phenyl α-halomethyl ketones: New inhibitors of glycogen synthase kinase (GSK-3β) from a library of compound searching. J Med Chem. 2003;46(22):4631-3. doi: 10.1021/jm034108b. PubMed PMID: WOS:000185991100002.

9. Góral I, Wichur T, Slugocka E, Godyn J, Szalaj N, Zareba P, et al. Connecting GSK-3β Inhibitory Activity with IKK-β or ROCK-1 Inhibition to Target Tau Aggregation and Neuroinflammation in Alzheimer's Disease-Discovery, In Vitro and In Cellulo Activity of Thiazole-Based Inhibitors. Molecules. 2024;29(11). doi: ARTN 2616

10.3390/molecules29112616. PubMed PMID: WOS:001245650100001.

10. Llorens-Martín M, Jurado J, Hernádez F, Avila J. GSK-3β, a pivotal kinase in Alzheimer disease. Front Mol Neurosci. 2014;7. doi: ARTN 46

10.3389/fnmol.2014.00046. PubMed PMID: WOS:000347970800001.

11. Hamdan AME, Abu-Elfotuh K, Alshaman R, Salem HA, Hamdan AM, Alaisayi KHA, et al. The protective role of Astaxanthin against the central and peripheral detrimental effects for chronic administration of sweeteners in a rat model: Involvement of multiple signaling axes. J Nutr Biochem. 2026;153. doi: ARTN 110309

10.1016/j.jnutbio.2026.110309. PubMed PMID: WOS:001712551400001.

12. Zhang JX, Huang XY, Ma LC, Chen ZX, Li TY, Wang LJ, et al. Micro-Organ Chip Deciphers Tumor-Derived G-CSF as Remote Commander of Lung Pre-Metastatic Niche via VEGFA-KDR Cascade. Adv Sci. 2026;13(7). doi: 10.1002/advs.202518584. PubMed PMID: WOS:001620062700001.

13. Duan XW, Tan Y, Zhang YK, Wang B, Zhang S, Li T, et al. Restoration of RBM22 overcomes the transcriptional and epigenetic barriers of cardiomyocyte proliferation for heart regeneration. Nat Commun. 2026;17(1). doi: ARTN 3684

10.1038/s41467-026-70235-3. PubMed PMID: WOS:001747237300007.

14. Pat Y, Yazici D, Zeyneloglu C, Babayev H, Ardicli S, Garci-Sanchez A, et al. Cellular Stress, Inflammation and Barrier Damage in Gut Epithelial Cells Caused by Aspartame. Allergy. 2026;81(3):884-901. doi: 10.1111/all.70095. PubMed PMID: WOS:001599539800001.

15. Li NR, Zeng YX, Gu YF, Xie P, Deng BY, Lu SF, et al. Aspartame increases the risk of liver cancer through CASP1 protein: A comprehensive network analysis insights. Ecotox Environ Safe. 2025;294. doi: ARTN 118089

10.1016/j.ecoenv.2025.118089. PubMed PMID: WOS:001456788900001.

16. Fogel MN, Khalil A, Khaled SF, Rodriguez EM, Payne KM, Blount JR, et al. Aspartame and Its Potential Neurocognitive Effects in Humans. Nutr Rev. 2026;84(5):1026-38. doi: 10.1093/nutrit/nuaf103. PubMed PMID: WOS:001521947700001.

17. Gider V, Budak C. A physics-informed graph neural network to approximate docking-based binding affinity for DYRK2 in Alzheimer's drug repurposing. Sci Rep-Uk. 2026;16(1). doi: ARTN 8357

10.1038/s41598-026-35102-7. PubMed PMID: WOS:001714901500012.

18. Zhang TY, Wang T, Yu KY, Huang CR, Bao KY. Aspartame and ischemic stroke: unraveling the molecular link through network toxicology and molecular docking analysis. Sci Rep-Uk. 2025;15(1). doi: ARTN 23871

10.1038/s41598-025-08898-z. PubMed PMID: WOS:001523065300008.

19. Timmerman R, Burm SM, Bajramovic JJ. An Overview of

Methods to Study Microglia. Front Cell Neurosci. 2018;12. doi: ARTN 242

10.3389/fncel.2018.00242. PubMed PMID: WOS:000440828600001.

20. Zhu YL, Guo XM, Li SM, Wu Y, Zhu F, Qin CF, et al. Naringenin ameliorates amyloid-β pathology and neuroinflammation in Alzheimer's disease. Commun Biol. 2024;7(1). doi: ARTN 912

10.1038/s42003-024-06615-6. PubMed PMID: WOS:001278135400002.

21. Das A, Kim SH, Arifuzzaman S, Yoon T, Chai JC, Lee YS, et al. Transcriptome sequencing reveals that LPS-triggered transcriptional responses in established microglia BV2 cell lines are poorly representative of primary microglia. J Neuroinflamm. 2016;13. doi: ARTN 182

10.1186/s12974-016-0644-1. PubMed PMID: WOS:000379327300002.

22. Umpornpun KP, Oldham BB, Vekaria HJ, Zamorano M, Merchant M, Johnson GIII, et al. Primary and immortalized microglia exhibit divergent responses to hemoglobin. Exp Neurol. 2026;403. doi: ARTN 115838

10.1016/j.expneurol.2026.115838. PubMed PMID: WOS:001791624800001.

23. Butovsky O, Weiner HL. Microglial signatures and their role in health and disease. Nat Rev Neurosci. 2018;19(10):622-35. doi: 10.1038/s41583-018-0057-5. PubMed PMID: WOS:000445094700008.

24. Mancuso R, Fattorelli N, Martinez-Muriana A, Davis E, Wolfs L, van den Daele J, et al. Xenografted human microglia display diverse transcriptomic states in response to Alzheimer's disease-related amyloid-β pathology. Nat Neurosci. 2024;27(5):886-900. doi: 10.1038/s41593-024-01600-y. PubMed PMID: WOS:001194682200002.

25. Chang CY, Wu CC, Tzeng CY, Li JR, Chen YF, Chen WY, et al. NMDA receptor blockade attenuates Japanese encephalitis virus infection-induced microglia activation. J Neuroinflamm. 2024;21(1). doi: ARTN 291

10.1186/s12974-024-03288-0. PubMed PMID: WOS:001351597800001.

26. Pearson A, Koprivica M, Eisenbaum M, Ortiz C, Browning M, Vincennie T, et al. PPARγ activation ameliorates cognitive impairment and chronic microglial activation in the aftermath of r-mTBI. J Neuroinflamm. 2024;21(1). doi: ARTN 194

10.1186/s12974-024-03173-w. PubMed PMID: WOS:001284609200001.

27. Bustin SA, Ruijter JM, van den Hoff MJB, Kubista M, Pfaffl MW, Shipley GL, et al. MIQE 2.0: Revision of the Minimum Information for Publication of Quantitative Real-Time PCR Experiments Guidelines. Clin Chem. 2025;71(6):634-51. doi: 10.1093/clinchem/hvaf043. PubMed PMID: WOS:001473412900001.

28. Taylor SC, Nadeau K, Abbasi M, Lachance C, Nguyen M, Fenrich J. The Ultimate qPCR Experiment: Producing Publication Quality, Reproducible Data the First Time. Trends Biotechnol. 2019;37(7):761-74. doi: 10.1016/j.tibtech.2018.12.002. PubMed PMID: WOS:000471155300009.

Attachment Submitted filename: Response to Reviewers.docx

# Editor report: Decision Letter 1

**DOI:** 10.1371/journal.pone.0361766.r003

**Authors:** António Raposo

14 Sep 2026

Dear Dr. Sheng,

Thank you for submitting your manuscript to PLOS One. After careful consideration, we feel that it has merit but does not fully meet PLOS One’s publication criteria as it currently stands. Therefore, we invite you to submit a revised version of the manuscript that addresses the points raised during the review process.

Please submit your revised manuscript by Oct 14 2026 11:59PM. If you will need more time than this to complete your revisions, please reply to this message or contact the journal office at plosone@plos.org. When you're ready to submit your revision, log on to https://www.editorialmanager.com/pone/ and select the 'Submissions Needing Revision' folder to locate your manuscript file.

- A letter that responds to each point raised by the academic editor and reviewer(s). You should upload this letter as a separate file labeled 'Response to Reviewers'.
- A marked-up copy of your manuscript that highlights changes made to the original version. You should upload this as a separate file labeled 'Revised Manuscript with Track Changes'.
- An unmarked version of your revised paper without tracked changes. You should upload this as a separate file labeled 'Manuscript'.

If you would like to make changes to your financial disclosure, please include your updated statement in your cover letter. Guidelines for resubmitting your figure files are available below the reviewer comments at the end of this letter.

If applicable, we recommend that you deposit your laboratory protocols in protocols.io to enhance the reproducibility of your results. Protocols.io assigns your protocol its own identifier (DOI) so that it can be cited independently in the future. For instructions see: https://journals.plos.org/plosone/s/submission-guidelines#loc-laboratory-protocols. Additionally, PLOS One offers an option for publishing peer-reviewed Lab Protocol articles, which describe protocols hosted on protocols.io. Read more information on sharing protocols at https://plos.org/protocols?utm_medium=editorial-email&utm_source=authorletters&utm_campaign=protocols.

As the corresponding author, your ORCID iD is verified in the submission system and will appear in the published article. PLOS supports the use of ORCID, and we encourage all coauthors to register for an ORCID iD and use it as well. Please encourage your coauthors to verify their ORCID iD within the submission system before final acceptance, as unverified ORCID iDs will not appear in the published article. *Only*  the individual author can complete the verification step; PLOS staff *cannot*  verify ORCID iDs on behalf of authors.

We look forward to receiving your revised manuscript.

Kind regards,

António Raposo

Academic Editor

PLOS One

Journal Requirements:

1. If the reviewer comments include a recommendation to cite specific previously published works, please review and evaluate these publications to determine whether they are relevant and should be cited. There is no requirement to cite these works unless the editor has indicated otherwise.

2. Please review your reference list to ensure that it is complete and correct. If you have cited papers that have been retracted, please include the rationale for doing so in the manuscript text, or remove these references and replace them with relevant current references. Any changes to the reference list should be mentioned in the rebuttal letter that accompanies your revised manuscript. If you need to cite a retracted article, indicate the article’s retracted status in the References list and also include a citation and full reference for the retraction notice.

**Additional Editor Comments:**

The revised manuscript is clearly improved, and the authors have addressed most of the concerns raised in the previous round. The additional IBA-1 data, clarification of the aspartame concentration used in the qPCR and ELISA experiments, revision of the molecular docking interpretation, and the expanded discussion of the limitations of the BV2 model have all strengthened the paper.

I still think that a few points need to be corrected before the manuscript can be accepted.

The main issue remains the interpretation of the aspartame concentrations used in vitro. The authors now acknowledge that the tested concentrations are much higher than those expected after normal dietary exposure, which is important. However, some parts of the Discussion still go too far in linking these findings to possible human neurological risk. The arguments concerning chronic exposure, blood-brain barrier dysfunction, metabolite accumulation, or higher local brain exposure are interesting possibilities, but they are not demonstrated by the present study. I would therefore suggest keeping the interpretation more firmly at the level of a mechanistic in vitro model and avoiding implications regarding usual human consumption.

I also remain concerned about the use of 1 mg/mL aspartame for the mechanistic experiments. At this concentration, cell viability was approximately 63%. This is a substantial reduction in viability, and it is difficult to exclude the possibility that cellular stress or cytotoxicity contributed to the increase in inflammatory markers. For this reason, I would avoid describing 1 mg/mL as a “sub-cytotoxic” concentration and would add this point explicitly to the limitations.

The statistical section has improved, particularly with the clarification that Dunnett’s test was used. However, I do not think the statement that the qPCR experiments were “sufficiently powered” is justified when only three biological replicates were used and no formal power analysis was performed. This sentence should be removed or softened, and the exploratory nature of these data should be stated more clearly.

Some of the wording in the Results and Discussion should also be moderated. For example, statements suggesting that the experiments provide “direct experimental evidence” for a role of aspartame in Alzheimer’s disease-associated neuroinflammation are stronger than the model can support. The data show an inflammatory response in an immortalized murine microglial cell line under relatively high in vitro concentrations of aspartame, but they do not directly demonstrate an effect on Alzheimer’s disease pathogenesis.

I also noticed an inconsistency in the revision documents. The cover letter states that corrections for multiple comparisons were included, whereas the response to the statistical comment states that no formal multiplicity correction was applied across endpoints and that the reported p-values are nominal. This should be made consistent before resubmission.

The manuscript is now much closer to being suitable for publication. I do not think additional major experiments are necessary, but the remaining issues concerning translational interpretation, cytotoxicity, statistical caution, and wording should be addressed carefully.

[Note: HTML markup is below. Please do not edit.]

[NOTE: If reviewer comments were submitted as an attachment file, they will be attached to this email and accessible via the submission site. Please log into your account, locate the manuscript record, and check for the action link "View Attachments". If this link does not appear, there are no attachment files.]

To ensure your figures meet our technical requirements, please review our figure guidelines: https://journals.plos.org/plosone/s/figures

You may also use PLOS’s free figure tool, NAAS, to help you prepare publication quality figures: https://journals.plos.org/plosone/s/figures#loc-tools-for-figure-preparation.

NAAS will assess whether your figures meet our technical requirements by comparing each figure against our figure specifications.

# Author comment: Author response to Decision Letter 2

**DOI:** 10.1371/journal.pone.0361766.r004

24 Sep 2026

PONE-D-26-23444R1

Unveiling the Neuroinflammatory Potential of Aspartame in Alzheimer's Disease: A Comprehensive Network Toxicology and Experimental Validation Approach

PLOS One

Dear Dr. Sheng,

Thank you for submitting your manuscript to PLOS One. After careful consideration, we feel that it has merit but does not fully meet PLOS One’s publication criteria as it currently stands. Therefore, we invite you to submit a revised version of the manuscript that addresses the points raised during the review process.

Please submit your revised manuscript by Oct 14 2026 11:59PM. If you will need more time than this to complete your revisions, please reply to this message or contact the journal office at plosone@plos.org. When you're ready to submit your revision, log on to https://www.editorialmanager.com/pone/ and select the 'Submissions Needing Revision' folder to locate your manuscript file.

Please include the following items when submitting your revised manuscript:

• A letter that Responses to each point raised by the academic editor and reviewer(s). You should upload this letter as a separate file labeled 'Response to Reviewers'.

• A marked-up copy of your manuscript that highlights changes made to the original version. You should upload this as a separate file labeled 'Revised Manuscript with Track Changes'.

• An unmarked version of your revised paper without tracked changes. You should upload this as a separate file labeled 'Manuscript'.

If you would like to make changes to your financial disclosure, please include your updated statement in your cover letter. Guidelines for resubmitting your figure files are available below the reviewer comments at the end of this letter.

If applicable, we recommend that you deposit your laboratory protocols in protocols.io to enhance the reproducibility of your results. Protocols.io assigns your protocol its own identifier (DOI) so that it can be cited independently in the future. For instructions see: https://journals.plos.org/plosone/s/submission-guidelines#loc-laboratory-protocols. Additionally, PLOS One offers an option for publishing peer-reviewed Lab Protocol articles, which describe protocols hosted on protocols.io. Read more information on sharing protocols at https://plos.org/protocols?utm_medium=editorial-email&utm_source=authorletters&utm_campaign=protocols.

As the corResponseing author, your ORCID iD is verified in the submission system and will appear in the published article. PLOS supports the use of ORCID, and we encourage all coauthors to register for an ORCID iD and use it as well. Please encourage your coauthors to verify their ORCID iD within the submission system before final acceptance, as unverified ORCID iDs will not appear in the published article. Only the individual author can complete the verification step; PLOS staff cannot verify ORCID iDs on behalf of authors.

We look forward to receiving your revised manuscript.

Kind regards,

António Raposo

Academic Editor

PLOS One

Response: Thank you very much for your email and for giving us the opportunity to revise our manuscript. We are truly grateful for your careful consideration and for the constructive comments provided by you and the reviewer. These comments have been extremely helpful in improving the clarity, caution, and overall quality of our work. We have carefully revised the manuscript in response to all the comments. A detailed point-by-point response to each comment is provided in the separate “Response to Reviewers” file. We have also prepared a marked-up copy of the manuscript with track changes and an unmarked version, as requested.

Journal Requirements:

1. If the reviewer comments include a recommendation to cite specific previously published works, please review and evaluate these publications to determine whether they are relevant and should be cited. There is no requirement to cite these works unless the editor has indicated otherwise.

Response: We have carefully reviewed the reviewer comments for this round and note that the reviewer did not recommend any specific previously published works for citation. We have therefore not added any references on this basis. We sincerely appreciate your reminder, which helps ensure that our reference list remains appropriate and fully compliant with the journal’s policy.

2. Please review your reference list to ensure that it is complete and correct. If you have cited papers that have been retracted, please include the rationale for doing so in the manuscript text, or remove these references and replace them with relevant current references. Any changes to the reference list should be mentioned in the rebuttal letter that accompanies your revised manuscript. If you need to cite a retracted article, indicate the article’s retracted status in the References list and also include a citation and full reference for the retraction notice.

Response: We have thoroughly checked the reference list for completeness and accuracy. We confirm that no retracted articles are cited in our manuscript.

Additional Editor Comments:

(1) The revised manuscript is clearly improved, and the authors have addressed most of the concerns raised in the previous round. The additional IBA-1 data, clarification of the aspartame concentration used in the qPCR and ELISA experiments, revision of the molecular docking interpretation, and the expanded discussion of the limitations of the BV2 model have all strengthened the paper. I still think that a few points need to be corrected before the manuscript can be accepted.

Response: We sincerely thank the Editor for the careful review and for the positive assessment of our revised manuscript. We agree that a few points still require correction before the manuscript can be accepted, and we will address them carefully in the next revision.

(2) The main issue remains the interpretation of the aspartame concentrations used in vitro. The authors now acknowledge that the tested concentrations are much higher than those expected after normal dietary exposure, which is important. However, some parts of the Discussion still go too far in linking these findings to possible human neurological risk. The arguments concerning chronic exposure, blood-brain barrier dysfunction, metabolite accumulation, or higher local brain exposure are interesting possibilities, but they are not demonstrated by the present study. I would therefore suggest keeping the interpretation more firmly at the level of a mechanistic in vitro model and avoiding implications regarding usual human consumption.

Response: We sincerely thank the Editor for this insightful comment. The in vitro concentrations should remain firmly at the level of a mechanistic cell model and should not be extended to imply risks from usual human consumption. In the revised Discussion, we have carefully moderated all statements that could be read as linking our findings to human neurological risk. Specifically, we have replaced phrases such as “experimental evidence for the involvement of aspartame in the pathogenesis of AD” with “generate mechanistic hypotheses regarding the potential involvement of aspartame in AD-related pathways, although further validation is required.” We have also removed the claim that our findings underscore the pro-inflammatory potential of aspartame at “pathophysiologically relevant exposures” and instead state that the concentrations used are much higher than those expected from normal dietary exposure. The statement that aspartame may “synergistically accelerate the pathological progression of AD” has been revised to emphasize that this is a computational prediction requiring experimental validation and should not be interpreted as direct evidence of accelerated AD pathology. Furthermore, we have deleted the sentence implying that long-term aspartame consumption may provoke RNA metabolic disturbances, protein homeostasis imbalance, and cellular stress in the central nervous system, and replaced it with a clear statement that our hypotheses are based on network predictions and high-concentration in vitro experiments and do not demonstrate such effects from normal dietary consumption. We have also added a caveat that the 1 mg/mL concentration reduced cell viability to approximately 63%, so we cannot exclude the possibility that cellular stress or cytotoxicity contributed to the inflammatory responses. Throughout the revised Discussion, we now consistently describe the work as providing initial in vitro evidence in a BV2 cell model and call for future validation using physiologically relevant exposure models and in vivo systems.

We believe these changes fully address the Editor’s concern while preserving the scientific value of our study. The revision is highlighted in red from lines 10-11 on page 7, lines 27-30 on page 19, lines 1on page 20, lines 27-30 on page 21, lines 1-2 on page 22.

(3) I also remain concerned about the use of 1 mg/mL aspartame for the mechanistic experiments. At this concentration, cell viability was approximately 63%. This is a substantial reduction in viability, and it is difficult to exclude the possibility that cellular stress or cytotoxicity contributed to the increase in inflammatory markers. For this reason, I would avoid describing 1 mg/mL as a “sub-cytotoxic” concentration and would add this point explicitly to the limitations.

Response: We sincerely thank the Editor for this important and constructive comment. 1 mg/mL aspartame used for the mechanistic experiments cannot be regarded as sub-cytotoxic, because cell viability was reduced to approximately 63%. We also agree that, at this level of viability reduction, it is difficult to exclude the possibility that cellular stress or cytotoxicity contributed to the increases in inflammatory markers. In the revised manuscript, we have therefore removed the phrase “without excessive cytotoxicity” from the Discussion and replaced it with a clear statement that this concentration reduced cell viability to approximately 63%, that cellular stress or cytotoxicity cannot be excluded as a contributing factor, and that the mechanistic findings at this concentration should be interpreted with this caveat. In addition, we have explicitly added this point to the Limitations section. The revised text now states that 1 mg/mL should not be described as a sub-cytotoxic concentration, that the inflammatory responses observed at this dose may partly reflect a stress response, and that future studies should include a wider range of lower, non-cytotoxic concentrations together with rigorous cytotoxicity controls to better distinguish direct pro-inflammatory effects from secondary responses to cellular damage.

We believe these changes directly address the Editor’s concern and ensure that our conclusions remain appropriately cautious and transparent. The revision is highlighted in red from lines 27-30 on page 21, lines 1-2 on page 22.

(4)The statistical section has improved, particularly with the clarification that Dunnett’s test was used. However, I do not think the statement that the qPCR experiments were “sufficiently powered” is justified when only three biological replicates were used and no formal power analysis was performed. This sentence should be removed or softened, and the exploratory nature of these data should be stated more clearly.

Response: We sincerely thank the Editor for this careful statistical comment. In the revised Statistical Analysis section, we now explicitly state that the qPCR data should be regarded as exploratory and hypothesis-generating rather than confirmatory. These changes make the exploratory nature of the qPCR data clear and ensure that our statistical reporting is appropriately cautious and consistent throughout the manuscript. We are grateful to the Editor for pointing this out. The revision is highlighted in red from lines 6-9 on page 8.

(5) Some of the wording in the Results and Discussion should also be moderated. For example, statements suggesting that the experiments provide “direct experimental evidence” for a role of aspartame in Alzheimer’s disease-associated neuroinflammation are stronger than the model can support. The data show an inflammatory response in an immortalized murine microglial cell line under relatively high in vitro concentrations of aspartame, but they do not directly demonstrate an effect on Alzheimer’s disease pathogenesis.

Response: We sincerely thank you for this careful comment. Our data show an inflammatory response in an immortalized murine microglial cell line under relatively high in vitro concentrations of aspartame, but they do not directly demonstrate an effect on Alzheimer’s disease pathogenesis. We have therefore moderated the relevant wording in the Results and Discussion sections. In the Results, we removed the phrase “direct experimental evidence” and revised the sentence to state that the findings provide “in vitro evidence of microglial inflammatory activation.” In the Discussion, we also softened the description of our experimental data as “the first biological evidence” and now describe them as “initial in vitro evidence” in a BV2 cell model. These changes keep our interpretation firmly at the level of a mechanistic in vitro model and avoid overstating the translational implications of the study. We are grateful to the Editor for this helpful guidance. The revision is highlighted in red from lines 3 on page 15.

(6) I also noticed an inconsistency in the revision documents. The cover letter states that corrections for multiple comparisons were included, whereas the response to the statistical comment states that no formal multiplicity correction was applied across endpoints and that the reported p-values are nominal. This should be made consistent before resubmission.

Response: We sincerely thank you for identifying this inconsistency between the revision documents. We fully agree that the cover letter and the response to the statistical comment should not contradict each other, and we apologize for the confusion caused by the wording in the cover letter.

To clarify the manuscript does not include formal corrections for multiple comparisons. The p-values reported in our study are nominal and were not adjusted for multiplicity across endpoints. The statement in the cover letter referring to “corrections for multiple comparisons” was inaccurate and did not reflect the actual statistical approach used in the study. To ensure full consistency between the manuscript and our response, we have revised the Statistical Analysis section to state explicitly: “No formal correction for multiple comparisons was applied.” This addition makes the manuscript’s statistical declaration unambiguous and fully aligned with our response to the statistical comment. The manuscript now clearly describes the exploratory nature of the data and the absence of formal multiplicity correction, and we have verified that no other section of the manuscript contains a conflicting statement on this point.

We are grateful to the Editor for catching this inconsistency and for giving us the opportunity to correct it before resubmission. The revision is highlighted in red.

(7) The manuscript is now much closer to being suitable for publication. I do not think additional major experiments are necessary, but the remaining issues concerning translational interpretation, cytotoxicity, statistical caution, and wording should be addressed carefully.

Response: We are very grateful for your encouraging assessment and for confirming that no additional major experiments are needed. We also appreciate your guidance on the remaining issues, which we have taken seriously. Following your suggestion, we went through the entire manuscript once more and made a number of careful adjustments to ensure that our interpretation, statistical reporting, and wording are all appropriately cautious and consistent.

For translational interpretation, we softened several statements that could have been read as going beyond what our data can support. For instance, the Introduction now says that we seek to “explore the potential mechanisms linking aspartame to AD” instead of “uncover the potential pathological mechanisms.” In the Results, the molecular docking section no longer describes all targets as showing “stable binding interactions”; it now states that the targets display “varying binding affinities, with GSK3β and KDR showing the strongest interactions and VEGFA exhibiting weaker binding.” We also removed the word “sustained” from the NO response description, since our experiment did not measure long-term effects. For cytotoxicity, we have made it explicit in both the Discussion and the Limitations that the 1 mg/mL concentration reduced BV2 viability to approximately 63%, that cellular stress or cytotoxicity may have contributed to the inflammatory readouts, and that this concentration should not be called sub-cytotoxic. We also suggest that future studies test lower, non-cytotoxic concentrations with proper controls. For statistical caution, we confirmed that the Statistical Analysis section now clearly states that no formal correction for multiple comparisons was applied, that the reported p-values are nominal, and that the qPCR data are exploratory and hypothesis-generating given the three biological replicates and the absence of a formal power analysis. Finally, we removed the remaining overstatement in the Conclusion regarding “the potential risks of long-term aspartame consumption for individuals at high risk of AD.” The Conclusion now says that the study provides a basis for further investigation and highlights the need for future studies using physiologically relevant exposure models.

We believe these changes make the manuscript’s conclusions fully supported by the data and appropriately cautious. Thank you again for your helpful guidance. The revision is highlighted in red.

Attachment Submitted filename: Response_to_Reviewers_auresp_2.docx

# Editor report: Decision Letter 2

**DOI:** 10.1371/journal.pone.0361766.r005

**Authors:** António Raposo

28 Sep 2026

Dear Dr. Sheng,

Thank you for submitting your manuscript to PLOS One. After careful consideration, we feel that it has merit but does not fully meet PLOS One’s publication criteria as it currently stands. Therefore, we invite you to submit a revised version of the manuscript that addresses the points raised during the review process.

Please submit your revised manuscript by Nov 12 2026 11:59PM. If you will need more time than this to complete your revisions, please reply to this message or contact the journal office at plosone@plos.org. When you're ready to submit your revision, log on to https://www.editorialmanager.com/pone/ and select the 'Submissions Needing Revision' folder to locate your manuscript file.

- A letter that responds to each point raised by the academic editor and reviewer(s). You should upload this letter as a separate file labeled 'Response to Reviewers'.
- A marked-up copy of your manuscript that highlights changes made to the original version. You should upload this as a separate file labeled 'Revised Manuscript with Track Changes'.
- An unmarked version of your revised paper without tracked changes. You should upload this as a separate file labeled 'Manuscript'.

If you would like to make changes to your financial disclosure, please include your updated statement in your cover letter. Guidelines for resubmitting your figure files are available below the reviewer comments at the end of this letter.

If applicable, we recommend that you deposit your laboratory protocols in protocols.io to enhance the reproducibility of your results. Protocols.io assigns your protocol its own identifier (DOI) so that it can be cited independently in the future. For instructions see: https://journals.plos.org/plosone/s/submission-guidelines#loc-laboratory-protocols. Additionally, PLOS One offers an option for publishing peer-reviewed Lab Protocol articles, which describe protocols hosted on protocols.io. Read more information on sharing protocols at https://plos.org/protocols?utm_medium=editorial-email&utm_source=authorletters&utm_campaign=protocols.

As the corresponding author, your ORCID iD is verified in the submission system and will appear in the published article. PLOS supports the use of ORCID, and we encourage all coauthors to register for an ORCID iD and use it as well. Please encourage your coauthors to verify their ORCID iD within the submission system before final acceptance, as unverified ORCID iDs will not appear in the published article. *Only*  the individual author can complete the verification step; PLOS staff *cannot*  verify ORCID iDs on behalf of authors.

We look forward to receiving your revised manuscript.

Kind regards,

António Raposo

Academic Editor

PLOS One

Journal Requirements:

If the reviewer comments include a recommendation to cite specific previously published works, please review and evaluate these publications to determine whether they are relevant and should be cited. There is no requirement to cite these works unless the editor has indicated otherwise.

Please review your reference list to ensure that it is complete and correct. If you have cited papers that have been retracted, please include the rationale for doing so in the manuscript text, or remove these references and replace them with relevant current references. Any changes to the reference list should be mentioned in the rebuttal letter that accompanies your revised manuscript. If you need to cite a retracted article, indicate the article’s retracted status in the References list and also include a citation and full reference for the retraction notice.

Additional Editor Comments:

Thank you for taking the time to revise the manuscript again. I have read the revised version together with your responses to my previous comments, and I appreciate the care you have taken in addressing the concerns that remained after the last round.

I am particularly pleased to see that the interpretation of the experimental findings is now much more cautious. The limitations associated with the relatively high aspartame concentrations are clearer, and the manuscript now openly acknowledges that the reduced cell viability at 1 mg/mL makes it difficult to rule out a contribution from cellular stress or cytotoxicity to the inflammatory response. I also think the changes to the statistical section are helpful, especially the clarification that the qPCR findings should be considered exploratory rather than confirmatory. These changes have addressed my main concerns from the previous round.

There are just a few points that I think still need some attention.

The first concerns the Abstract. Although the Discussion and Conclusions are now appropriately cautious, a couple of statements in the Abstract still seem somewhat stronger than the data allow. In particular, saying that aspartame “may induce microglial neuroinflammation through multiple signaling pathways” and that the study provides “critical insights into the neurological safety of aspartame” could still be read as extending the findings beyond this particular in vitro model. I would suggest bringing these sentences into line with the more measured language now used elsewhere in the manuscript.

Along the same lines, I would avoid saying that molecular docking “validated” favorable interactions. Docking provides a prediction of possible binding interactions rather than experimental validation, so a term such as “predicted” or “suggested” would be more accurate.

I also noticed that the Results still refer to “pathways significantly associated with aspartame-induced AD.” I think this should be changed. The study does not show that aspartame induces AD, and this wording does not really reflect the more careful interpretation that the authors have adopted in this revision. Referring instead to pathways enriched among the overlapping aspartame- and AD-associated targets would avoid this problem.

There are also two small technical points that would be worth checking. The Methods describe a STRING interaction score of 0.9 as “medium confidence,” which does not seem to be the correct description for such a stringent threshold. In addition, the PPI network is reported as having 143 nodes and 231 edges, followed by “degree of 23.464.” It is not clear to me what this value represents, so I would ask the authors to check the calculation and specify the network metric being reported.

Finally, there are still a few grammatical and typographical issues throughout the text. Nothing here affects my assessment of the work, but another careful read through the manuscript would be worthwhile before publication.

I appreciate the way the authors have responded to the comments throughout the revision process. The main scientific concerns I raised previously have, in my view, been addressed, and I do not see a need for further experiments at this stage. The remaining points are relatively minor and should be straightforward to correct.

[Note: HTML markup is below. Please do not edit.]

[NOTE: If reviewer comments were submitted as an attachment file, they will be attached to this email and accessible via the submission site. Please log into your account, locate the manuscript record, and check for the action link "View Attachments". If this link does not appear, there are no attachment files.]

To ensure your figures meet our technical requirements, please review our figure guidelines: https://journals.plos.org/plosone/s/figures

You may also use PLOS’s free figure tool, NAAS, to help you prepare publication quality figures: https://journals.plos.org/plosone/s/figures#loc-tools-for-figure-preparation.

NAAS will assess whether your figures meet our technical requirements by comparing each figure against our figure specifications.

# Author comment: Author response to Decision Letter 3

**DOI:** 10.1371/journal.pone.0361766.r006

28 Sep 2026

PONE-D-26-23444R2

Unveiling the Neuroinflammatory Potential of Aspartame in Alzheimer's Disease: A Comprehensive Network Toxicology and Experimental Validation Approach

PLOS One

Dear Dr. Sheng,

Thank you for submitting your manuscript to PLOS One. After careful consideration, we feel that it has merit but does not fully meet PLOS One’s publication criteria as it currently stands. Therefore, we invite you to submit a revised version of the manuscript that addresses the points raised during the review process.

Please submit your revised manuscript by Nov 12 2026 11:59PM. If you will need more time than this to complete your revisions, please reply to this message or contact the journal office at plosone@plos.org. When you're ready to submit your revision, log on to https://www.editorialmanager.com/pone/ and select the 'Submissions Needing Revision' folder to locate your manuscript file.

Please include the following items when submitting your revised manuscript:

A letter that responds to each point raised by the academic editor and reviewer(s). You should upload this letter as a separate file labeled 'Response to Reviewers'.

A marked-up copy of your manuscript that highlights changes made to the original version. You should upload this as a separate file labeled 'Revised Manuscript with Track Changes'.

An unmarked version of your revised paper without tracked changes. You should upload this as a separate file labeled 'Manuscript'.

If you would like to make changes to your financial disclosure, please include your updated statement in your cover letter. Guidelines for resubmitting your figure files are available below the reviewer comments at the end of this letter.

If applicable, we recommend that you deposit your laboratory protocols in protocols.io to enhance the reproducibility of your results. Protocols.io assigns your protocol its own identifier (DOI) so that it can be cited independently in the future. For instructions see: https://journals.plos.org/plosone/s/submission-guidelines#loc-laboratory-protocols. Additionally, PLOS One offers an option for publishing peer-reviewed Lab Protocol articles, which describe protocols hosted on protocols.io. Read more information on sharing protocols at https://plos.org/protocols?utm_medium=editorial-email&utm_source=authorletters&utm_campaign=protocols.

As the corresponding author, your ORCID iD is verified in the submission system and will appear in the published article. PLOS supports the use of ORCID, and we encourage all coauthors to register for an ORCID iD and use it as well. Please encourage your coauthors to verify their ORCID iD within the submission system before final acceptance, as unverified ORCID iDs will not appear in the published article. Only the individual author can complete the verification step; PLOS staff cannot verify ORCID iDs on behalf of authors.

We look forward to receiving your revised manuscript.

Kind regards,

António Raposo

Academic Editor

PLOS One

Additional Editor Comments:

Thank you for taking the time to revise the manuscript again. I have read the revised version together with your responses to my previous comments, and I appreciate the care you have taken in addressing the concerns that remained after the last round.

I am particularly pleased to see that the interpretation of the experimental findings is now much more cautious. The limitations associated with the relatively high aspartame concentrations are clearer, and the manuscript now openly acknowledges that the reduced cell viability at 1 mg/mL makes it difficult to rule out a contribution from cellular stress or cytotoxicity to the inflammatory response. I also think the changes to the statistical section are helpful, especially the clarification that the qPCR findings should be considered exploratory rather than confirmatory. These changes have addressed my main concerns from the previous round.

Response: We sincerely thank you for your encouraging comments and for your careful assessment of our revised manuscript. We are delighted to hear that you are pleased with the more cautious interpretation of the experimental findings and that the main scientific concerns from the previous round have been addressed. We also appreciate your confirmation that no further experiments are required at this stage. We have carefully revised the manuscript according to the remaining points, and we provide a point-by-point response below. All changes have been highlighted in the revised manuscript.

There are just a few points that I think still need some attention.

(1) The first concerns the Abstract. Although the Discussion and Conclusions are now appropriately cautious, a couple of statements in the Abstract still seem somewhat stronger than the data allow. In particular, saying that aspartame “may induce microglial neuroinflammation through multiple signaling pathways” and that the study provides “critical insights into the neurological safety of aspartame” could still be read as extending the findings beyond this particular in vitro model. I would suggest bringing these sentences into line with the more measured language now used elsewhere in the manuscript.

Response: We sincerely thank the Editor for this thoughtful comment. We fully agree that the Abstract should use the same measured language as the Discussion and Conclusions, and we appreciate the Editor pointing out these two statements. We have revised the Abstract accordingly to avoid extending our findings beyond the in vitro model.

In the revised Abstract, the sentence now clearly limits the conclusion to BV2 microglial cells and explicitly states that the relevance to human neurological safety remains to be established. The sentence replaces “critical insights” with “a basis for further investigation,” which is consistent with the cautious tone used throughout the revised manuscript. We believe these changes fully address the Editor’s concern while preserving the scientific message of the study. The revision is highlighted in red from lines 1-4 on page 2.

(2) Along the same lines, I would avoid saying that molecular docking “validated” favorable interactions. Docking provides a prediction of possible binding interactions rather than experimental validation, so a term such as “predicted” or “suggested” would be more accurate.

Response: We sincerely thank the Editor for this helpful comment. The molecular docking provides a prediction of possible binding interactions rather than experimental validation, and we appreciate the suggestion to use more accurate wording. We have therefore replaced “validated” with “predicted” in the Abstract, so that the description of the docking results is consistent throughout the paper. This change ensure that the docking results are described as computational predictions rather than experimental validation, in line with the Editor’s comment. The revision is highlighted in red from line 23 on page 1.

(3) I also noticed that the Results still refer to “pathways significantly associated with aspartame-induced AD.” I think this should be changed. The study does not show that aspartame induces AD, and this wording does not really reflect the more careful interpretation that the authors have adopted in this revision. Referring instead to pathways enriched among the overlapping aspartame- and AD-associated targets would avoid this problem.

Response: We sincerely thank the Editor for this careful observation. We agree that the phrase “pathways significantly associated with aspartame-induced AD” is inaccurate, because our study does not demonstrate that aspartame induces AD. We have therefore revised this sentence to refer to pathways enriched among the overlapping aspartame- and AD-associated targets, which more accurately reflects the exploratory nature of our analysis. We are grateful to the Editor for helping us align the terminology with the more cautious interpretation adopted in this revision. The revision is highlighted in red from lines 15-16 on page 10.

(4) There are also two small technical points that would be worth checking. The Methods describe a STRING interaction score of 0.9 as “medium confidence,” which does not seem to be the correct description for such a stringent threshold. In addition, the PPI network is reported as having 143 nodes and 231 edges, followed by “degree of 23.464.” It is not clear to me what this value represents, so I would ask the authors to check the calculation and specify the network metric being reported.

Response: We sincerely thank the Editor for these two careful technical observations. We apologize for the inaccurate description. In the STRING database, the confidence score levels are defined as follows: 0.15 (low confidence), 0.40 (medium confidence), 0.70 (high confidence), and 0.90 (highest confidence). Because we applied a minimum interaction score of 0.9, the correct description is “highest confidence,” not “medium confidence.” We have corrected this in the Methods section.

We also thank the Editor for asking us to clarify this value. The network contains 143 nodes and 231 edges. The quantity we originally reported as “degree of 23.464” was not the average degree. The average degree of an undirected network is defined as: ⟨k⟩ = 2L / N, where L is the number of edges and N is the number of nodes. For our network, this gives: ⟨k⟩ = 2 × 231 / 143 ≈ 3.23. This means that, on average, each node in the PPI network is connected to approximately 3.23 other nodes. We have corrected the Results section to report the correct metric and have explicitly named it as the average degree, so that its meaning is unambiguous. The revision is highlighted in red from lines 12-13 on page 5, lines 4-5 on page 9.

We are grateful to the Editor for catching these errors. We have carefully verified all other numerical values and network metrics in the manuscript to ensure that no similar inconsistencies remain.

(5) Finally, there are still a few grammatical and typographical issues throughout the text. Nothing here affects my assessment of the work, but another careful read through the manuscript would be worthwhile before publication.

Response: We sincerely thank the reviewer for this careful reading. We have performed another thorough read-through of the entire manuscript and corrected the grammatical and typographical issues throughout the text. All language-related corrections have been highlighted in red in the revised manuscript for easy identification. These changes are limited to grammar, wording, and typographical formatting and do not alter the scientific content, data interpretation, or conclusions of the study. We hope the revised manuscript is now more polished and suitable for publication.

I appreciate the way the authors have responded to the comments throughout the revision process. The main scientific concerns I raised previously have, in my view, been addressed, and I do not see a need for further experiments at this stage. The remaining points are relatively minor and should be straightforward to correct.

Response: We sincerely thank the reviewer for the positive and encouraging comments. We are grateful that the main scientific concerns raised in the previous round have been addressed and that no further experiments are deemed necessary at this stage. We have carefully addressed all remaining minor points, including the grammatical and typographical issues, and all corresponding revisions have been highlighted in red in the revised manuscript. We hope the revised manuscript is now acceptable for publication.

Attachment Submitted filename: Response_to_Reviewers_auresp_3.docx

# Editor report: Decision Letter 3

**DOI:** 10.1371/journal.pone.0361766.r007

**Authors:** António Raposo

30 Sep 2026

Dear Dr. Sheng,

Thank you for submitting your manuscript to PLOS One. After careful consideration, we feel that it has merit but does not fully meet PLOS One’s publication criteria as it currently stands. Therefore, we invite you to submit a revised version of the manuscript that addresses the points raised during the review process.

Please submit your revised manuscript by Oct 30 2026 11:59PM. If you will need more time than this to complete your revisions, please reply to this message or contact the journal office at plosone@plos.org. When you're ready to submit your revision, log on to https://www.editorialmanager.com/pone/ and select the 'Submissions Needing Revision' folder to locate your manuscript file.

- A letter that responds to each point raised by the academic editor and reviewer(s). You should upload this letter as a separate file labeled 'Response to Reviewers'.
- A marked-up copy of your manuscript that highlights changes made to the original version. You should upload this as a separate file labeled 'Revised Manuscript with Track Changes'.
- An unmarked version of your revised paper without tracked changes. You should upload this as a separate file labeled 'Manuscript'.

If you would like to make changes to your financial disclosure, please include your updated statement in your cover letter. Guidelines for resubmitting your figure files are available below the reviewer comments at the end of this letter.

If applicable, we recommend that you deposit your laboratory protocols in protocols.io to enhance the reproducibility of your results. Protocols.io assigns your protocol its own identifier (DOI) so that it can be cited independently in the future. For instructions see: https://journals.plos.org/plosone/s/submission-guidelines#loc-laboratory-protocols. Additionally, PLOS One offers an option for publishing peer-reviewed Lab Protocol articles, which describe protocols hosted on protocols.io. Read more information on sharing protocols at https://plos.org/protocols?utm_medium=editorial-email&utm_source=authorletters&utm_campaign=protocols.

As the corresponding author, your ORCID iD is verified in the submission system and will appear in the published article. PLOS supports the use of ORCID, and we encourage all coauthors to register for an ORCID iD and use it as well. Please encourage your coauthors to verify their ORCID iD within the submission system before final acceptance, as unverified ORCID iDs will not appear in the published article. *Only*  the individual author can complete the verification step; PLOS staff *cannot*  verify ORCID iDs on behalf of authors.

We look forward to receiving your revised manuscript.

Kind regards,

António Raposo

Academic Editor

PLOS One

**Journal Requirements:**

1. If the reviewer comments include a recommendation to cite specific previously published works, please review and evaluate these publications to determine whether they are relevant and should be cited. There is no requirement to cite these works unless the editor has indicated otherwise.

2. Please review your reference list to ensure that it is complete and correct. If you have cited papers that have been retracted, please include the rationale for doing so in the manuscript text, or remove these references and replace them with relevant current references. Any changes to the reference list should be mentioned in the rebuttal letter that accompanies your revised manuscript. If you need to cite a retracted article, indicate the article’s retracted status in the References list and also include a citation and full reference for the retraction notice.

**Additional Editor Comments:**

Thank you for taking the time to revise the manuscript again. I have read the new version alongside your responses, and the changes have addressed most of the points raised in my previous assessment. The Abstract is more cautious, and the corrections to the STRING confidence threshold and average network degree are clear.

A few passages still need attention before the manuscript can be accepted.

First, some of the wording corrected in the Abstract and Results remains elsewhere in the paper. The Introduction still says that molecular docking was used to “validate the binding affinities” (page 3, lines 25–27), and the Methods still refer to “aspartame-induced AD” (page 5, line 16). Please revise these passages too. Docking predicts possible interactions, and the present study does not show that aspartame induces Alzheimer’s disease.

The point I would particularly like you to check concerns GSK-3β protein expression. The Discussion refers to its experimental assessment on page 19, lines 25–26, and later states that GSK-3β and downstream cytokines were upregulated at both protein and mRNA levels (page 20, lines 19–20). I could find the qPCR results for GSK-3β, but I could not find a corresponding protein assay or protein expression results. If only GSK-3β mRNA was measured, please state this consistently and remove the references to its protein expression. The cytokine measurements by ELISA can be described separately.

This also affects a couple of mechanistic statements. The phrases “robust elucidation” of the NO/oxidative stress–GSK-3β/NF-κB axis (page 20, lines 1–3) and “GSK-3β activation […] we observed” (page 21, line 24) go beyond the measurements presented. An increase in GSK-3β mRNA does not establish activation of the protein. Please describe the proposed pathway as a possible explanation for the findings, rather than a mechanism demonstrated by this study.

Finally, please give the text and figure legends another careful read, particularly the punctuation around the Figure 3 citation and the significance symbols and thresholds in the Figure 8 legend.

[Note: HTML markup is below. Please do not edit.]

[NOTE: If reviewer comments were submitted as an attachment file, they will be attached to this email and accessible via the submission site. Please log into your account, locate the manuscript record, and check for the action link "View Attachments". If this link does not appear, there are no attachment files.]

To ensure your figures meet our technical requirements, please review our figure guidelines: https://journals.plos.org/plosone/s/figures

You may also use PLOS’s free figure tool, NAAS, to help you prepare publication quality figures: https://journals.plos.org/plosone/s/figures#loc-tools-for-figure-preparation.

NAAS will assess whether your figures meet our technical requirements by comparing each figure against our figure specifications.

# Author comment: Author response to Decision Letter 4

**DOI:** 10.1371/journal.pone.0361766.r008

8 Oct 2026

PONE-D-26-23444R3

Unveiling the Neuroinflammatory Potential of Aspartame in Alzheimer's Disease: A Comprehensive Network Toxicology and Experimental Validation Approach

PLOS One

Dear Dr. Sheng,

Thank you for submitting your manuscript to PLOS One. After careful consideration, we feel that it has merit but does not fully meet PLOS One’s publication criteria as it currently stands. Therefore, we invite you to submit a revised version of the manuscript that addresses the points raised during the review process.

Please submit your revised manuscript by Oct 30 2026 11:59PM. If you will need more time than this to complete your revisions, please reply to this message or contact the journal office at plosone@plos.org. When you're ready to submit your revision, log on to https://www.editorialmanager.com/pone/ and select the 'Submissions Needing Revision' folder to locate your manuscript file.

Please include the following items when submitting your revised manuscript:

A letter that responds to each point raised by the academic editor and reviewer(s). You should upload this letter as a separate file labeled 'Response to Reviewers'.

A marked-up copy of your manuscript that highlights changes made to the original version. You should upload this as a separate file labeled 'Revised Manuscript with Track Changes'.

An unmarked version of your revised paper without tracked changes. You should upload this as a separate file labeled 'Manuscript'.

If you would like to make changes to your financial disclosure, please include your updated statement in your cover letter. Guidelines for resubmitting your figure files are available below the reviewer comments at the end of this letter.

If applicable, we recommend that you deposit your laboratory protocols in protocols.io to enhance the reproducibility of your results. Protocols.io assigns your protocol its own identifier (DOI) so that it can be cited independently in the future. For instructions see: https://journals.plos.org/plosone/s/submission-guidelines#loc-laboratory-protocols. Additionally, PLOS One offers an option for publishing peer-reviewed Lab Protocol articles, which describe protocols hosted on protocols.io. Read more information on sharing protocols at https://plos.org/protocols?utm_medium=editorial-email&utm_source=authorletters&utm_campaign=protocols.

As the corresponding author, your ORCID iD is verified in the submission system and will appear in the published article. PLOS supports the use of ORCID, and we encourage all coauthors to register for an ORCID iD and use it as well. Please encourage your coauthors to verify their ORCID iD within the submission system before final acceptance, as unverified ORCID iDs will not appear in the published article. Only the individual author can complete the verification step; PLOS staff cannot verify ORCID iDs on behalf of authors.

We look forward to receiving your revised manuscript.

Kind regards,

António Raposo

Academic Editor

PLOS One

Journal Requirements:

1. If the reviewer comments include a recommendation to cite specific previously published works, please review and evaluate these publications to determine whether they are relevant and should be cited. There is no requirement to cite these works unless the editor has indicated otherwise.

2. Please review your reference list to ensure that it is complete and correct. If you have cited papers that have been retracted, please include the rationale for doing so in the manuscript text, or remove these references and replace them with relevant current references. Any changes to the reference list should be mentioned in the rebuttal letter that accompanies your revised manuscript. If you need to cite a retracted article, indicate the article’s retracted status in the References list and also include a citation and full reference for the retraction notice.

Additional Editor Comments:

Thank you for taking the time to revise the manuscript again. I have read the new version alongside your responses, and the changes have addressed most of the points raised in my previous assessment. The Abstract is more cautious, and the corrections to the STRING confidence threshold and average network degree are clear.

A few passages still need attention before the manuscript can be accepted.

(1) First, some of the wording corrected in the Abstract and Results remains elsewhere in the paper. The Introduction still says that molecular docking was used to “validate the binding affinities” (page 3, lines 25–27), and the Methods still refer to “aspartame-induced AD” (page 5, line 16). Please revise these passages too. Docking predicts possible interactions, and the present study does not show that aspartame induces Alzheimer’s disease.

Response: We sincerely thank the Editor for pointing this out. The wording in the Introduction and Methods should be consistent with the more careful language used elsewhere in the revised manuscript. Molecular docking can only predict possible binding interactions rather than experimentally validate them, and our study does not demonstrate that aspartame induces Alzheimer’s disease. We have therefore revised both passages accordingly. In the Introduction, we changed the sentence “Molecular docking simulations were subsequently performed to validate the binding affinities between aspartame and its targets” to “Molecular docking simulations were subsequently performed to predict the possible binding interactions between aspartame and its targets.” In the Methods, we changed the phrase “potential targets involved in aspartame-induced AD” to “potential targets overlapping between aspartame and AD,” which more accurately reflects that these targets are shared between the two datasets rather than evidence that aspartame causes AD. These changes are fully consistent with the revised Abstract, Results, and Discussion. The modified text has been highlighted in red from line 26 on page 3 and lines 16-17 on page 5 for your convenience. We are grateful to the Editor for helping us eliminate these remaining inconsistencies.

(2) The point I would particularly like you to check concerns GSK-3β protein expression. The Discussion refers to its experimental assessment on page 19, lines 25–26, and later states that GSK-3β and downstream cytokines were upregulated at both protein and mRNA levels (page 20, lines 19–20). I could find the qPCR results for GSK-3β, but I could not find a corresponding protein assay or protein expression results. If only GSK-3β mRNA was measured, please state this consistently and remove the references to its protein expression. The cytokine measurements by ELISA can be described separately.

Response: We sincerely thank the Editor for this careful and important observation. GSK-3β protein expression was not measured; only its mRNA level was assessed. After re-checking the relevant passages, we confirm that we did not perform any protein assay for GSK-3β. The only protein-level measurements in our study were the ELISA-based quantification of secreted cytokines (TNF-α, IL-1β, and IL-6), which are separate from GSK-3β.

We have therefore revised the Discussion to state consistently that GSK-3β was assessed only at the mRNA level. Specifically, in the paragraph comparing our work with Ge et al., the sentence “showing that aspartame upregulates GSK-3β and downstream cytokines at both the protein and mRNA levels” has been changed to “showing that aspartame upregulates GSK-3β mRNA and increases the secretion and mRNA expression of downstream cytokines (TNF-α, IL-1β, and IL-6).” In the Limitations section, the phrase “The GSK-3β activation and pro-inflammatory response we observed” has been changed to “The GSK-3β mRNA upregulation and pro-inflammatory response we observed.” We have also checked the rest of the manuscript and removed any other wording that could be read as referring to GSK-3β protein expression or activation. The cytokine measurements by ELISA are now described separately and clearly, without linking them to GSK-3β protein. All modified text has been highlighted in red from line 19-20 on page 20, and 24 on page 21 in the revised manuscript. We are grateful to the Editor for catching this inconsistency, which has helped us make the manuscript more accurate and transparent.

(3) This also affects a couple of mechanistic statements. The phrases “robust elucidation” of the NO/oxidative stress–GSK-3β/NF-κB axis (page 20, lines 1–3) and “GSK-3β activation […] we observed” (page 21, line 24) go beyond the measurements presented. An increase in GSK-3β mRNA does not establish activation of the protein. Please describe the proposed pathway as a possible explanation for the findings, rather than a mechanism demonstrated by this study.

Response: We sincerely thank the Editor for this thoughtful comment. We agree that an increase in GSK-3β mRNA does not establish activation of the protein, and we should not have used language implying that the NO/oxidative stress–GSK-3β/NF-κB axis was definitively elucidated or that GSK-3β activation was observed. We have therefore revised these mechanistic statements to present the proposed pathway as a possible explanation rather than a demonstrated mechanism. In the Discussion, the sentence “This targeted focus enabled robust elucidation of the NO/oxidative stress-GSK-3β/NF-κB cytokine signaling axis” has been changed to “This targeted focus allowed us to explore a possible involvement of the NO/oxidative stress–GSK-3β/NF-κB cytokine signaling axis.” Similarly, the phrase “The GSK-3β activation and pro-inflammatory response we observed” has been changed to “The GSK-3β mRNA upregulation and pro-inflammatory response we observed.” We have also checked the rest of the manuscript for any other wording that could be read as claiming GSK-3β activation and have revised it accordingly. All modified text has been highlighted in red from lines 1- 2, page 20. We are grateful to the Editor for helping us keep our mechanistic interpretation strictly within the limits of our data.

(4) Finally, please give the text and figure legends another careful read, particularly the punctuation around the Figure 3 citation and the significance symbols and thresholds in the Figure 8 legend.

Response: We sincerely thank the Editor for this final careful reminder. We have now given the text and all figure legends another thorough read, with particular attention to the punctuation around the Figure 3 citation and to the significance symbols and thresholds in the Figure 8 legend. In the Results, the Figure 3 citation previously read “... overlapping aspartame- and AD-associated targets. (Fig 3). The 10 most enriched pathways...”, which was awkwardly punctuated and placed the citation after a full stop. We have corrected this to “... overlapping aspartame- and AD-associated targets (Fig 3). The 10 most enriched pathways...”, so that the citation now sits naturally before the closing period. For Figure 8, we have carefully revised the entire legend; the significance symbols and thresholds have been revised to “**P < 0.01, ****P < 0.0001”. We have also checked the other figure legends for similar issues and have made the necessary corrections. All modified text has been highlighted in red in the revised manuscript for your convenience. We are grateful to the Editor for encouraging this final careful read, which has helped us improve the accuracy and professionalism of the manuscript.

Attachment Submitted filename: Response_to_Reviewers_auresp_4.docx

# Editor report: Decision Letter 4

**DOI:** 10.1371/journal.pone.0361766.r009

**Authors:** António Raposo

9 Oct 2026

Dear Dr. Sheng,

Please submit your revised manuscript by Nov 08 2026 11:59PM. If you will need more time than this to complete your revisions, please reply to this message or contact the journal office at plosone@plos.org. When you're ready to submit your revision, log on to https://www.editorialmanager.com/pone/ and select the 'Submissions Needing Revision' folder to locate your manuscript file.

- A letter that responds to each point raised by the academic editor and reviewer(s). You should upload this letter as a separate file labeled 'Response to Reviewers'.
- A marked-up copy of your manuscript that highlights changes made to the original version. You should upload this as a separate file labeled 'Revised Manuscript with Track Changes'.
- An unmarked version of your revised paper without tracked changes. You should upload this as a separate file labeled 'Manuscript'.

If you would like to make changes to your financial disclosure, please include your updated statement in your cover letter. Guidelines for resubmitting your figure files are available below the reviewer comments at the end of this letter.

If applicable, we recommend that you deposit your laboratory protocols in protocols.io to enhance the reproducibility of your results. Protocols.io assigns your protocol its own identifier (DOI) so that it can be cited independently in the future. For instructions see: https://journals.plos.org/plosone/s/submission-guidelines#loc-laboratory-protocols. Additionally, PLOS One offers an option for publishing peer-reviewed Lab Protocol articles, which describe protocols hosted on protocols.io. Read more information on sharing protocols at https://plos.org/protocols?utm_medium=editorial-email&utm_source=authorletters&utm_campaign=protocols.

As the corresponding author, your ORCID iD is verified in the submission system and will appear in the published article. PLOS supports the use of ORCID, and we encourage all coauthors to register for an ORCID iD and use it as well. Please encourage your coauthors to verify their ORCID iD within the submission system before final acceptance, as unverified ORCID iDs will not appear in the published article. *Only*  the individual author can complete the verification step; PLOS staff *cannot*  verify ORCID iDs on behalf of authors.

We look forward to receiving your revised manuscript.

Kind regards,

António Raposo

Academic Editor

PLOS One

Journal Requirements:

If the reviewer comments include a recommendation to cite specific previously published works, please review and evaluate these publications to determine whether they are relevant and should be cited. There is no requirement to cite these works unless the editor has indicated otherwise.

Please review your reference list to ensure that it is complete and correct. If you have cited papers that have been retracted, please include the rationale for doing so in the manuscript text, or remove these references and replace them with relevant current references. Any changes to the reference list should be mentioned in the rebuttal letter that accompanies your revised manuscript. If you need to cite a retracted article, indicate the article’s retracted status in the References list and also include a citation and full reference for the retraction notice.

Additional Editor Comments:

There are still a few passages that need attention before I can accept the manuscript.

The main remaining issue is in the Discussion (page 20, lines 19–26). Although the wording about protein expression has been corrected, this paragraph still refers to “functional confirmation,” “downstream cytokines,” a “shared neuroinflammatory mechanism,” and effects occurring “through a GSK-3β-associated pathway.” These expressions go beyond what was measured. The experiments show an increase in GSK-3β mRNA alongside changes in inflammatory markers, but they do not establish that GSK-3β mediates those changes. Similarly, the resemblance to the LPS response does not demonstrate that the two treatments act through the same mechanism. Please revise this paragraph to present GSK-3β involvement as a possible explanation that requires further testing.

The same point applies to the phrase “provide a valid proof-of-concept for a mechanistic pathway” in the Limitations section (page 21, lines 25–27). Please describe this more cautiously as preliminary evidence of an inflammatory response accompanied by increased GSK-3β mRNA expression. The Conclusion should follow the same approach: “systematically elucidate” and “through a multi-target network in vitro” still suggest that the proposed mechanisms have been established experimentally.

Please also check the molecular docking Results (page 12, lines 11–16). The sentence beginning “Our results demonstrated that aspartame forms…” should describe predicted interactions, and the discussion of binding affinities should make clear that these are computational estimates.

Finally, there is an incomplete sentence in the functional enrichment Methods (page 5, lines 19–21), beginning “Key pathways overlapping aspartame- and AD-associated targets threshold…”. Please restore the sentence and specify the significance threshold used, including whether it refers to nominal or adjusted p-values.

[Note: HTML markup is below. Please do not edit.]

[NOTE: If reviewer comments were submitted as an attachment file, they will be attached to this email and accessible via the submission site. Please log into your account, locate the manuscript record, and check for the action link "View Attachments". If this link does not appear, there are no attachment files.]

To ensure your figures meet our technical requirements, please review our figure guidelines: https://journals.plos.org/plosone/s/figures

You may also use PLOS’s free figure tool, NAAS, to help you prepare publication quality figures: https://journals.plos.org/plosone/s/figures#loc-tools-for-figure-preparation.

NAAS will assess whether your figures meet our technical requirements by comparing each figure against our figure specifications.

# Author comment: Author response to Decision Letter 5

**DOI:** 10.1371/journal.pone.0361766.r010

12 Oct 2026

PONE-D-26-23444R4

Unveiling the Neuroinflammatory Potential of Aspartame in Alzheimer's Disease: A Comprehensive Network Toxicology and Experimental Validation Approach

PLOS One

Dear Dr. Sheng,

Thank you for submitting your manuscript to PLOS One. After careful consideration, we feel that it has merit but does not fully meet PLOS One’s publication criteria as it currently stands. Therefore, we invite you to submit a revised version of the manuscript that addresses the points raised during the review process.

Please submit your revised manuscript by Nov 08 2026 11:59PM. If you will need more time than this to complete your revisions, please reply to this message or contact the journal office at plosone@plos.org. When you're ready to submit your revision, log on to https://www.editorialmanager.com/pone/ and select the 'Submissions Needing Revision' folder to locate your manuscript file.

Please include the following items when submitting your revised manuscript:

A letter that responds to each point raised by the academic editor and reviewer(s). You should upload this letter as a separate file labeled 'Response to Reviewers'.

A marked-up copy of your manuscript that highlights changes made to the original version. You should upload this as a separate file labeled 'Revised Manuscript with Track Changes'.

An unmarked version of your revised paper without tracked changes. You should upload this as a separate file labeled 'Manuscript'.

If you would like to make changes to your financial disclosure, please include your updated statement in your cover letter. Guidelines for resubmitting your figure files are available below the reviewer comments at the end of this letter.

If applicable, we recommend that you deposit your laboratory protocols in protocols.io to enhance the reproducibility of your results. Protocols.io assigns your protocol its own identifier (DOI) so that it can be cited independently in the future. For instructions see: https://journals.plos.org/plosone/s/submission-guidelines#loc-laboratory-protocols. Additionally, PLOS One offers an option for publishing peer-reviewed Lab Protocol articles, which describe protocols hosted on protocols.io. Read more information on sharing protocols at https://plos.org/protocols?utm_medium=editorial-email&utm_source=authorletters&utm_campaign=protocols.

As the corresponding author, your ORCID iD is verified in the submission system and will appear in the published article. PLOS supports the use of ORCID, and we encourage all coauthors to register for an ORCID iD and use it as well. Please encourage your coauthors to verify their ORCID iD within the submission system before final acceptance, as unverified ORCID iDs will not appear in the published article. Only the individual author can complete the verification step; PLOS staff cannot verify ORCID iDs on behalf of authors.

We look forward to receiving your revised manuscript.

Kind regards,

António Raposo

Academic Editor

PLOS One

Journal Requirements:

If the reviewer comments include a recommendation to cite specific previously published works, please review and evaluate these publications to determine whether they are relevant and should be cited. There is no requirement to cite these works unless the editor has indicated otherwise.

Please review your reference list to ensure that it is complete and correct. If you have cited papers that have been retracted, please include the rationale for doing so in the manuscript text, or remove these references and replace them with relevant current references. Any changes to the reference list should be mentioned in the rebuttal letter that accompanies your revised manuscript. If you need to cite a retracted article, indicate the article’s retracted status in the References list and also include a citation and full reference for the retraction notice.

Additional Editor Comments:

There are still a few passages that need attention before I can accept the manuscript.

(1) The main remaining issue is in the Discussion (page 20, lines 19–26). Although the wording about protein expression has been corrected, this paragraph still refers to “functional confirmation,” “downstream cytokines,” a “shared neuroinflammatory mechanism,” and effects occurring “through a GSK-3β-associated pathway.” These expressions go beyond what was measured. The experiments show an increase in GSK-3β mRNA alongside changes in inflammatory markers, but they do not establish that GSK-3β mediates those changes. Similarly, the resemblance to the LPS response does not demonstrate that the two treatments act through the same mechanism. Please revise this paragraph to present GSK-3β involvement as a possible explanation that requires further testing.

Response: We sincerely thank the Editor for this careful and important comment. The experiments show an increase in GSK-3β mRNA alongside changes in inflammatory markers, but they do not demonstrate that GSK-3β mediates those changes, and the resemblance to the LPS response does not prove that the two treatments act through the same mechanism. We have therefore revised this paragraph to present GSK-3β involvement as a possible explanation that requires further testing. Specifically, we replaced “functional confirmation” with “further investigation,” removed the term “downstream cytokines” and simply referred to the measured cytokines, changed the statement about a “shared neuroinflammatory mechanism” to a more cautious description of a similar direction of response without claiming a common mechanism, and replaced “through a GSK-3β-associated pathway” with a description of GSK-3β mRNA upregulation as a possible associated feature whose mediating role remains to be tested. The revised paragraph now reads as follows, with the changes highlighted in red (page 20, lines 21-30). We believe this revision keeps our interpretation strictly within the limits of the data and fully addresses the Editor’s concern.

(2) The same point applies to the phrase “provide a valid proof-of-concept for a mechanistic pathway” in the Limitations section (page 21, lines 25–27). Please describe this more cautiously as preliminary evidence of an inflammatory response accompanied by increased GSK-3β mRNA expression. The Conclusion should follow the same approach: “systematically elucidate” and “through a multi-target network in vitro” still suggest that the proposed mechanisms have been established experimentally.

Response: We sincerely thank the Editor for this further careful comment. We agree that the phrase “provide a valid proof-of-concept for a mechanistic pathway” in the Limitations section overstates what our data can support, and that the words “systematically elucidate” and “through a multi-target network in vitro” in the Conclusion still imply that the proposed mechanisms have been experimentally established. Our experiments show an inflammatory response accompanied by increased GSK-3β mRNA expression, but they do not establish a mechanistic pathway or a multi-target network in vitro. We have therefore revised both the Limitations and the Conclusion to describe our findings more cautiously as preliminary evidence of an inflammatory response accompanied by increased GSK-3β mRNA expression, and to present the proposed mechanisms as hypotheses that require further testing. All modified text has been highlighted in red (page 21, lines 28-29; page 22, lines 16, 25-26) in the revised manuscript. We are grateful to the Editor for helping us keep our conclusions strictly within the limits of our data.

(3) Please also check the molecular docking Results (page 12, lines 11–16). The sentence beginning “Our results demonstrated that aspartame forms…” should describe predicted interactions, and the discussion of binding affinities should make clear that these are computational estimates.

Response: We thank the Editor for this additional careful observation. In the original sentence, “Our results demonstrated that aspartame forms hydrogen bonds and strong electrostatic interactions with its protein targets” implied experimental observation, which docking cannot provide. Likewise, the listing of binding energies did not make clear that these values are calculated predictions. We have therefore revised this passage accordingly. The first sentence now reads: “Our docking analysis predicted that aspartame may form hydrogen bonds and electrostatic interactions with its protein targets.” The second sentence now reads: “The predicted binding energies of aspartame with CCNE1 (-4.049 kcal/mol), CDKN1A (-3.837 kcal/mol), CDKN1B (-3.356 kcal/mol), ERBB2 (-3.019 kcal/mol), GSK-3β (-5.002 kcal/mol), KDR (-5.613 kcal/mol), NOS3 (-4.046 kcal/mol), and VEGFA (-2.06 kcal/mol) are computational estimates that indicate varying binding affinities, with the most favorable predicted binding energies observed for GSK-3β and KDR and weaker predicted binding for VEGFA.” These changes make it clear that the docking results are predictions and that the binding energies are computational estimates, in line with the Editor’s comment. All modified text has been highlighted in red in the revised manuscript (page 12, lines 11-17). We are grateful to the Editor for helping us describe the docking results with appropriate precision.

(4) Finally, there is an incomplete sentence in the functional enrichment Methods (page 5, lines 19–21), beginning “Key pathways overlapping aspartame- and AD-associated targets threshold…”. Please restore the sentence and specify the significance threshold used, including whether it refers to nominal or adjusted p-values.

Response: We thank the Editor for catching this incomplete sentence. We apologize for the error. We have now restored the sentence and have also specified the significance threshold clearly, including whether it refers to nominal or adjusted p-values. In our KEGG enrichment analysis using the DAVID database, we used a Benjamini-Hochberg adjusted p-value < 0.05 as the threshold for identifying significantly enriched pathways. This is an adjusted p-value, not a nominal p-value. We have therefore revised the Methods sentence to read as follows, with the restored and clarified text highlighted in red: “Key pathways overlapping aspartame- and AD-associated targets were determined via KEGG enrichment analysis. A Benjamini–Hochberg adjusted p-value < 0.05 was used as the significance threshold to control the false discovery rate (FDR), and all visualizations were performed using the SRplot platform” This revision restores the grammatical structure of the sentence and makes explicit that the threshold refers to adjusted p-values (page 5, lines 20-22). All modified text has been highlighted in red in the revised manuscript. We are grateful to the Editor for identifying this error and for giving us the opportunity to correct it.

Attachment Submitted filename: Response_to_Reviewers_auresp_5.docx

# Editor report: Decision Letter 5

**DOI:** 10.1371/journal.pone.0361766.r011

**Authors:** António Raposo

16 Oct 2026

<p>Unveiling the Neuroinflammatory Potential of Aspartame in Alzheimer's Disease: A Comprehensive Network Toxicology and Experimental Validation Approach

PONE-D-26-23444R5

Dear Dr. Sheng,

We’re pleased to inform you that your manuscript has been judged scientifically suitable for publication and will be formally accepted for publication once it meets all outstanding technical requirements.

Within one week, you’ll receive an e-mail detailing the required amendments. When these have been addressed, you’ll receive a formal acceptance letter and your manuscript will be scheduled for publication.

An invoice will be generated when your article is formally accepted. Please note, if your institution has a publishing partnership with PLOS and your article meets the relevant criteria, all or part of your publication costs will be covered. Please make sure your user information is up-to-date by logging into Editorial Manager at Editorial Manager® and clicking the ‘Update My Information' link at the top of the page. For questions related to billing, please contact billing support.

If your institution or institutions have a press office, please notify them about your upcoming paper to help maximize its impact. If they’ll be preparing press materials, please inform our press team as soon as possible -- no later than 48 hours after receiving the formal acceptance. Your manuscript will remain under strict press embargo until 2 pm Eastern Time on the date of publication. For more information, please contact onepress@plos.org.

Kind regards,

António Raposo

Academic Editor

PLOS One

Additional Editor Comments (optional):

Reviewers' comments:

# Editor report: Acceptance letter

**DOI:** 10.1371/journal.pone.0361766.r012

**Authors:** António Raposo

PONE-D-26-23444R5

PLOS One

Dear Dr. Sheng,

I'm pleased to inform you that your manuscript has been deemed suitable for publication in PLOS One. Congratulations! Your manuscript is now being handed over to our production team.

At this stage, our production department will prepare your paper for publication. This includes ensuring the following:

* All references, tables, and figures are properly cited

* All relevant supporting information is included in the manuscript submission,

* There are no issues that prevent the paper from being properly typeset

You will receive further instructions from the production team, including instructions on how to review your proof when it is ready. Please keep in mind that we are working through a large volume of accepted articles, so please give us a few days to review your paper and let you know the next and final steps.

Lastly, if your institution or institutions have a press office, please let them know about your upcoming paper now to help maximize its impact. If they'll be preparing press materials, please inform our press team within the next 48 hours. Your manuscript will remain under strict press embargo until 2 pm Eastern Time on the date of publication. For more information, please contact onepress@plos.org.

You will receive an invoice from PLOS for your publication fee after your manuscript has reached the completed accept phase. If you receive an email requesting payment before acceptance or for any other service, this may be a phishing scheme. Learn how to identify phishing emails and protect your accounts at https://explore.plos.org/phishing.

If we can help with anything else, please email us at customercare@plos.org.

Thank you for submitting your work to PLOS One and supporting open access.

Kind regards,

PLOS One Editorial Office Staff

on behalf of

Dr. António Raposo

Academic Editor

PLOS One
